# Efficacy and safety of intravenous imatinib in COVID-19 ARDS: a randomized, double-blind, placebo-controlled clinical trial

**DOI:** 10.1186/s13054-023-04516-4

**Published:** 2023-06-08

**Authors:** Leila N. Atmowihardjo, Job R. Schippers, Erik Duijvelaar, Imke H. Bartelink, Pierre M. Bet, Noortje E. L. Swart, Nienke van Rein, Keith Purdy, David Cavalla, Andrew McElroy, Sarah Fritchley, Anton Vonk Noordegraaf, Henrik Endeman, Patricia van Velzen, Matty Koopmans, Harm Jan Bogaard, Leo Heunks, Nicole Juffermans, Marcus J. Schultz, Pieter R. Tuinman, Lieuwe D. J. Bos, Jurjan Aman

**Affiliations:** 1 Intensive Care, Amsterdam UMC Location University of Amsterdam, Meibergdreef 9, Amsterdam, The Netherlands; 2 Department of Pulmonary Medicine, Amsterdam University Medical Centers, Location VUmc, Room number 5A-074, De Boelelaan 1117, 1081 HV Amsterdam, The Netherlands; 3 Department of Clinical Pharmacology and Pharmacy, Amsterdam UMC Location Vrije Universiteit Amsterdam, Boelelaan 1117, Amsterdam, The Netherlands; 4 Department of Clinical Pharmacology and Pharmacy, Amsterdam UMC Location University of Amsterdam, Meibergdreef 9, Amsterdam, The Netherlands; 5 Department of Clinical Pharmacology and Pharmacy, Leiden UMC, Albinusdreef 2, Leiden, The Netherlands; 6 Exvastat, Cambridge, England; 7 Intensive Care, Erasmus University Medical Centre, Doctor Molewaterplein 40, Rotterdam, The Netherlands; 8 Intensive Care, Dijklander Hospital, Location Purmerend, Waterlandlaan 250, Purmerend, The Netherlands; 9 Intensive Care, OLVG Hospital Location Oost, Oosterpark 9, Amsterdam, The Netherlands; 10 Mahidol Oxford Tropical Medicine Research Unit (MORU), Mahidol University, Bangkok, Thailand; 11 Nuffield Department of Medicine, University of Oxford, Oxford, UK; 12 Department of Research and Development, Hamilton Medical AG, Chur, Switzerland; 13 Intensive Care, Amsterdam UMC Location Vrije Universiteit Amsterdam, Boelelaan 1117, Amsterdam, The Netherlands; 14 Laboratory of Translational Intensive Care, Erasmus University, Rotterdam, The Netherlands

**Keywords:** COVID-19, ARDS, Imatinib, Vascular permeability, Endothelial barrier dysfunction, Pulmonary edema

## Abstract

**Purpose:**

A hallmark of acute respiratory distress syndrome (ARDS) is hypoxaemic respiratory failure due to pulmonary vascular hyperpermeability. The tyrosine kinase inhibitor imatinib reversed pulmonary capillary leak in preclinical studies and improved clinical outcomes in hospitalized COVID-19 patients. We investigated the effect of intravenous (IV) imatinib on pulmonary edema in COVID-19 ARDS.

**Methods:**

This was a multicenter, randomized, double-blind, placebo-controlled trial. Invasively ventilated patients with moderate-to-severe COVID-19 ARDS were randomized to 200 mg IV imatinib or placebo twice daily for a maximum of seven days. The primary outcome was the change in extravascular lung water index (∆EVLWi) between days 1 and 4. Secondary outcomes included safety, duration of invasive ventilation, ventilator-free days (VFD) and 28-day mortality. Posthoc analyses were performed in previously identified biological subphenotypes.

**Results:**

66 patients were randomized to imatinib (n = 33) or placebo (n = 33). There was no difference in ∆EVLWi between the groups (0.19 ml/kg, 95% CI − 3.16 to 2.77, *p* = 0.89). Imatinib treatment did not affect duration of invasive ventilation (*p* = 0.29), VFD (*p* = 0.29) or 28-day mortality (*p* = 0.79). IV imatinib was well-tolerated and appeared safe. In a subgroup of patients characterized by high IL-6, TNFR1 and SP-D levels (n = 20), imatinib significantly decreased EVLWi per treatment day (− 1.17 ml/kg, 95% CI − 1.87 to − 0.44).

**Conclusions:**

IV imatinib did not reduce pulmonary edema or improve clinical outcomes in invasively ventilated COVID-19 patients. While this trial does not support the use of imatinib in the general COVID-19 ARDS population, imatinib reduced pulmonary edema in a subgroup of patients, underscoring the potential value of predictive enrichment in ARDS trials.

*Trial registration* NCT04794088, registered 11 March 2021. European Clinical Trials Database (EudraCT number: 2020-005447-23).

**Supplementary Information:**

The online version contains supplementary material available at 10.1186/s13054-023-04516-4.

## Introduction

Coronavirus disease 2019 (COVID-19) can lead to the development of acute respiratory distress syndrome (ARDS) [1]. Diffuse inflammatory damage of the alveolocapillary membrane [2] and subsequent alveolar-capillary hyperpermeability results in protein-rich pulmonary edema, eventually causing acute hypoxaemic respiratory failure [3, 4]. Patients with COVID-19 ARDS benefit from immune modulation by dexamethasone [5], interleukin-6 (IL-6) receptor inhibitors [6], Janus kinase (JAK) inhibitors [7] and monoclonal antibodies [8, 9]. However, there is no effective treatment that directly targets increased alveolar-capillary hyperpermeability [10].

The tyrosine kinase inhibitor imatinib attenuates vascular hyperpermeability and pulmonary edema in various preclinical in vitro and animal models of vascular leak [11-14]. Imatinib protects endothelial barrier integrity under inflammatory conditions by inhibiting the tyrosine kinase Arg/Abl2. Arg/Abl2 mediates endothelial barrier disruption by increasing integrin turnover and reducing endothelial cell–matrix interaction [12, 15]. Translating these findings into the clinical setting, the randomized, placebo-controlled, double-blind CounterCOVID trial examined the efficacy and safety of oral imatinib in hospitalized COVID-19 patients requiring supplemental oxygen therapy [16]. While the primary endpoint, time to discontinuation of supplemental oxygen and mechanical ventilation for more than 48 consecutive hours, was negative, oral imatinib reduced duration of invasive ventilation and length of intensive care unit (ICU) admission at 28-days and additionally reduced mortality in 90-day follow up [16, 17].

Clinical benefits of imatinib were predominantly observed in patients admitted to the intensive care unit (ICU). In a secondary analysis of the CounterCOVID trial, patients were characterized using plasma biomarkers for hierarchical clustering [18]. Three subphenotypes were identified. Notably, only patients classified into subphenotype 3, characterized by elevated levels of inflammatory cytokines and endothelial and epithelial injury biomarkers, showed a mortality benefit from imatinib treatment. These findings suggested moderation of the beneficial effect of imatinib through biological subphenotypes [18].

In this trial, a newly developed intravenous (IV) formulation was used to bypass gastrointestinal dysfunction often observed in critically ill patients. Critically ill patients often suffer from gastrointestinal dysfunction, which includes delayed gastric emptying and intestinal edema, which may delay drug uptake and drug exposure [19, 20]. The dosing scheme was based on preclinical evidence of the optimal protective effect of oral imatinib on the endothelial barrier (plasma concentrations of 2–10 μM) [11] and pharmacokinetic studies of IV imatinib [21]. The CounterCOVID trial supported the above-mentioned dosing, showing that treatment with 400 mg/day was sufficient to obtain target plasma levels [22].

The InventCOVID trial evaluated the efficacy and safety of IV imatinib in patients receiving invasive ventilation for moderate-to-severe COVID-19 ARDS. We hypothesized that IV imatinib would stabilize the alveolocapillary barrier and thereby reduce pulmonary edema, quantified by extravascular lung water index (EVLWi), a validated measure of pulmonary edema [23-25]. To our knowledge, this is the first translational trial to directly target and measure pulmonary vascular leak in patients with ARDS.

## METHODS

### Study design and population

**Fig. 1:**
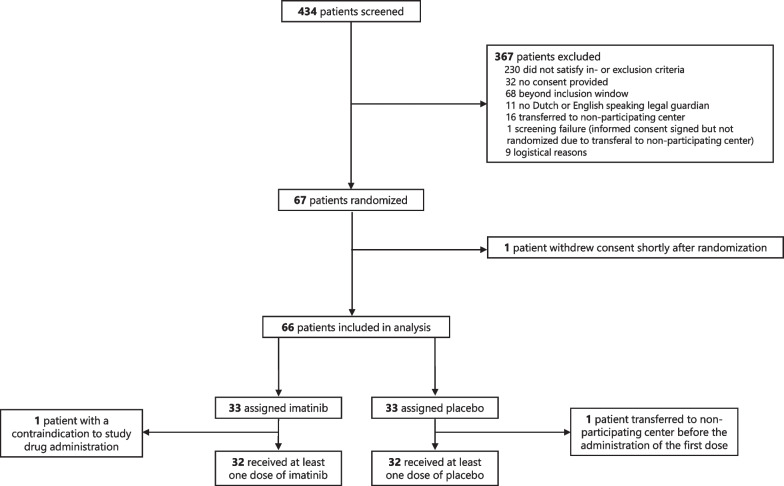
Patient screening and inclusion. Screening and inclusion flowchart. Of the 67 randomized patients, 66 patients were included in the final analysis and 64 patients received at least one dose of the study medication

This phase IIb, randomized, double-blind, placebo-controlled clinical trial was performed in four academic and non-academic ICUs in the Netherlands between March 2021 and March 2022 (Fig. 1, Additional file 1: Figure S1). The trial protocol was approved by the Medical Ethics Committee of the Amsterdam UMC (location VUMC, IRB number NL75871.029.20, approved on 22-01-2021) and has been published [26]. Written informed consent was obtained from the patient’s legal representative. The trial was conducted according to the principles of the World Medical Association’s Declaration of Helsinki.

Patients were eligible if aged ≥ 18 years, intubated for invasive ventilation and had moderate-to-severe ARDS due to COVID-19. ARDS was classified according to Berlin criteria [27]. The patients were included in the trial as soon as possible after intubation and were excluded from participation if the anticipated start of study medication was > 48 h after the start of invasive ventilation. Other exclusion criteria included a history of severe chronic pulmonary disease, ejection fraction of < 40% and participation in another clinical trial. A complete overview of all exclusion criteria can be found in Additional file 1 (p. 2).

### Randomization and blinding

Patients were randomized 1:1 to receive IV imatinib or placebo for 7 days. Randomization was done in the web-based application Castor electronic data capture using variable block sizes (4–6 patients per block) and stratification per participating center. Allocation of randomization group was only visible to the pharmacy staff preparing the treatment. The patients, clinical staff, investigators and statisticians remained blinded to randomization allocation during the entire study. Blinding was guaranteed by distribution of the study drug in amber-colored syringes and lines to conceal any color differences. The local pharmacies were responsible for the preparation of the study drug and, if necessary, for unblinding.

### Study procedures

At baseline, all patients received a Pulse Contour Cardiac Output (PiCCO; Pulsion Medical Systems, Munich, Germany) catheter. PiCCO catheter placement was performed as part of a deferred consent procedure and replaced the standard care arterial line. Extravascular lung water (EVLW) measurements were performed daily for seven days or until transfer to the ward, as previously described [28, 29]. EVLW was indexed to predicted body weight.

After randomization and PiCCO measurement, the study drug was administered twice daily as a 25 ml, two-hour infusion for seven days or until ICU discharge. The study drug consisted of a 9.6 mg/mL imatinib mesylate solution (Exvastat Ltd, Cambridge, United Kingdom), corresponding to 200 mg imatinib per dose, or placebo. Clinical and ventilation parameters were recorded on days 1,2,4,7,10 and 28. Ventilation parameters were recorded once per hour, and partial oxygen pressure (PaO2) at least once per shift (i.e. every 4 h) or more frequently, if clinically indicated. Ventilation parameters were collected at the time of the lowest ratio of PaO2 to the fraction of inspired oxygen (FiO2). Blood samples were collected for plasma biomarker analyses (Additional file 1: Table S1).

Adverse events (AEs) and serious adverse events (SAEs) were recorded until day 28. Due to the high incidence of adverse events in the ICU, only prespecified events were recorded (Additional file 2, pp. 26–29). Reporting was conducted according to the Council for International Organizations of Medical Sciences (CIOMS) reporting guidelines. Safety was assessed using clinical laboratory tests and electrocardiograms (ECG) at baseline and on days 1,2,4,7 and 10. Details on predefined stopping criteria, are described in the protocol (Additional file 2, pp. 24–25). Patients discharged before day 28 were contacted by telephone on day 28 to evaluate their clinical status.

### Prespecified outcomes

The primary endpoint was the change (Δ) in EVLWi between days 1 and 4. We chose this period to capture the effect of imatinib during the exudative phase of ARDS, characterized by vascular leak and alveolar flooding [3, 30, 31]. We hypothesized that this period would be the window-of-opportunity in which imatinib could best exert its vasculoprotective effect.

The following key secondary outcomes were analyzed in hierarchical order: change in P_a_O_2_/FiO_2_ ratio, number of ventilator-free days (VFD), length of ICU, hospital length of stay and 28-day mortality. Other explorative secondary endpoints included the duration of invasive ventilation, change in the pulmonary vascular permeability index (PVPi) and plasma biomarker concentrations (Additional file 1: Table S1), ventilation parameters, Sequential Organ Failure Assessment (SOFA) score and the 9-point World Health Organization (WHO) ordinal scale for clinical improvement (all specified in Additional file 1).

### Sample size calculation

A sample size of 90 patients was determined to demonstrate a 25% reduction in EVLWi, with 80% power and a type 1 error rate of 0.05. The calculation was based on an anticipated baseline EVLWi of 17 ml/kg with a standard deviation (SD) of 7 ml/kg. This was based on previously performed EVLW measurements in patients with moderate to severe ARDS [32-34]. The expected EVLWi reduction of 25% was based on preclinical data [11].

The study protocol (Additional file 2) allowed for a sample size re-estimation in case of falling recruitment rates. Revision of the power calculation was supported by a lower variation in EVLWi than initially anticipated based on data in non-COVID-19 ARDS (i.e. SD of 4.9 instead of 7.0). Re-estimation was done by updating the assumptions using pre-randomization data from the 66 patients included at the time of re-estimation. The estimated difference between groups (µ1 – µ2) was left unchanged, as we were not able to re-estimate the treatment effect without unblinding. Based on the observed mean EVLWi at n = 66 of 16.5 ml/kg with an SD of 4.9 and assuming an alpha of 5%, 19 patients per allocated group were considered sufficient to detect a 25% reduction in EVLWi between the groups at 80% power. Therefore, recruitment was halted after 67 patients.

### Statistical analysis

Statistical analyses were performed in the intention-to-treat (ITT) population of 66 randomized patients. An overview of predefined statistical tests is provided in the Statistical Analysis Plan (Additional file 3). In summary, normal distribution was tested using the Shapiro–Wilk test. Baseline imbalances were defined as a difference of ≥ 5% between the placebo and imatinib groups, and baseline imbalances deemed clinically relevant to the primary outcome by consensus were adjusted for in subsequent analyses. Categorical data were expressed as numbers and percentages, and differences between categorical variables were tested using a Chi-square test. Continuous data were expressed as mean ± SD or median with interquartile range [IQR]. Differences between continuous variables were analyzed depending on parametric or non-parametric distribution using a two-tailed t-test, or Mann–Whitney-U-test, respectively.

The primary endpoint was presented as mean ± SD and analyzed using a two-tailed t-test. In addition, an analysis of covariance (ANCOVA) was performed. For the primary endpoint, data imputation was performed in case of missing EVLWi values. In case of missing values on day 1, the data from day 2 was carried backwards. In case of missing values at day 4, the value was imputed by carrying the day 3 value forward, or, in case of no day 3 measurement, carrying the day 5 value backwards. For sensitivity analysis, the primary endpoint was analyzed in the ITT population without data imputation. In addition, a per-protocol analysis of the primary endpoint was performed in all patients missing ≤ 1 study drug dose in the first four study days and who had no missing EVLWi measurements on days 1 and 4.

28-day mortality was analyzed using a Cox proportional hazards model and visualized using a Kaplan–Meier curve. EVLWi, PVPi, ventilation parameters, plasma biomarkers and clinical laboratory outcomes over the first seven days were analyzed using linear mixed effect (LME) models, with time point, randomization group and their interaction term as fixed effects, study ID as random intercept and, in case of baseline imbalances, relevant covariates. For LME analyses, logarithmic transformation was applied to non-normally distributed data.

We performed a posthoc analysis applying the three biomarker-derived subphenotypes identified in the CounterCOVID trial [18]. Using the *nnet* package [35], a multinomial logistic regression model was trained using data from the CounterCOVID trial with plasma levels of interleukin (IL)-6, tumor necrosis factor receptor 1 (TNFR1), surfactant protein (SP)-D and angiopoietin (Ang)-2 to Ang-1 ratio measured at baseline as predictors of cluster allocation. Based on pre-treatment biomarker data obtained from the InventCOVID patients and using the *predict()* function in R studio [36], this model was used to classify patients into three subphenotypes: i.e. subphenotype 1 (high IL-6, high TNFR1, low SP-D), subphenotype 2 (low IL-6, low TNFR1, low SP-D) and subphenotype 3 (high IL-6, high TNFR1, high SP-D). LME modelling of ΔEVLWi per treatment day was repeated in the subphenotypes. Statistical analyses were performed using R, version 4.1.3 and RStudio, version 2022.02.1.

## Results

### Patient characteristics

Between March 2021 and March 2022, 434 patients admitted to the ICU were screened for trial participation. Of these, 67 patients underwent randomization. One patient withdrew consent shortly after randomization, before receiving the first dose of study medication, and was excluded from further analysis. The study medication was not administered in two patients because of a contraindication to study drug administration and transferal to a non-participating hospital before administration of the first dose. Altogether, the ITT population contained 33 patients per arm (Fig. 1).

Baseline patient characteristics were largely balanced between the two groups. Imbalances in age, BMI and d-dimer levels were deemed clinically relevant (Table 1) and adjusted for in the analysis of primary and secondary outcomes. 53 patients (80%) were classified as moderate ARDS and 13 patients (20%) as severe ARDS. All patients received dexamethasone treatment and 91% received IL-6 receptor inhibitors (Table 1). Additionally, 15 patients (23%) received monoclonal antibodies (Additional file 1: Table S2). The median duration of study treatment was 7 days [4, 7]. The most common reasons for study drug discontinuation were transferal to a non-participating hospital and discharge to the ward before study day 7 (Additional file 1: Table S3).

**Table 1:** Baseline demographic and clinical patient characteristics

	**Placebo**	**Imatinib**
n	33	33
*Admission characteristics*		
Age in years, mean (SD)	62 (11.4)	63 (9.3)
Male sex, n (%)	19 (58)	19 (58)
BMI in kg/m^2^, median [IQR]	30 [28, 35]	28 [25, 32]
ARDS classification (Berlin criteria)		
Severe, n (%)	6 (18)	7 (21)
Moderate, n (%)	27 (82)	26 (79)
SOFA score, median [IQR]	8 [7.0, 8.0]	7 [6.8, 9.0]
Charlson comorbidity score, median [IQR]	2 [1, 4]	2 [1, 4]
Fluid balance in liters, median [IQR]	0.46 (1.01)	0.12 (1.3)
QTc in msec, mean (SD)	451.0 (31.6)	447.8 (29.6)
IL-6 receptor inhibitors*, n (%)	30 (91)	30 (91)
Dexamethasone, n (%)	31 (94)	30 (91)
Completed 10-day dexamethasone treatment before ICU admission, n (%)	2 (6)	3 (9)
Vaccinated, n (%)		
No	19 (58)	14 (42)
Yes	6 (18)	11 (33)
Unknown	8 (24)	8 (24)
Time from symptoms until intubation in days, median [IQR]	11 [8, 15]	12 [9, 13]
*Comorbidities*^†^		
COPD, n (%)	1 (3)	0 (0)
Heart failure, n (%)	0 (0)	0 (0)
Renal failure, n (%)	2 (6)	2 (6)
Myocardial infarction, n (%)	1 (3)	2 (6)
*Ventilation and gas exchange*		
TV/PBW in ml/kg, median [IQR]	6.2 [5.8, 7.2]	6.2 [5.6, 7.5]
PaO_2_/FiO_2_ in mmHg, mean (SD)	138 (35.8)	130 (34.9)
PEEP in cmH_2_O, mean (SD)	11.2 (2.3)	10.6 (2.8)
*Laboratory measurements*		
Hemoglobin in g/dL, median [IQR]	13.5 [13.1, 13.9]	13.1 [12.3, 13.9]
Leucocytes × 10^9^/L, median [IQR]	10.8 [7.7, 14.6]	10.1 [8.4, 16.2]
Thrombocytes × 10^9^/L, median [IQR]	267 [223, 348]	265 [227, 367]
D-dimer in mg/L, median [IQR]	1.7 [0.9, 4.0]	3.5 [1.3, 12.7]
Creatinine in micromol/L, median [IQR]	90 [65, 108]	84 [71, 121]
NTproBNP in pg/ml, median [IQR]	140 [62, 424]	169 [74, 471]
AST in U/L, median [IQR]	56 [48, 88]	53 [38, 77]
ALT in U/L, median [IQR]	57 [40, 114]	41 [27, 62]
*PiCCO measurements*		
EVLW in ml, median [IQR]	1039.0 [884.5, 1185.8]	1026.5 [849.3, 1363.5]
EVLWi in ml/kg, median [IQR]	15.2 [12.8, 18.5]	15.3 [13.7, 19.5]
PVPi, median [IQR]	3.1 [2.6, 3.9]	3.7 [2.8, 4.7]

*ALT* alanine transaminase; *ARDS* Acute Respiratory Distress Syndrome; *AST* aspartate transaminase; *BMI* Body Mass Index; *COPD* chronic obstructive pulmonary disease; *COVID-19* Coronavirus disease 2019; *EVLW(i)* extravascular lung water (index); *FiO*_*2*_ fraction of inspired oxygen; *ICU* intensive care unit; *IL-6* interleukin-6; *IQR* interquartile range; *NTproBNP* N-terminal prohormone brain natriuretic peptide; *PaO*_*2*_ partial pressure of oxygen; *PCR* Polymerase chain reaction; *PEEP* positive end-expiratory pressure; *PiCCO* pulse contour cardiac output; *PVPi* pulmonary vascular permeability index. *QT*_*c*_ corrected QT interval time; *SARS-CoV-2* Severe acute respiratory syndrome coronavirus 2; *SD* standard deviation; *SOFA* Sequential Organ Failure Assessment; *TV/PBW* tidal volume indexed to predicted body weight; *PVPi* pulmonary vascular permeability index

^*^Tocilizumab (8 mg/kg single intravenous administration) or sarilumab (400 mg single intravenous administration) administered upon Intensive Care Unit admission

^†^Known history of the disease at the moment of randomization

### Primary outcome

**Fig. 2:**
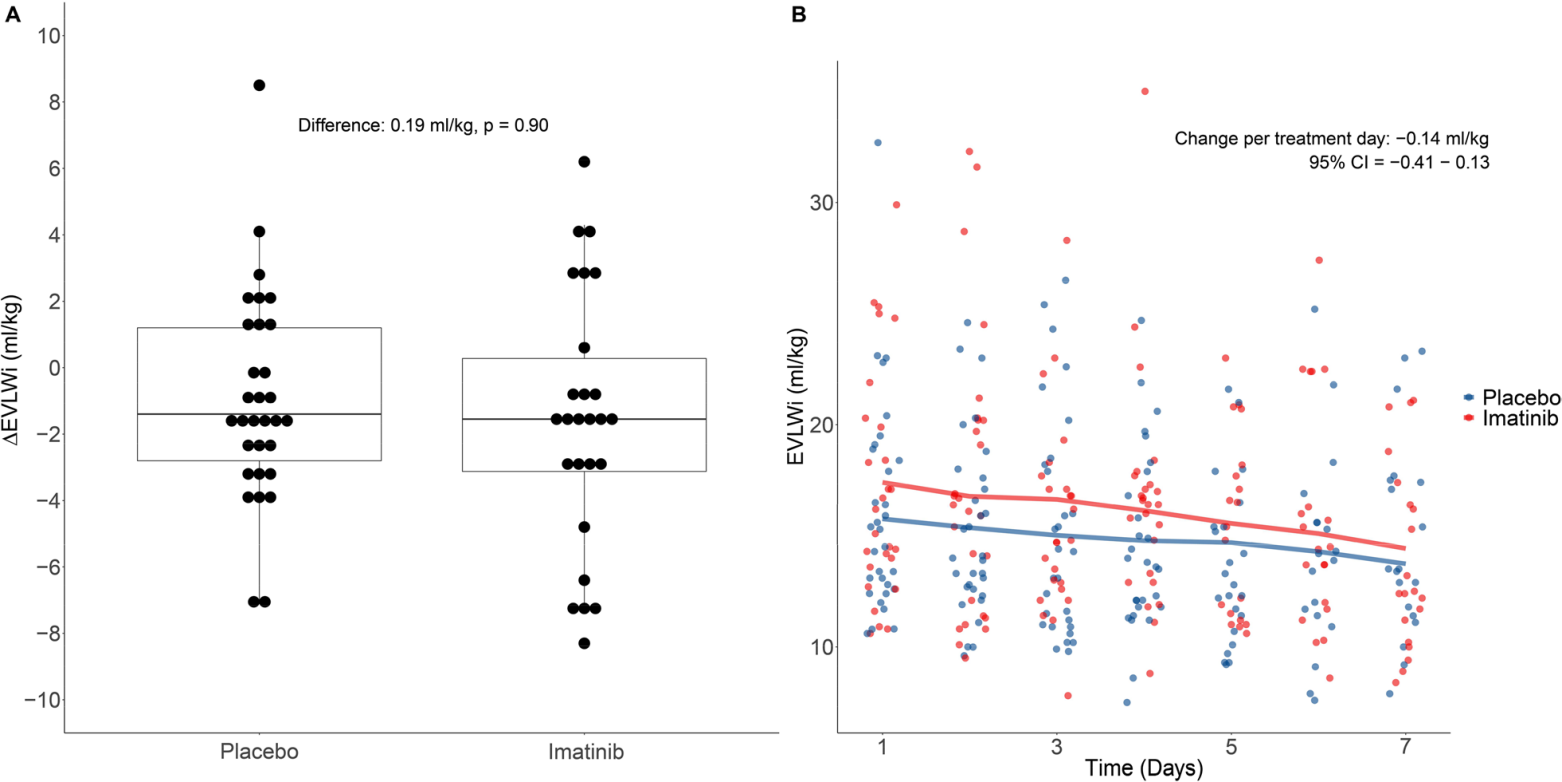
The primary endpoint and extravascular lung water index (EVLWi) over time, stratified by randomization group. Boxplot (**A**) depicting the distribution of the change in EVLWi in the imatinib group versus the placebo group between baseline (day 1) and day 4. Scatterplot (**B**) depicting the dynamic changes of the EVLWi per treatment day in the imatinib group versus the placebo group from day 1 to day 7

No significant difference in mean ∆EVLWi between days 1 and 4 was found between the two groups (difference 0.19 ml/kg, 95% CI − 3.16 to 2.77, *p* = 0.90, Table 2, Fig. 2A). This finding was confirmed by ANCOVA using the randomization group and individual patients as covariates (*p* = 0.39, Table 2). Four patients (6%) did not have a day 1 EVLWi measurement and 10 patients (15%) did not have a day 4 measurement. These values could be imputed from a previous or subsequent day in 7 of the 14 patients (50%). Sensitivity analysis without imputation of missing EVLWi data comprising 52 patients (78%) and the per-protocol analysis containing 48 patients (72%) both did not reveal significant differences in ∆EVLWi (Table 2).

**Table 2:** Trial efficacy endpoints: Change in extravascular lung water index between days 1 and 4 (primary outcome)

Population	Test	Placebo	Imatinib	Effect size	t/F-value	df	95% CI	*P* -value
ITT (n = 66)	T-test	− 1.37	− 1.18	0.19	− 0.13	41.63	− 3.16 to 2.77	0.90
PP (n = 49)	T-test	− 1.35	− 1.34	0.01	− 0.00	32.32	− 3.70 to 3.71	0.99
Sensitivity (n = 52)	T-test	− 1.34	− 1.17	0.17	− 0.10	35.07	− 3.64 to 3.30	0.92
ITT	ANCOVA	–	–	–	0.76	1	–	0.39

Table summarizing the primary endpoint analysis in the intention-to-treat (ITT) and per-protocol (PP) population, including the sensitivity analysis without imputation of missing data

*ANCOVA* analysis of covariance; *CI* confidence interval; *df* degrees of freedom, *EVLWi* extravascular lung water index in ml/kg; *ITT* intention-to-treat; *PP* per-protocol

### Secondary outcomes

**Fig. 3:**
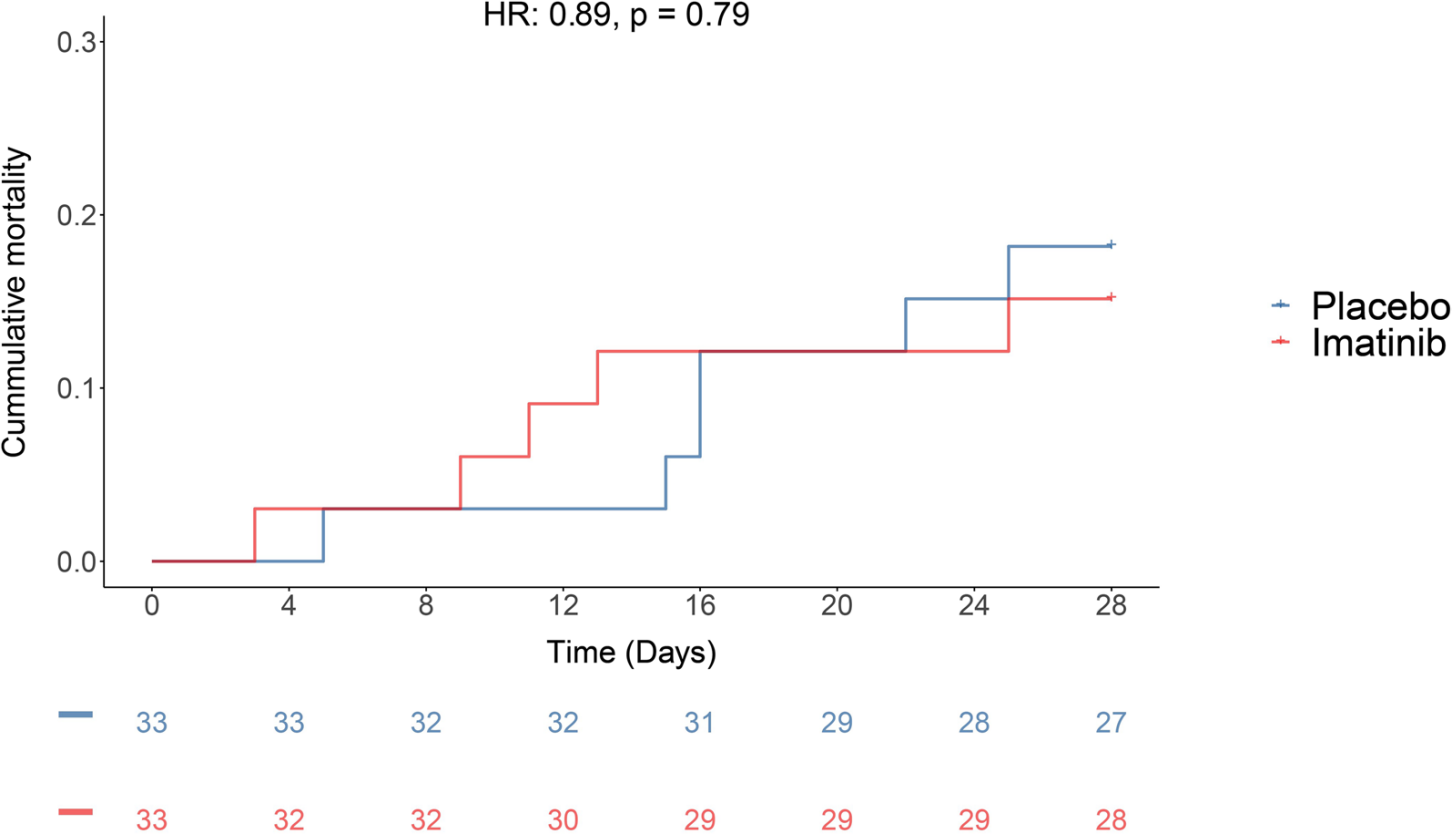
Kaplan-Meier curve of the 28-day mortality rate. Kaplan–Meier curve depicting time-to-event analysis of the 28-day mortality rate in patients treated with imatinib and placebo. The unadjusted hazard ratio was calculated by Cox regression analysis

The median number of VFDs was 14 days [0, 23] in the imatinib group versus 19 days [0, 24] in the placebo group (*p* = 0.29) (Table 3). There was no significant difference in the median duration of invasive ventilation, ICU stay and hospital stay (Table 3). The unadjusted hazard ratio for mortality was 0.89 (95% CI 0.39–2.07, *p* = 0.79, Fig. 3). No difference in PaO_2_/FiO_2_ ratio, EVLWi, PVPi, oxygenation index, driving pressure, compliance, mechanical power, SOFA score or 9-point WHO ordinal scale for clinical improvement was observed between groups (Table 4, Fig. 2B; Additional file 1: Figure S2, S3 and S6).

**Table 3:** Trial efficacy endpoints: Key secondary outcomes

	Placebo	Imatinib	Effect size (95% CI)	*p* value
28-day mortality, n (%)	6 (18)	5 (15)	HR: 0.89 (0.39 to 2.07)	0.79
VFD, median days [IQR]*	19 [0, 24]	14 [0, 23]	HL: 1.00 (− 0.99 to 9.00)	0.29
Sensitivity analysis^†^	19 [0, 24]	14 [0, 23]	HL: 1.00 (− 1.00 to 9.00)	0.36
Duration of ICU stay, median days [IQR]^‡^	11 [6, 28]	23 [7, 28]	HL: 0.00 (− 0.11 to 0.00)	0.25
Hospital length of stay, median days [IQR]^‡^	21 [13, 28]	28 [13, 28]	HL: − 2.00 (− 8.00 to 0.00)	0.20

Table summarizing the secondary outcomes at 28 days

*HL* Hodges—Lehmann estimator; *HR* Hazard ratio; *ICU* intensive care unit; *IQR* interquartile range; *VFD* ventilator-free days

^*^Death penalized by assigning 0 days

^†^Death penalized by assigning -1 days

^‡^Death penalized by assigning 28 days

**Table 4:** Trial efficacy endpoints: P_a_O_2_/FiO_2_ ratio and exploratory outcomes

Population	Outcome	Change per day exposed to treatment	95% CI
ITT	PaO_2_/FiO_2_*	− 0.09	− 4.11 to 4.01
ITT	EVLWi	− 0.14	− 0.41 to 0.13
PP	EVLWi	− 0.68	− 1.39 to 0.017
ITT	Oxygenation index	+ 0.32	− 0.21 to 0.83
ITT	Driving pressure	+ 0.31	− 0.21 to 0.83
ITT	Compliance	− 1.54	− 4.66 to 1.54
ITT	Mechanical power	+ 0.54	− 0.40 to 1.46
ITT	PVPi	− 0.05	− 0.12 to 0.01
ITT	SOFA score	− 0.08	− 0.32 to 0.16

Table summarizing the secondary outcomes assessed by linear mixed effect modelling, with the placebo group as the reference group

*CI* confidence interval; *EVLWi* extravascular lung water index in ml/kg; *ITT* intention-to-treat; *PaO*_*2*_*/FiO*_*2*_ partial pressure of oxygen to fraction of inspired oxygen (FiO_2_) ratio; *PP* per-protocol; *PVPi* pulmonary vascular permeability index; *SOFA* sequential organ failure assessment

^*^Prioritized secondary outcome

### Safety

The reported AEs and SAEs are listed in Table 5. AEs and SAEs were recorded in 18 imatinib-treated patients and 17 placebo-treated patients. 16 patients (50%) in the imatinib group and 11 patients (34%) in the placebo group had at least one SAE. The most frequently reported AEs were pulmonary embolism and bacteremia. The most frequently reported SAE was respiratory failure. Respiratory failure events were reported more frequently in the imatinib group (11 versus four patients, respectively). Four cases of respiratory failure in the imatinib group occurred after study day 28 but were reported in adherence to the European Commission’s CT3 guideline. In 18 patients, the final outcome of a reported SAE was death, with 10 deaths in the imatinib group and eight deaths in the placebo group. No infusion reactions due to imatinib administrations were observed and no AEs or SAEs were attributed to imatinib treatment.

**Table 5:** Treatment-emergent adverse and serious adverse events, stratified by treatment group

	AEs			SAEs		
	Placebo n = 32	Imatinib n = 32	Total n = 64	Placebo n = 32	Imatinib n = 32	Total n = 64
Any treatment-emergent adverse events, n (%)	16 (50)	18 (56)	34 (53)	11 (34)	16 (50)	27 (42)
Anemia	0 (0)	0 (0)	0 (0)	2 (6)	1 (3)	3 (5)
Fatal outcome*, n (%)	0 (0)	0 (0)	0 (0)	8 (25)	10 (31)	18 (28)
*Respiratory disorders*						
Bacterial pneumonia, n (%)	4 (13)	0 (0)	4 (6)	0 (0)	0 (0)	0 (0)
Viral pneumonia (herpes), n (%)	1 (3)	0 (0)	1 (2)	0 (0)	0 (0)	0 (0)
Aspiration pneumonia, n (%)	1 (3)	0 (0)	1 (2)	0 (0)	0 (0)	0 (0)
Fungal pneumonia, n (%)	2 (6)	3 (9)	5 (7.8)	1 (3)	0 (0)	1 (2)
Pneumonia unknown pathogen, n (%)	1 (3)	1 (3)	2 (3)	0 (0)	0 (0)	0 (0)
Respiratory failure, n (%)^†^	1 (3)	0 (0)	1 (2)	4 (13)	11 (34)	16 (23)
Pneumothorax, n (%)	0 (0)	1 (3)	1 (2)	0 (0)	0 (0)	0 (0)
Pulmonary embolism, n (%)	3 (9)	6 (19)	9 (14)	0 (0)	0 (0)	0 (0)
Laryngeal edema, n (%)	0 (0)	0 (0)	0 (0)	0 (0)	1 (3)	1 (2)
*Infections*						
Bacteremia, n (%)	3 (9)	5 (16)	8 (3)	0 (0)	0 (0)	0 (0)
Infection of unknown origin, n (%)	1 (3)	4 (13)	5 (8)	0 (0)	0 (0)	0 (0)
Sepsis, n (%)	0 (0)	1 (3)	1 (2)	0 (0)	0 (0)	0 (0)
Septic shock, n (%)	0 (0)	0 (0)	0 (0)	4 (13)	2 (6)	6 (9)
Ischemic stroke, n (%)	0 (0)	0 (0)	0 (0)	1 (3)	0 (0)	1 (2)
*Vascular*						
Arterial thrombosis, n (%)	0 (0)	1 (3)	1 (2)	0 (0)	0 (0)	0 (0)
Retroperitoneal hemorrhage, n (%)	0 (0)	0 (0)	0 (0)	1 (3)	0 (0)	1 (2)
*Cardiac disorders*						
Hyperkaliemia-induced cardiac arrest, n (%)	0 (0)	0 (0)	0 (0)	1 (3)	0 (0)	1 (2)
Life-threatening arrhythmia, n (%)	0 (0)	0 (0)	0 (0)	0 (0)	1 (3)	1 (2)
Right ventricular failure, n (%)	0 (0)	0 (0)	0 (0)	0 (0)	1 (3)	1 (2)
Renal replacement therapy initiation, n (%)	0 (0)	0 (0)	0 (0)	1 (3)	4 (13)	5 (8)
Muscle necrosis, n (%)	0 (0)	0 (0)	0 (0)	1 (3)	0 (0)	1 (2)
Severe dyskinesia, n (%)	0 (0)	0 (0)	0 (0)	1 (3)	0 (0)	1 (2)
Skin rash, n (%)	1 (3)	1 (3)	2 (3)	0 (0)	0 (0)	0 (0)

Table summarizing the occurrence of adverse and serious treatment-emergent adverse events and the frequency of fatal outcomes of patients treated with imatinib or placebo (n = 64)

*AEs* adverse events; *SAEs* serious adverse events

^*^7 deaths were reported beyond study day 28 in adherence with the CT3 guidelines of reporting adverse events in medicinal studies with human subjects. ^†^4 cases of respiratory failure were reported beyond study day 28 (4 in the imatinib group, 0 in the placebo group)

Laboratory test results over time are displayed in Table S4 and Figure S4 (Additional file 1). There was no difference in hemoglobin, liver function and kidney function between the groups. Patients receiving imatinib showed a larger increase in leucocyte count over time. No difference in the course of NT-proBNP or QTc time was observed between the groups.

### Posthoc analysis

**Fig. 4:**
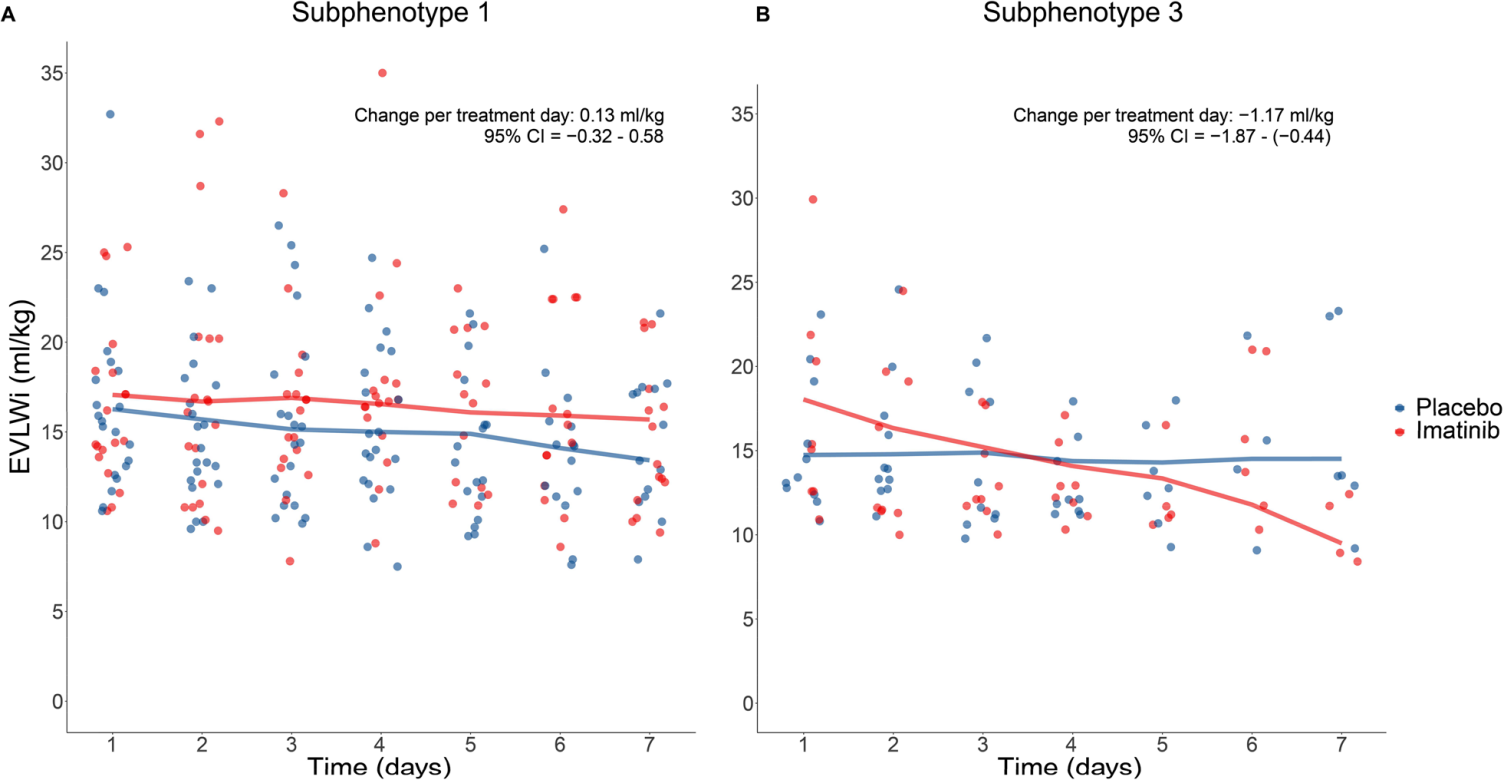
The change in extravascular lung water index over time, stratified by treatment group in subphenotypes 1 and 3. Scatterplots depicting the dynamic changes of the extravascular lung water index (EVLWi) per treatment day in the imatinib group versus the placebo group from day 1 to day 7 in subphenotype 1 and subphenotype 3

Biomarker analyses are provided in Table S5 and Figure S5 (Additional file 1). Based on the three subphenotypes identified in the CounterCOVID trial, 43 patients were classified into subphenotype 1, 20 patients into subphenotype 3 and none in the milder subphenotype 2 Three patients had no baseline plasma biomarker measurements and could not be assigned. Subphenotype characteristics and biomarker levels can be found in Table S6, S7 and S8 (Additional file 1). In the LME analysis modelling EVLWi over the 7 treatment days, a significant difference was found in patients classified into subphenotype 3 receiving imatinib (∆EVLWi per treatment day, with placebo as the reference group: − 1.17 ml/kg, 95% CI − 1.87 to − 0.44, Fig. 4B). In subphenotype 1, no significantly different change in EVLWi over time was observed between the two groups (Fig. 4A).

## Discussion

Administration of IV imatinib in invasively ventilated patients with COVID-19 ARDS had no significant effect on ΔEVLWi between days 1 and 4, indicating that imatinib did not reduce pulmonary edema. Imatinib did not improve clinical outcomes such as oxygenation, duration of invasive ventilation or 28-day mortality. IV imatinib was well-tolerated, with no adverse events attributed to imatinib administration. A posthoc analysis in previously identified subphenotypes suggests that IV imatinib reduced EVLWi in a subgroup of patients characterized by high levels of IL-6, TNFR1 and SP-D.

This trial follows up on the CounterCOVID trial, which showed that oral imatinib, in hospitalized COVID-19 patients, resulted in a shorter duration of invasive ventilation and a lower 90-day mortality [16, 17]. There are several possible explanations why imatinib did not reduce pulmonary edema and did not improve clinical outcomes in the current study. The first explanation comprises the underlying heterogeneity of COVID-19 ARDS [37]. By applying previously identified subphenotypes [18], we aimed to investigate whether the biological heterogeneity found in the CounterCOVID trial may explain the absence of an effect observed in the current trial. While the sample size of the subphenotype groups is limited, subgroup analysis revealed a significant reduction in EVLWi one biological subphenotype, suggesting that predictive enrichment using ARDS severity classification was insufficient. Notably, a minority of patients were classified into this subphenotype, which may explain the overall neutral finding of the trial.

Second, a notable difference with the CounterCOVID trial was that in the current trial, virtually all patients received IL-6 receptor inhibitors before starting imatinib treatment. Although attenuation of pulmonary endothelial barrier disruption was the hypothesized working mechanism of imatinib, an immune-modulating effect was previously observed [12, 18, 38, 39]. Potentially, this beneficial effect was already achieved by concomitant treatment with immunomodulatory drugs, making it more challenging to find an additional effect of imatinib. An argument against this is that imatinib treatment did not result in a decrease in IL-6 levels, providing no direct evidence for immune modulation through IL-6 reduction. It is unlikely that a potential difference in IL-6 levels was masked by the use of IL-6 receptor blockers, as these tend to cause an increase in IL-6 concentrations [40, 41].

Third, the timing of treatment may have been too late. Imatinib may not be able to effectively reverse vascular leak and pulmonary edema once the alveolocapillary barrier has suffered excessive damage. The high baseline EVLWi and PVPi levels suggest that the peak in pulmonary edema formation likely occurred before intubation. This could explain the discrepancy with the beneficial clinical effect seen in the CounterCOVID ICU population, in whom imatinib treatment was started within 24 h after hospital admission [16]. We thus suggest that optimal start of alveolar endothelial barrier-enhancing drugs is before intubation.

Fourth, autopsy studies in COVID-19 patients have revealed extensive endothelial injury [42], the relative contribution of endothelial dysfunction to pulmonary edema development and clinical outcome may be outweighed by extensive inflammation and alveolar epithelial cell injury [43, 44]. Lastly, although the daily dosing in the current study was similar to that in the CounterCOVID trial (400 mg/day), pharmacokinetic analysis of the CounterCOVID data demonstrated that the free fraction of imatinib inversely correlates with acute-phase proteins [22]. The pharmacokinetics of imatinib may thus be perturbed in conditions of critical illness, potentially affecting the dose–response relationship in the current study population.

This was the first trial to intravenously administer imatinib and evaluate its safety. While no clear safety concerns were observed, a critical view of the data could indicate a trend towards more (serious) adverse events in the imatinib group. However, these differences were not statistically significant, and none of the AEs could be attributed to the treatment of imatinib. A larger trial is necessary to provide a definitive answer to the question of safety of IV imatinib in critically ill patients.

A strength of this trial was that by using EVLWi as a measure of pulmonary edema, we could directly translate the preclinical hypothesis of the effect of imatinib on vascular leak to measurement in the clinical setting. EVLWi is a validated measure of pulmonary edema and an independent prognostic factor in patients with ARDS [29, 45]. Moreover, COVID-19 ARDS is characterized by higher EVLWi than non-COVID ARDS [46]. Thus, despite the neutral findings of this trial, we remain confident that it was a well-chosen endpoint for a phase IIb study investigating the efficacy of a drug targeting pulmonary vascular hyperpermeability. Another strength of the study design is the collection of a comprehensive array of parameters to study vascular leak in ARDS, such as transpulmonary thermodilution, ventilation parameters and plasma biomarker data. This provides an unprecedented biological and clinical characterization of vascular leak in ARDS patients. Moreover, this trial was conducted in a period in which dexamethasone [5], IL-6 receptor inhibitors [6] and monoclonal antibodies [9] had already been implemented for the management of COVID-19, thus reflecting current pharmacotherapeutic practice.

Some limitations should be acknowledged. EVLW measurements can be affected by external and patient factors, such as ventilator settings [47] and rhythm disturbances [47], potentially causing inaccuracies. Moreover, imputation of missing EVLWi data may have introduced over-or underestimation. Attrition due to transferal to non-participating centers in the context of the pandemic lead to a relatively high rate of patients in whom the study drug had to be prematurely discontinued. Lastly, concomitant treatment with IL-6 receptor inhibitors may have affected subphenotype attribution in the posthoc analysis, as clustering was based on data from the CounterCOVID trial, which was conducted before the introduction of tocilizumab.

The study’s findings have several implications. The neutral results of this study question the efficacy of targeting endothelial barrier disruption as a strategy in ARDS. Although preventing or attenuating endothelial barrier disruption may form a suitable strategy to prevent reaching the state of ARDS, the temporal dynamics of vascular leak in ARDS are still insufficiently understood. This precludes the current implementation of imatinib for the general COVID-19 ARDS population or translation to all-cause ARDS. A second implication is that a certain subpopulation may benefit from imatinib. Further clinical and biological characterization of subphenotypes is necessary here. Finally, the trial results underscore the importance of what is taking an increasingly central role in ARDS research: the characterization of biologically distinct subphenotypes for improved predictive enrichment in trials that examine potential treatments.

In conclusion, IV imatinib did not reduce EVLWi in invasively ventilated patients with COVID-19 ARDS. The administration of IV imatinib was well-tolerated and did not result in major safety concerns. Possible explanations for the lack of observed benefit include the biological heterogeneity of COVID-19 ARDS, concomitant use of immune-modulating medication and/or timing of imatinib administration. Further characterization of the imatinib-responsive subphenotype in future studies may help identify patients who benefit from vascular barrier-enhancing drugs.

## Supplementary Information

**Additional file 1.** Supplementray figures and tables.

**Additional file 2.** The study protocol.

**Additional file 3.** Statistical Analysis Plan.

## Data Availability

All anonymized patient data will be available after the publication of the article. The data can be requested from the corresponding author (j.aman@amsterdamumc.nl) by other researchers when reuse conditions are met.

## Abbreviations

ARDS: Acute respiratory distress syndrome
COVID-19: Coronavirus disease 2019
EVLW: Extravascular lung water
EVLWi: Extravascular lung water index
ICU: Intensive care unit
LUS: Lung ultrasound
PiCCO: Pulse contour cardiac output monitoring
VFD: Ventilator-free days
