# Supplementary material for: Efficacy and safety of intravenous imatinib in COVID-19 ARDS: a randomized, double-blind, placebo-controlled clinical trial
Source: Crit Care. 2023 Jun 8;27:226. doi: 10.1186/s13054-023-04516-4 (PMC10249575; doi:10.1186/s13054-023-04516-4)
Supplement: Supplementary file 1 — Additional file 1. Supplementray figures and tables. [file 13054_2023_4516_MOESM1_ESM.docx]

**Online Data Supplement 1**

**Title:** Efficacy and Safety of Intravenous Imatinib in COVID-19 ARDS: A Randomized, Double-Blind, Placebo-Controlled Clinical Trial

Table of Contents

[Complete list of in – and exclusion criteria 2](#_Toc134006240)

[9-point WHO ordinal scale for clinical improvement 4](#_Toc134006241)

[Table S1: Plasma biomarkers measured 5](#_Toc134006242)

[Table S2: Concomitant pharmacologic treatment for COVID-19 during Intensive Care Unit stay, stratified by treatment group 6](#_Toc134006243)

[Table S3: Reasons for premature discontinuation of study medication 7](#_Toc134006244)

[Table S4: Change in laboratory parameters and QTc time per treatment day 8](#_Toc134006245)

[Table S5: Change in plasma biomarker levels per treatment day 9](#_Toc134006246)

[Table S6: Baseline demographic and clinical patient characteristics, stratified per biological subphenotype 10](#_Toc134006247)

[Table S8: Baseline levels of plasma biomarkers, stratified per biological subphenotype 13](#_Toc134006248)

[Figure S1: Study recruitment and recruitment per centre 14](#_Toc134006249)

[Figure S2: Change in pulmonary vascular permeability index over time, stratified by treatment group 15](#_Toc134006250)

[Figure S3: 9-point WHO ordinal scale for clinical improvement on days 7, 10 and 28 16](#_Toc134006251)

[Figure S4: Dynamic changes of laboratory results and QTc times over time, stratified by randomisation group 17](#_Toc134006252)

[Figure S5: Dynamic changes of plasma biomarker levels over time, stratified by randomisation group 20](#_Toc134006253)

[Figure S6: Dynamic changes of ventilation parameters and SOFA score over time, stratified by treatment group 26](#_Toc134006254)

## Complete list of in – and exclusion criteria

**Inclusion criteria:**

- Age ≥18 years
- Moderate to severe ARDS, as defined by the Berlin definition for ARDS, intubated for invasive mechanical ventilation
- A PCR positive for SARS-CoV2 infection within the current disease episode
- Written informed consent provided by the patient or the patient’s legally authorized representative.

**Exclusion criteria:**

- Persistent septic shock (> 24 h) with a mean arterial pressure ≤ 65 mmHg and serum lactate level > 4 mmol/L (36 mg/dL) despite adequate volume resuscitation and vasopressor use (norepinephrine > 0.2 μg/kg/min) for > 6 h;
- Pre-existing chronic pulmonary disease, including known diagnosis of interstitial lung disease; known diagnosis of chronic obstructive pulmonary disease GOLD Stage IV or forced expiratory volume in 1 s < 30% predicted; diffusing capacity for carbon monoxide < 45% (if test results are available); total lung capacity < 60% of predicted (if test results are available);
- Chronic home oxygen treatment;
- Pre-existing heart failure with known left ventricular ejection fraction < 40%;
- Active treatment of haematological or non-haematological cancer with targeted immuno- or chemotherapy, or thoracic radiotherapy in the last year;
- Currently receiving extracorporeal life support;
- Severe chronic liver disease with Child-Pugh score > 12;
- Subjects in whom a decision to withdraw medical care is made (e.g., palliative setting);
- Inability of the ICU staff to initiate investigational medicinal product administration within 48 h of intubation;
- Known to be pregnant or breast-feeding;
- Enrolled in a concomitant clinical trial of an investigational medicinal product;
- White blood cell count < 2.5 × 10^9^/l; haemoglobin < 4.0 mmol/l or thrombocytes < 50 × 10^9^/l;
- The use of strong CYP3A4 inducers, including the following drugs: Carbamazepine, efavirenz, enzalutamide, phenobarbital, phenytoin, hypericum (St. John’s wort), mitotane, nevirapine, primidone, rifabutin, rifampicin;
- The presence of an intra-aortic balloon pump (IABP);
- Known medical history of aortic aneurysm in the trajectory of the PiCCO measurement between central venous line and arterial detector;
- Known medical history of an intracardiac shunt

## 9-point WHO ordinal scale for clinical improvement

| **Clinical status** | **Score** |
| --- | --- |
| Uninfected | 0 |
| Ambulatory, no limitation of activities | 1 |
| Ambulatory, limitation of activities | 2 |
| Hospitalised, no oxygen therapy | 3 |
| Hospitalised, oxygen by mask or nasal prongs | 4 |
| Hospitalised, non-invasive ventilation or high-flow nasal oxygen | 5 |
| Hospitalised, invasive mechanical ventilation | 6 |
| Hospitalised, invasive ventilation and additional organ support: vasopressors, renal replacement therapy or ECMO | 7 |
| Death | 8 |

*ECMO = extracorporeal membrane oxygenation; WHO = World Health Organization*

**SUPPLEMENTARY TABLES**

## Table S1: Plasma biomarkers measured

| **Endothelial** | **Coagulation** | **Inflammation** | **Cytokines** | **Epithelial** |
| --- | --- | --- | --- | --- |
| Angiopoietin-1 | Tissue Factor | Procalcitonin | IL-2 | SP-D |
| Angiopoietin-2 | D-dimer | TNFR1 | IL-6 | RAGE |
| E-selectin | vWF-A2 | Pentraxin 3 | IL-8 | Fas ligand |
| Syndecan-1 | Protein C | PDGF-AB | IL-10 |  |
| Syndecan-4 |  | Proteinase-3 | IL-17 |  |
| Thrombomodulin |  | Myeloperoxidase | IFN-gamma |  |
| ICAM-1 |  |  | TNF-alfa |  |
| Endocan |  |  |  |  |

*ICAM = intercellular adhesion molecule; IFN = interferon; IL = interleukin; PDGF-AB = platelet-derived growth factor, subunits A and B; RAGE = receptor for advanced glycation end-products; SP-D = surfactant protein-D; TNF = tumour necrosis factor; TNFR1 = tumour necrosis factor receptor 1; vWF-A2 = von Willebrand factor A2 domain. Biomarkers were measured using Luminex multiplex assay (R&D systems, Abington, UK) and Bioplex 200 (Bio-Rad, Hercules, California, USA) according to the manufacturer’s instructions.*

## Table S2: Concomitant pharmacologic treatment for COVID-19 during Intensive Care Unit stay, stratified by treatment group

|  | **Placebo (n = 33)** | **Imatinib (n = 33)** |
| --- | --- | --- |
| Monoclonal antibodies*, n (%) | 10 (30) | 5 (15) |
| Dexamethasone, n (%) | 33 (100) | 33 (100) |
| Interleukin-6 inhibitor^†^ | 31 (94) | 31 (94) |

**Casirivimab / imdevimab (2*.*4g single intravenous administration) or sotrovimab (500mg IV single intravenous administration); ^†^Tocilizumab (8mg/kg single intravenous administration) or sarilumab (400mg single intravenous administration).*

## Table S3: Reasons for premature discontinuation of study medication

| **Reason for discontinuation** | **Number of patients (%)** |
| --- | --- |
| Transfer to a non-participating hospital | 6 (9) |
| Discharge to ward | 15 (23) |
| Physician decision* | 2 (3) |
| Logistic/other^†^ | 11 (17) |

******One case of discontinuation due to elevated liver enzymes, one case due to ventricular tachycardia; ^†^e.g. central venous catheter removed due to line infection, problems with study medication order in an electronic patient file or with medication delivery from pharmacy, logistic clinical reasons.*

## Table S4: Change in laboratory parameters and QTc time per treatment day

| **Outcome** | **Change per treatment day** | **95% confidence interval** |
| --- | --- | --- |
| Alkaline phosphatase (U/L) | +2.57 | -1.59 – 6.74 |
| ALT (U/L) | +8.97 | -0.79 – 18.71 |
| AST (U/L) | +6.05 | -0.88 – 13.59 |
| gGT (U/L) | +9.82 | -7.83 – 27.56 |
| Bilirubin (µmol/L) | +0.26 | -0.23 – 0.76 |
| Creatinine (µmol/L) | -0.21 | -5.97 – 5.53 |
| eGFR (ml/min/1.73m^2^) | +0.09 | -0.50 – 0.76 |
| Haemoglobin (g/dL) | -0.03 | -0.20 – 0.10 |
| Thrombocytes x10^9^/L | -4.26 | -12.52 – 3.98 |
| Leucocytes x10^9^/L | +0.84 | 0.33 – 1.35 |
| NTproBNP (units/log10 ng/L) | +0.04 | -0.003 – 0.08 |
| QTc time (msec) | +1.28 | -0.50 – 3.08 |

*Table showing the results of the linear mixed effect models of the dynamic change of laboratory parameters and corrected QT time (QTc) per treatment day, with the placebo group as the reference group. ALT = alanine transaminase; AST = aspartate transaminase; eGFR = estimated glomerular filtration rate; gGT = gamma-glutamyl transferase; NTproBNP = N-terminal prohormone brain natriuretic peptide; ng = nanogram; msec = milliseconds; U/L = units per litre; µmol = micromol*

## Table S5: Change in plasma biomarker levels per treatment day

| **Biomarker** | **Change per treatment day** | **95% confidence interval** |
| --- | --- | --- |
| *Endothelial* |  |  |
| Angiopoietin-1 | -0.001 | -0.04 - 0.04 |
| Angiopoietin-2 | -0.02 | -0.06 - 0.01 |
| E-selectin | -0.01 | -0.04 - 0.02 |
| Syndecan-1 | +0.02 | 0.01 - 0.04 |
| Syndecan-4 | +0.002 | -0.04 - 0.04 |
| Thrombomodulin | +0.01 | -0.02 - 0.03 |
| ICAM-1 | -0.01 | -0.03 - 0.01 |
| Endocan | +0.07 | 0.04 - 0.09 |
| *Coagulation* |  |  |
| Tissue Factor | +0.02 | -0.001 - 0.05 |
| D-dimer | -0.03 | -0.06 - 0.01 |
| vWF-A2 | +0.02 | -0.03 - 0.06 |
| Protein C | -0.01 | -0.1 - 0.08 |
| *Inflammation* |  |  |
| Procalcitonin | -0.06 | -0.11 - (-0.01) |
| TNFR1 | +0.01 | -0.02 - 0.04 |
| Pentraxin-3 | +0.03 | -0.02 - 0.07 |
| PDGF-AB | +0.001 | -0.03 - 0.03 |
| Proteinase-3 | -0.05 | -0.21 - 0.12 |
| Myeloperoxidase | -0.01 | -0.12 - 0.10 |
| *Cytokines* |  |  |
| IL-2 | -0.01 | -0.04 - 0.03 |
| IL-6 | -0.03 | -0.11 - 0.05 |
| IL-8 | +0.01 | -0.03 - 0.06 |
| IL-10 | +0.04 | -0.01 - 0.09 |
| IL-17 | -0.02 | -0.08 - 0.04 |
| IFN-gamma | -0.003 | -0.04 - 0.03 |
| TNF-alfa | -0.02 | -0.06 - 0.01 |
| *Epithelial* |  |  |
| SP-D | +0.06 | 0.03 - 0.08 |
| RAGE | +0.03 | -0.01 - 0.07 |
| Fas ligand | +0.001 | -0.04 - 0.04 |

*Table showing the results of the linear mixed effect models of the change of plasma biomarkers concentration (in units/log10 pg/mL) per treatment day, with the placebo group as the reference group. All biomarker concentrations are presented as log10 transformed units. ICAM = intercellular adhesion molecule; IFN = interferon; IL = interleukin; PDGF-AB = platelet-derived growth factor, subunits A and B; RAGE = receptor for advanced glycation end-products; SP-D = surfactant protein-D; TNF = tumour necrosis factor; TNFR1 = tumour necrosis factor receptor 1; VCAM = vascular adhesion molecule; vWF-A2 = von Willebrand factor A2 domain.*

## Table S6: Baseline demographic and clinical patient characteristics, stratified per biological subphenotype

|  | **Subphenotype 1** | **Subphenotype 3** | **p-value** |
| --- | --- | --- | --- |
| n | 43 | 20 |  |
| **Admission characteristics** |  |  |  |
| Age in years, mean (SD) | 64 (9.5) | 59 (11.5) | 0.12 |
| Male sex, n (%) | 26 (61) | 10 (50) | 0.61 |
| BMI in kg/m^2^, median [IQR] | 29 [26, 33] | 30 [28, 34] | 0.28 |
| Days since COVID symptoms, mean (SD) | 12 (4.7) | 11 (4.3) | 0.60 |
| Days since positive SARS-CoV-2 PCR test, mean (SD) | 8 (4.3) | 8 (5.2) | 0.60 |
| ARDS classification (Berlin criteria) |  |  |  |
| Severe, n (%) | 9 (21) | 3 (15) | 0.83 |
| SOFA score, median [IQR] | 8 [7, 8] | 7 [7, 8] | 0.26 |
| Charlson comorbidity score, median [IQR] | 3 [2, 4] | 2 [1, 2] | 0.06 |
| **Comorbidities*** |  |  |  |
| COPD, n (%) | 1 (2) | 0 (0) | 1.00 |
| Heart failure, n (%) | 0 (0) | 0 (0) | NA |
| Renal failure, n (%) | 4 (9) | 0 (0) | 0.39 |
| Myocardial infarction, n (%) | 3 (7) | 0 (0) | 0.57 |
| **Ventilation and gas exchange** |  |  |  |
| TV/PBW, median [IQR] | 6.4 [5.9, 7.4] | 5.9 [5.6, 6.3] | 0.06 |
| PaO_2_/ FiO_2_ in mmHg, mean (SD) | 114 (31.3) | 119 (30.5) | 0.53 |
| PEEP in cmH_2_O, mean (SD) | 10.9 (2.7) | 10.9 (2.4) | 0.99 |
| **Laboratory measurements** |  |  |  |
| Haemoglobin in g/dL, median [IQR] | 13.2 [12.3, 13.9] | 13.1 [12.6, 13.9] | 0.74 |
| Leucocytes x10^9^/L, median [IQR] | 10.4 [7.8, 15.0] | 11.0 [9.0, 14.8] | 0.80 |
| Thrombocytes x10^9^/L, median [IQR] | 281.37 (91.22) | 339.20 (130.88) | 0.05 |
| D-dimer in mg/L, median [IQR] | 2.7 [1.1, 7.3] | 1.7 [0.9, 6.9] | 0.71 |
| Creatinine in µmol/L, median [IQR] | 99 [74, 120] | 70 [56, 88] | 0.01 |
| eGFR in mL/min/1.73m2, median [IQR] | 65 [45, 90] | 90 [75, 90] | 0.02 |
| NTproBNP in pg/mL, median [IQR] | 175 [73, 477] | 130 [58, 191] | 0.20 |
| AST in U/L, median [IQR] | 55 [42, 89] | 55 [39, 70] | 0.58 |
| ALT in U/L, median [IQR] | 43 [32, 106] | 61 [45, 105] | 0.26 |
| **PiCCO measurements** |  |  |  |
| EVLW in ml, median [IQR] | 1041 [918, 1338] | 964 [823, 1170] | 0.51 |
| EVLWi in ml/kg, median [IQR] | 15.5 [13.3, 18.4] | 14.5 [12.6, 19.7] | 0.61 |
| PVPI, median [IQR] | 3.5 [2.9, 4.2] | 3.1 [2.5, 3.8] | 0.18 |
| IL-6 receptor inhibitors^†^, n (%) | 40 (93) | 17 (85) | 0.58 |
| Dexamethasone, n (%) | 41 (95) | 17 (85) | 0.36 |
| Monoclonal antibodies^‡^, n (%) | 9 (21) | 4 (20) | 1.00 |

**Known history of the disease at the moment of randomisation; ^†^Tocilizumab (8mg/kg single intravenous administration) or sarilumab (400mg single intravenous administration) administered upon Intensive Care Unit admission; ^‡^Casirivimab / imdevimab (2.4g single intravenous administration) or sotrovimab (500mg IV single intravenous administration)*

*ARDS = Acute Respiratory Distress Syndrome; ALT = alanine transaminase; AST = aspartate transaminase; BMI = Body Mass Index; COPD = chronic obstructive pulmonary disease; COVID-19 = Coronavirus disease 2019; EVLW(i) = extravascular lung water (index); FiO_2_ = fraction of inspired oxygen; ICU = intensive care unit; IL-6 = interleukin-6; IQR = interquartile range; NTproBNP = N-terminal prohormone brain natriuretic peptide; PaO_2_ = partial pressure of oxygen; PCR = Polymerase chain reaction; PEEP = positive end-expiratory pressure; PiCCO = pulse contour cardiac output; PVPI = pulmonary vascular permeability index. QT_c_ = corrected QT interval time; SARS-CoV-2 = Severe acute respiratory syndrome coronavirus 2; SD = standard deviation; SOFA = Sequential Organ Failure Assessment; TV/PBW = tidal volume indexed to predicted body weight; PVPI = pulmonary vascular permeability index.*

**Table S7: Baseline demographic and clinical characteristics in subphenotype 3**

|  | Total | Placebo | Imatinib |
| --- | --- | --- | --- |
| n | 20 | 11 | 9 |
| **Admission characteristics** |  |  |  |
| Age in years, mean (SD) | 59.4 (11.5) | 56.4 (11.3) | 63.1 (11.3) |
| Male sex, n (%) | 10 (50) | 5 (46) | 5 (56) |
| BMI in kg/m^2^, median [IQR] | 29.8 [28.0, 34.4] | 30.5 [28.5, 38.2] | 28.7 [27.8, 30.1] |
| ARDS classification (Berlin criteria) |  |  |  |
| Severe, n (%) | 3 (15) | 1 (9) | 2 (22) |
| Moderate, n (%) | 17 (85) | 10 (91) | 7 (78) |
| SOFA score, median [IQR] | 7.0 [7.0, 7.5] | 7.0 [7.0, 7.3] | 7.0 [6.0, 9.0] |
| Charlson comorbidity score, median [IQR] | 2.0 [1.0, 2.3] | 2.0 [0.5, 2.0] | 2.0 [2.0, 3.0] |
| Fluid balance in litres, mean (SD) | 0.2 (0.9) | 0.0 (0.4) | 0.5 (1.2) |
| QTc in msec, mean (SD) | 444.12 (35.01) | 438.09 (31.16) | 455.17 (41.86) |
| IL-6 receptor inhibitors^*^, n (%) | 17 (85) | 9 (82) | 8 (89) |
| Dexamethasone, n (%) | 17 (85) | 10 (91) | 7 (78) |
| Completed 10-day dexamethasone treatment before ICU admission, n (%) | 3 (15) | 1 (9) | 2 (12) |
| Days from symptoms to intubation, mean (SD) | 11.0 (4.3) | 10.6 (4.5) | 11.5 (4.4) |
| Vaccinated, n (%) | 10 (50) | 6 (55) | 4 (44) |
| **Comorbidities** |  |  |  |
| COPD, n (%) | 0 (0) | 0 (0) | 0 (0) |
| Heart failure, n (%) | 0 (0) | 0 (0) | 0 (0) |
| Renal failure, n (%) | 0 (0) | 0 (0) | 0 (0) |
| Myocardial infarction, n (%) | 0 (0) | 0 (0) | 0 (0) |
| **Ventilation and gas exchange** |  |  |  |
| TV/PBW in ml/kg, median [IQR] | 5.9 [5.8, 6.3] | 6.0 [5.8, 6.5] | 5.8 [5.4, 6.1] |
| PaO_2_/FiO_2_ in mmHg, mean (SD) | 119.1 (30.5) | 117.7 (29.8) | 120.7 (33.0) |
| PEEP in cm H_2_O, mean (SD) | 10.94 (2.4) | 11.71 (2.0) | 9.99 (2.5) |
| **Laboratory measurements** |  |  |  |
| Haemoglobin in g/dL, mean (SD) | 8.1 (0.9) | 8.2 (0.8) | 8.1 (1.0) |
| Leucocytes x 10^9^/L, median [IQR] | 11.0 [9.0, 14.8] | 11.0 [8.1, 13.4] | 10.9 [9.7, 16.2] |
| Thrombocytes x 10^9^/L, mean (SD) | 339.2 (130.9) | 338.6 (123.9) | 339.9 (146.6) |
| D-dimer in mg/L, median [IQR] | 1.7 [0.9, 6.9] | 1.6 [0.9, 2.6] | 4.8 [1.6, 17.9] |
| Creatinine in micromol/L, median [IQR] | 69.5 [55.8, 88.0] | 72.0 [55.5, 88.0] | 67.0 [63.0, 75.0] |
| NTproBNP in pg/ml, median [IQR] | 130.0 [57.5, 190.5] | 130.0 [74.5, 321.5] | 107.5 [57.0, 167.3] |
| AST in U/L, median [IQR] | 55.0 [39.3, 70.0] | 57.5 [42.8, 78.8] | 48.0 [36.8, 59.5] |
| ALT in U/L, median [IQR] | 61.0 [45.0, 104.5] | 67.5 [53.5, 115.0] | 56.0 [38.0, 62.0] |
| **PiCCO measurements** |  |  |  |
| EVLW in ml, median [IQR] | 964 [823, 1170] | 964 [823, 1160] | 1004 [843, 1268] |
| ELWi in ml/kg, median [IQR] | 14.5 [12.6, 19.7] | 13.4 [12.6, 17.3] | 15.3 [12.6, 20.7] |
| PVPI, median [IQR] | 3.1 [2.5, 3.8] | 3.1 [2.3, 3.4] | 3.3 [2.6, 5.1] |

## Table S8: Baseline levels of plasma biomarkers, stratified per biological subphenotype

|  | **Subphenotype 1**  **(n = 43)** | **Subphenotype 3**  **(n = 20)** | **p-value** |
| --- | --- | --- | --- |
| Tissue factor in pg/mL, median [IQR] | 47.2 [32.4, 72.5] | 37.1 [29.0, 49.9] | 0.14 |
| D-dimer in pg/mL, median [IQR] | 3496450 [2332397, 4747050] | 3566818 [1843670, 4553787] | 0.62 |
| E-selectin in pg/mL, median [IQR] | 16403 12759, 21635] | 19056 [16125, 23096] | 0.24 |
| ICAM-1 in pg/mL, median [IQR] | 218001 [167794, 342029] | 201084 [130714, 253558] | 0.16 |
| IFN-gamma in pg/mL, median [IQR] | 18.6 [13.8, 23.8] | 12.2 [10.8, 14.3] | 0.001 |
| IL-2 in pg/mL, median [IQR] | 11.0 [9.2, 14.7] | 8.6 [6.4, 9.6] | 0.004 |
| IL-6 in pg/mL, median [IQR] | 269.5 [111.9, 489.8] | 54.9 [28.9, 101.9] | <0.001 |
| IL-8 in pg/mL, median [IQR] | 30.9 [14.6, 45.0] | 21.6 [14.6, 27.2] | 0.23 |
| PDGF-AB in pg/mL, median [IQR] | 692 [421, 880] | 611 [309, 1154] | 0.94 |
| Pentraxin-3 in pg/mL, median [IQR] | 5491 [3165, 8709] | 4501 [2440, 6641] | 0.24 |
| sRAGE in pg/mL, median [IQR] | 6145 [2981, 11361] | 4422 [1543, 7255] | 0.051 |
| SP-D in pg/mL, median [IQR] | 13000 [8873, 21287] | 18871 [15518, 26377] | 0.022 |
| Syndecan-1 in pg/mL, median [IQR] | 4994 [3951, 5830] | 3675 [3059, 4502] | 0.012 |
| Syndecan-4 in pg/mL, median [IQR] | 467 [311, 555] | 499 [403, 672] | 0.26 |
| Thrombomodulin in pg/mL, median [IQR] | 7099 [5011, 10506] | 5567 [4689, 7714] | 0.10 |
| TNF-alpha in pg/mL, median [IQR] | 11.4 [8.4, 16.6] | 7.8 [6.5, 9.5] | 0.003 |
| TNFR1 in pg/mL, median [IQR] | 2130 [1524, 2836] | 1382 [1085, 1893] | 0.004 |
| vWF in pg/mL, median [IQR] | 7978 [5356, 10782] | 8049 [6298, 9068] | 0.47 |
| Ang-2 / Ang-1 in pg/mL, median [IQR] | 0.28 [0.16, 0.52] | 0.22 [0.13, 0.58] | 0.61 |

*Ang = angiopoietin; ICAM = intercellular adhesion molecule; IFN = interferon; IL = interleukin; IQR = Interquartile range; PDGF-AB = platelet-derived growth factor, subunits A and B; pg = pictogram; RAGE = receptor for advanced glycation end-products; SP-D = surfactant protein-D; TNF = tumour necrosis factor; TNFR1 = tumour necrosis factor receptor 1; VCAM = vascular adhesion molecule; vWF-A2 = von Willebrand factor A2 domain.*

**SUPPLEMENTARY FIGURES**

## Figure S1: Study recruitment and recruitment per centre


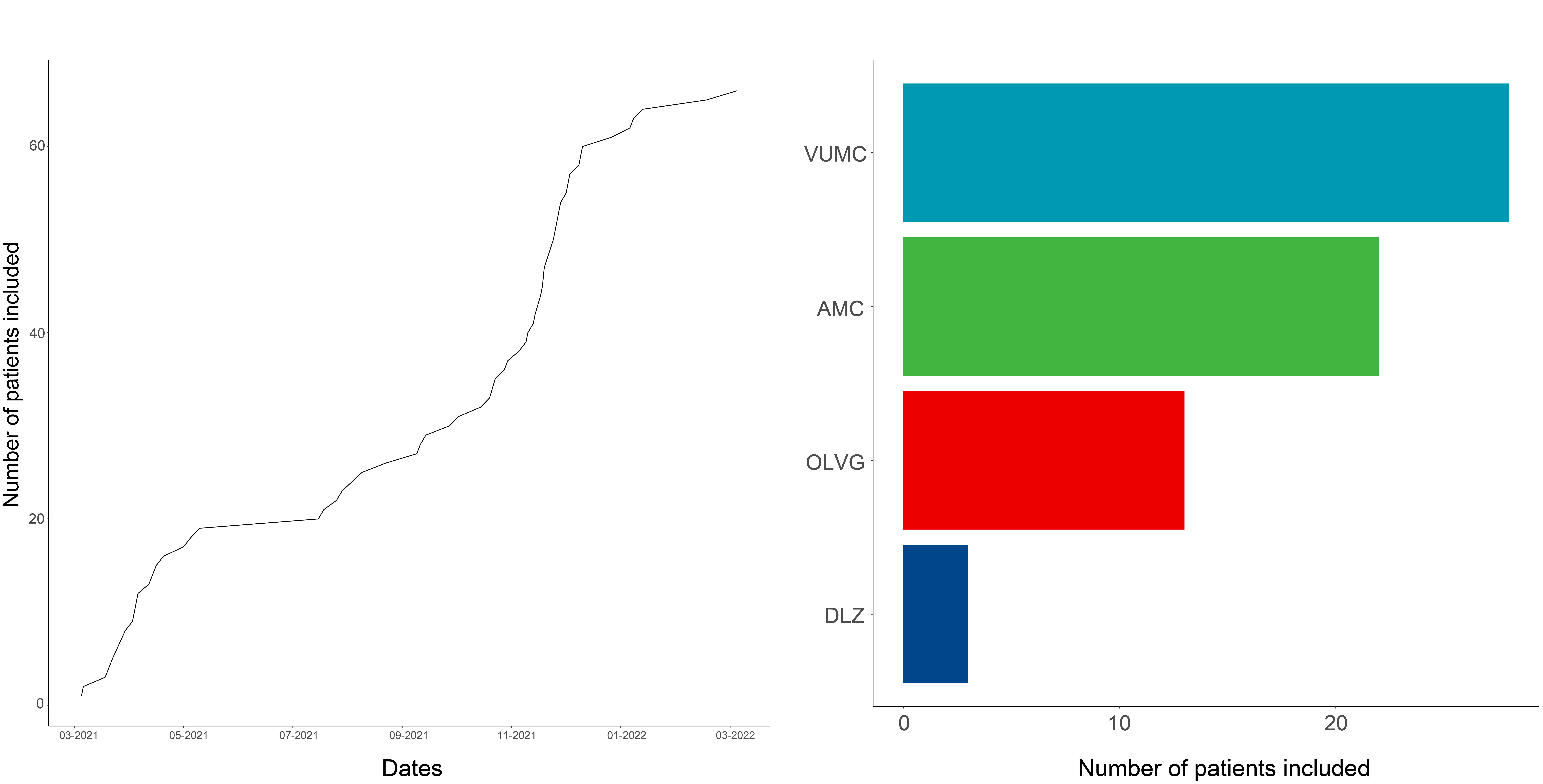


*Inclusion rate between March 2021 and March 2022 (panel A) and the number of inclusions per participating centre (panel B. AMC = Amsterdam University Medical Centres (AUMC), location Amsterdam Medical Centre; DLZ = Dijklander hospital; OLVG = Onze Lieve Vrouwe Gasthuis Hospital, location Oost; VUMC = AUMC, location Vrije Universiteit Medical Centre.*

## Figure S2: Change in pulmonary vascular permeability index over time, stratified by treatment group

**
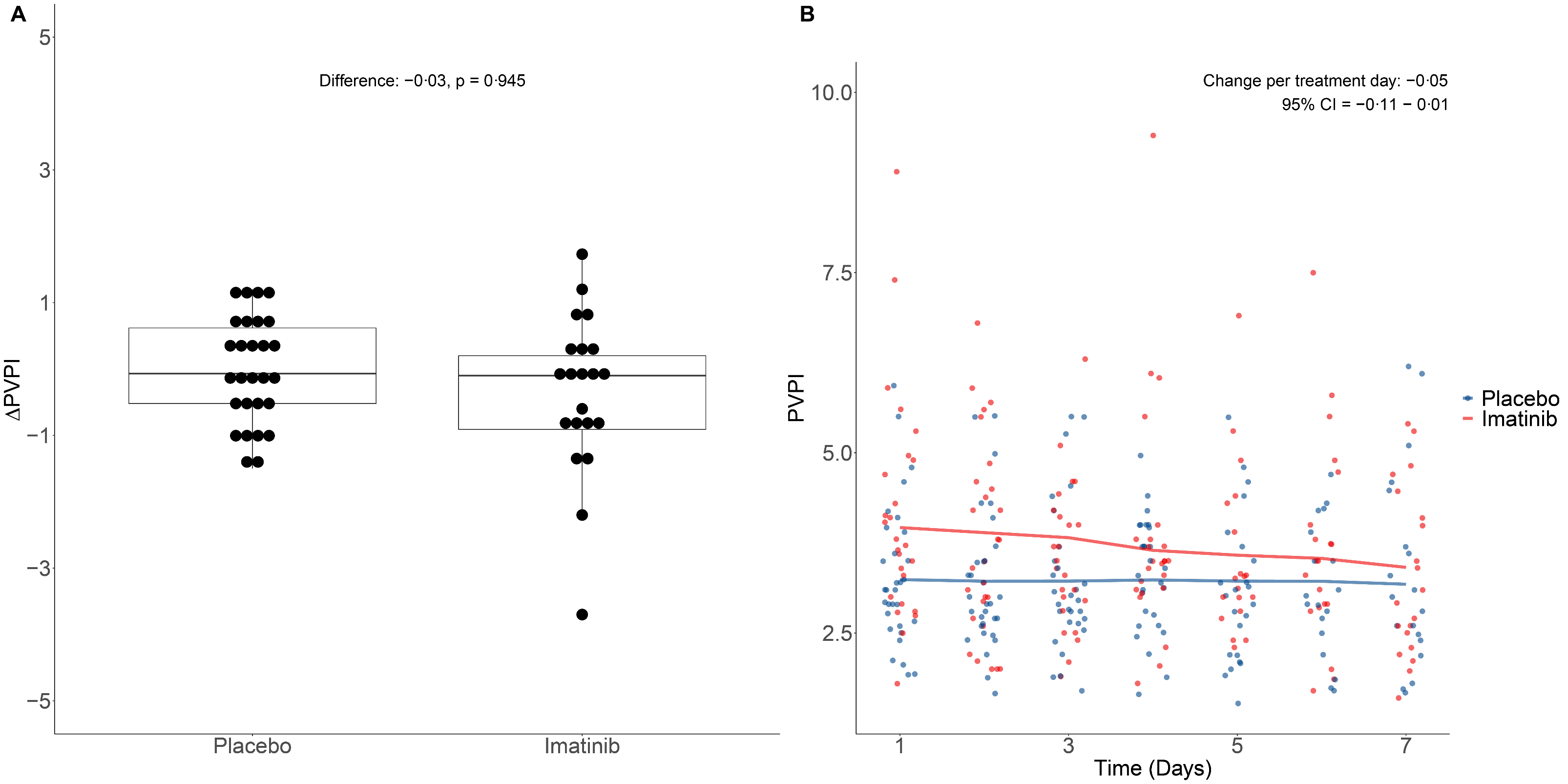
**

*Boxplot (panel A) depicting the distribution of the change in pulmonary vascular permeability index (∆PVPi) in the imatinib group versus placebo between day 1 and day 4. Scatterplot (panel B) depicting the dynamic changes of PVPi in the imatinib group versus the placebo group from day 1 to day 7.*

## Figure S3: 9-point WHO ordinal scale for clinical improvement on days 7, 10 and 28

*****The 9-point WHO ordinal scale for clinical improvement shows the proportion of patients by clinical status on day 7, day 10 and day 28 stratified by treatment group. The proportions of patients who were Hospitalised and required oxygen treatment in the imatinib versus the placebo group were 97% versus 97% on day 7, 93% versus 91% on day 10 and 58% versus 40% on day 28, respectively.*

## Figure S4: Dynamic changes of laboratory results and QTc times over time, stratified by randomisation group


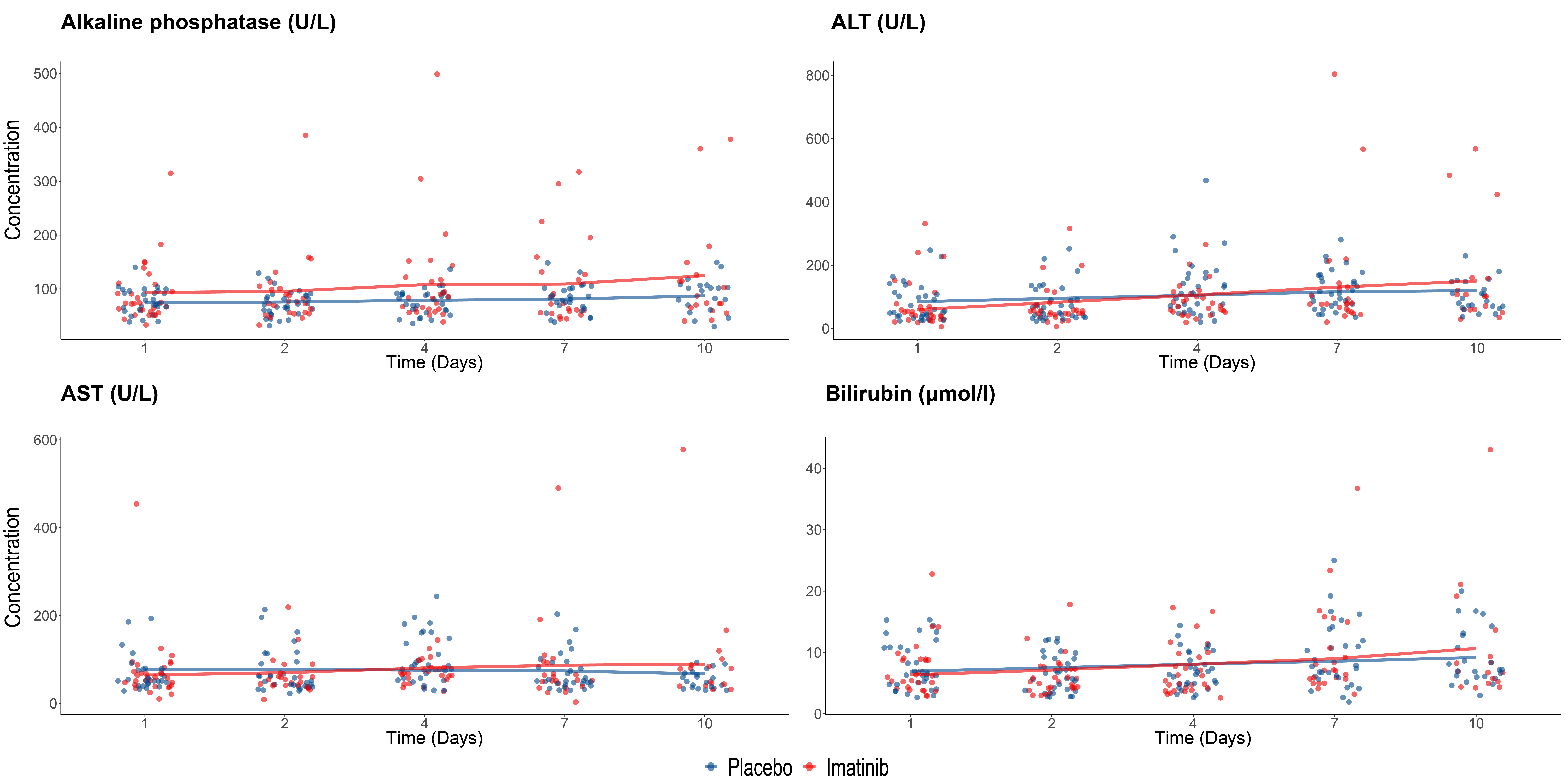

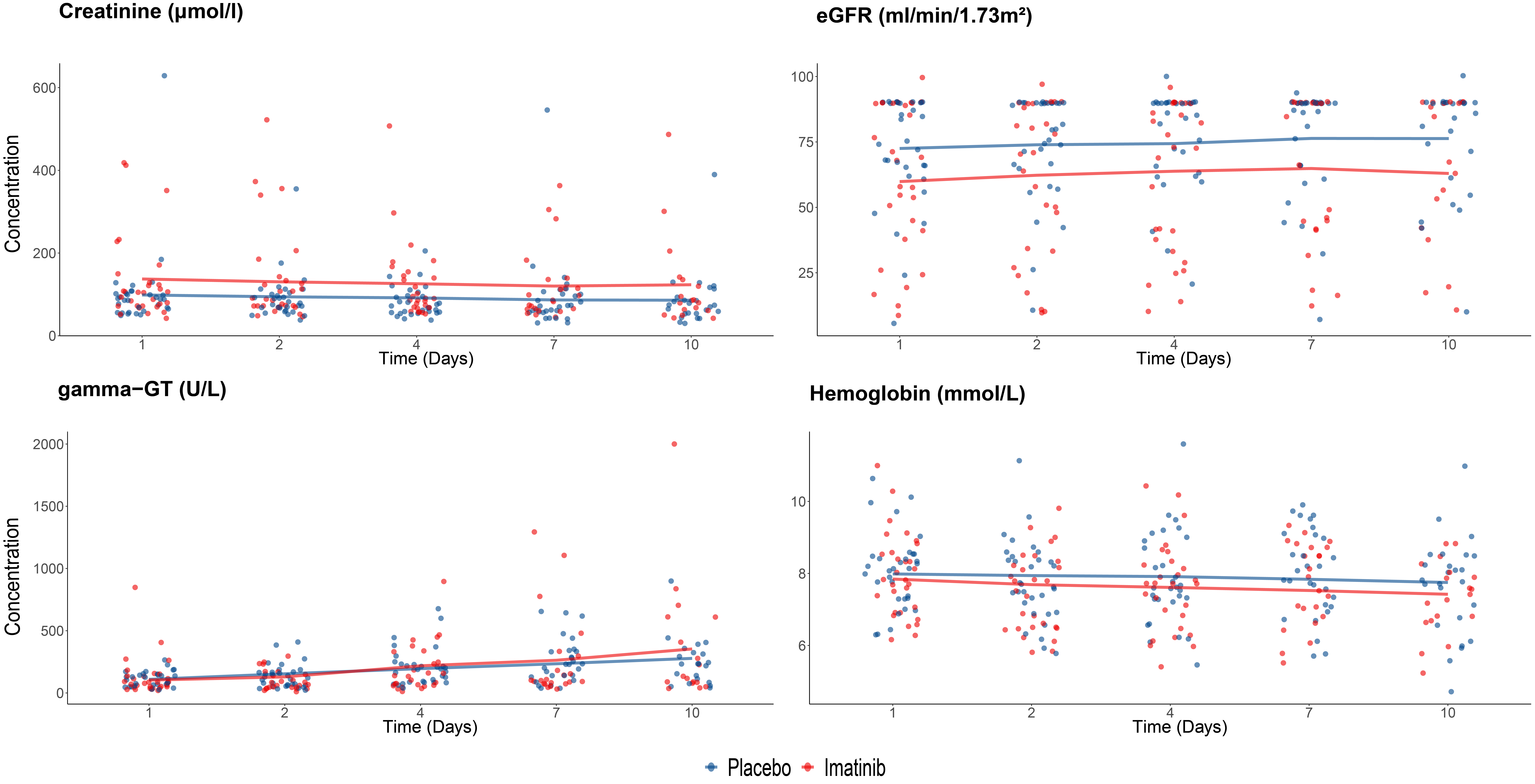

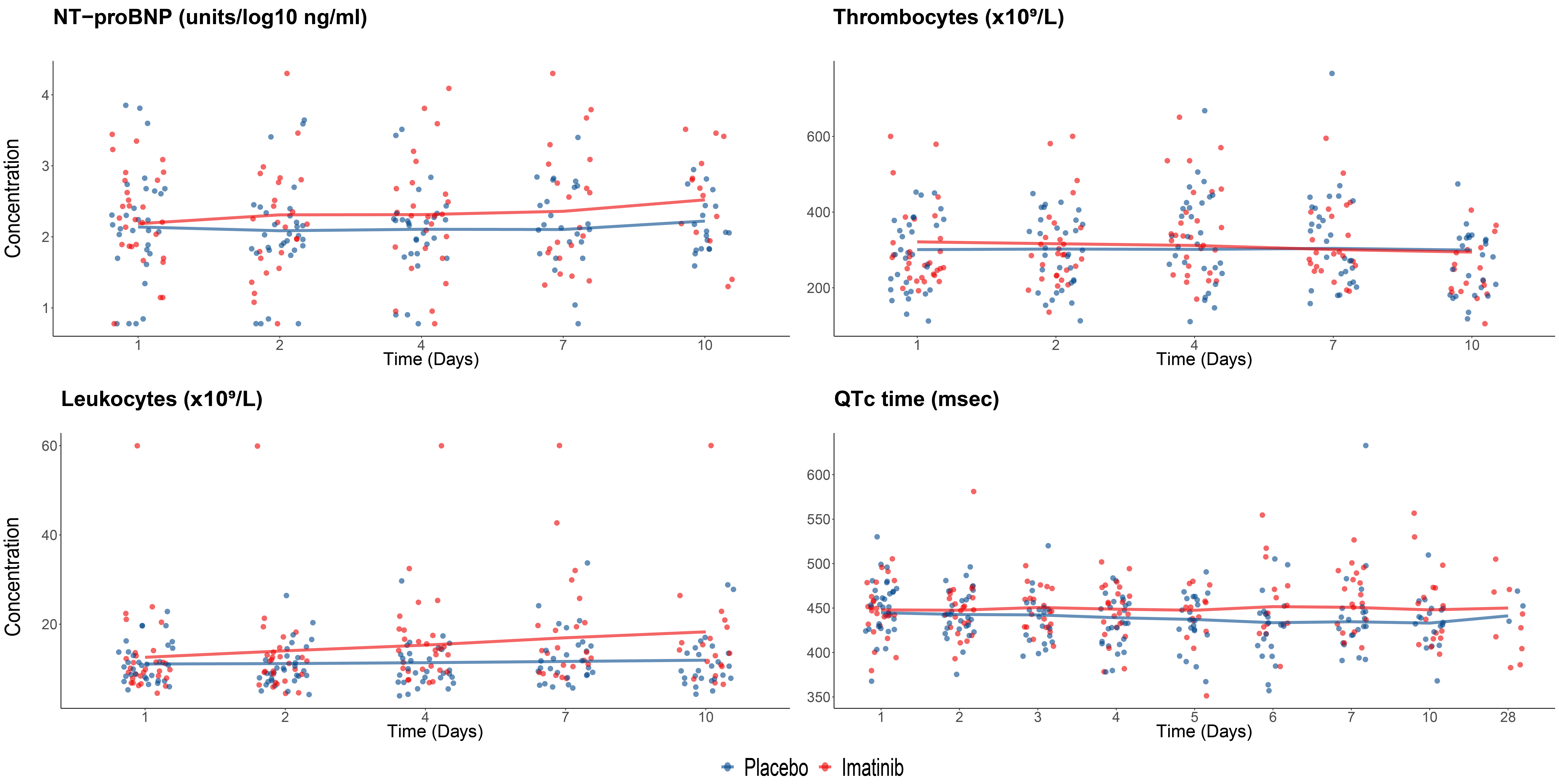


*Figure panel depicting laboratory results and corrected QT (QTc) time collected during the study period, stratified by treatment. ALT = alanine transaminase; AST = aspartate transaminase; eGFR = estimated glomerular filtration rate; gamma-GT = gamma-glytamyl transferase; NTproBNP = N-terminal prohormone brain natriuretic peptide; QTc = corrected QT time.*

## Figure S5: Dynamic changes of plasma biomarker levels over time, stratified by randomisation group

**A – Inflammation**





**B – Epithelial**


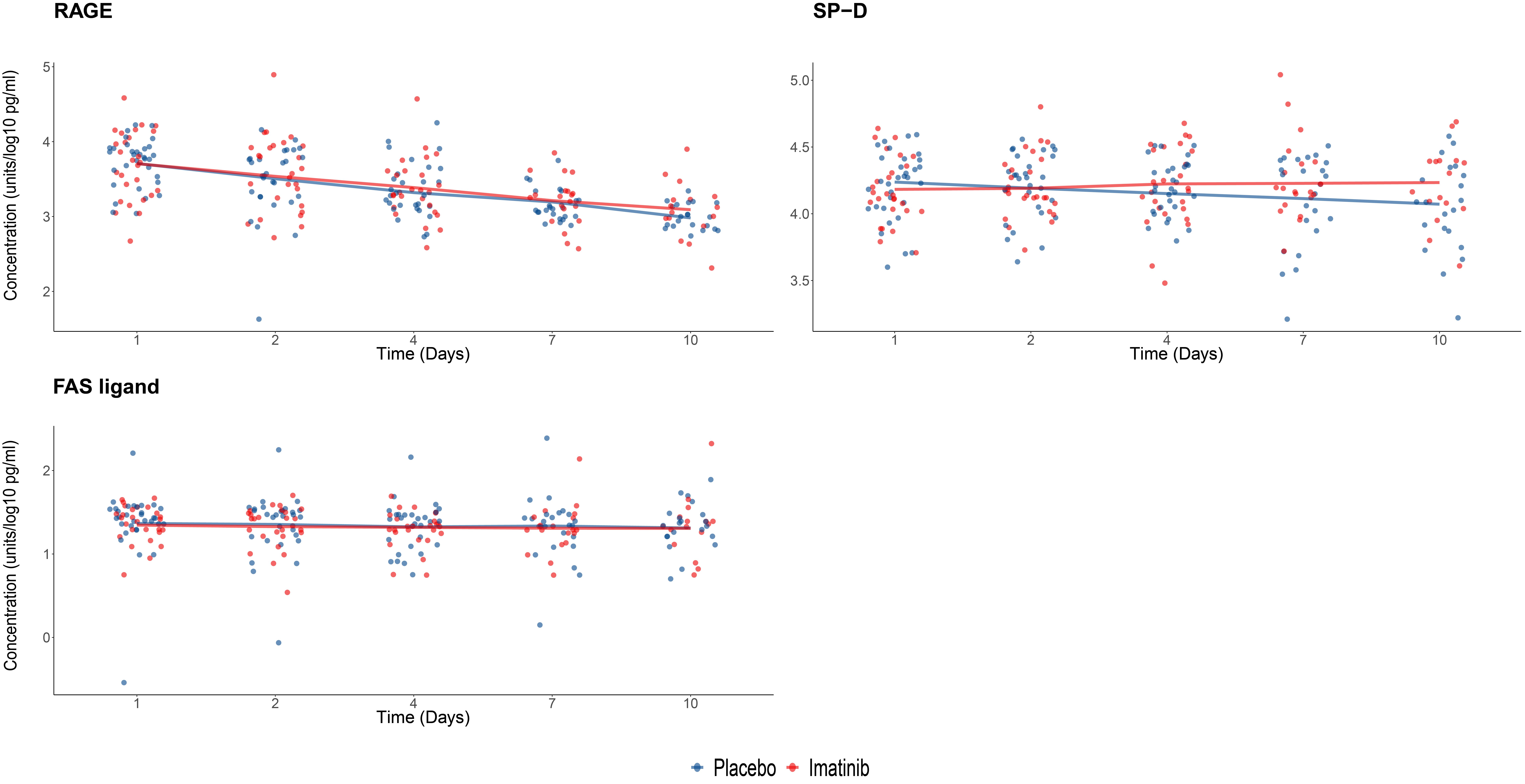


**C – Endothelial**


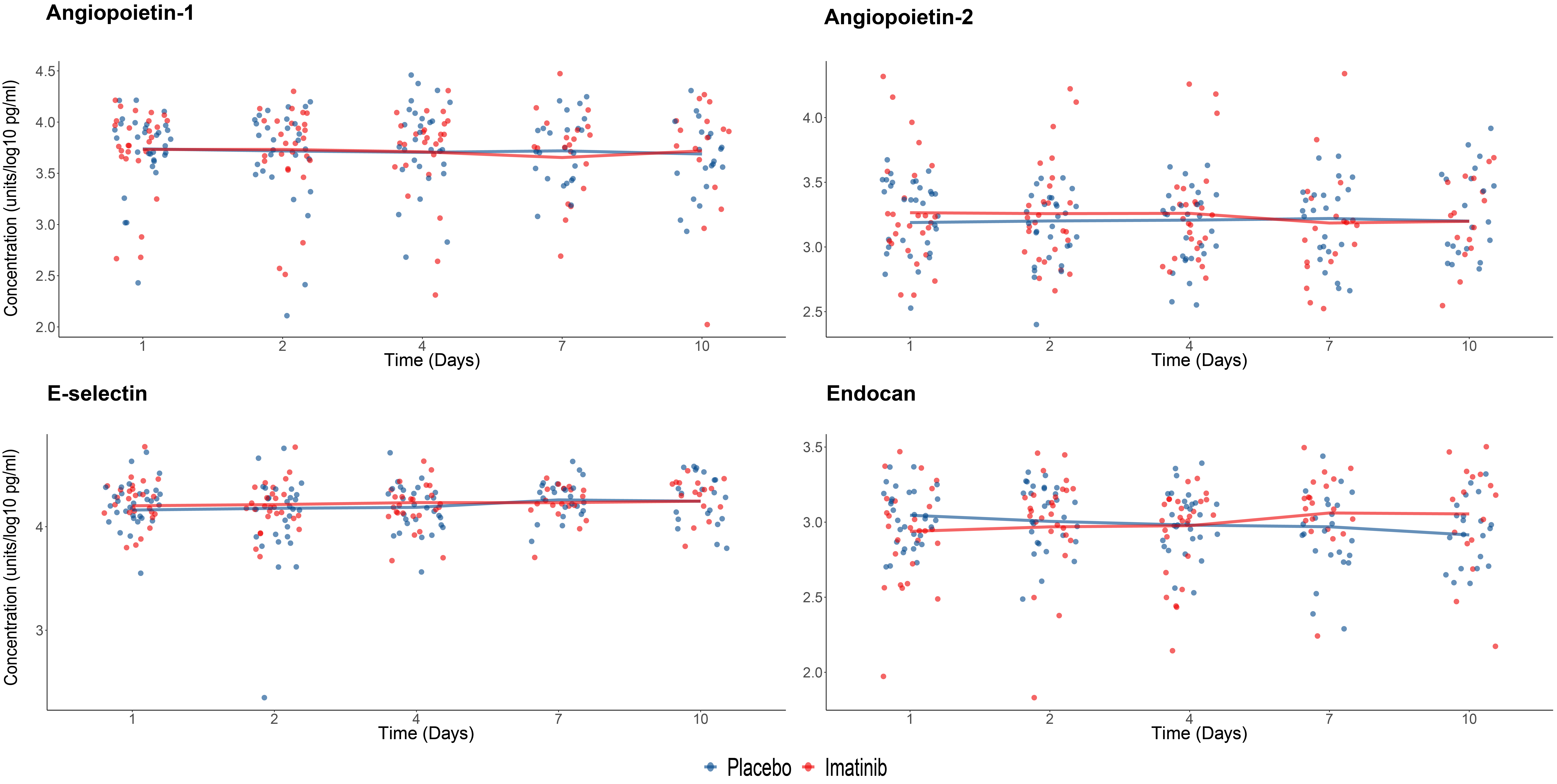

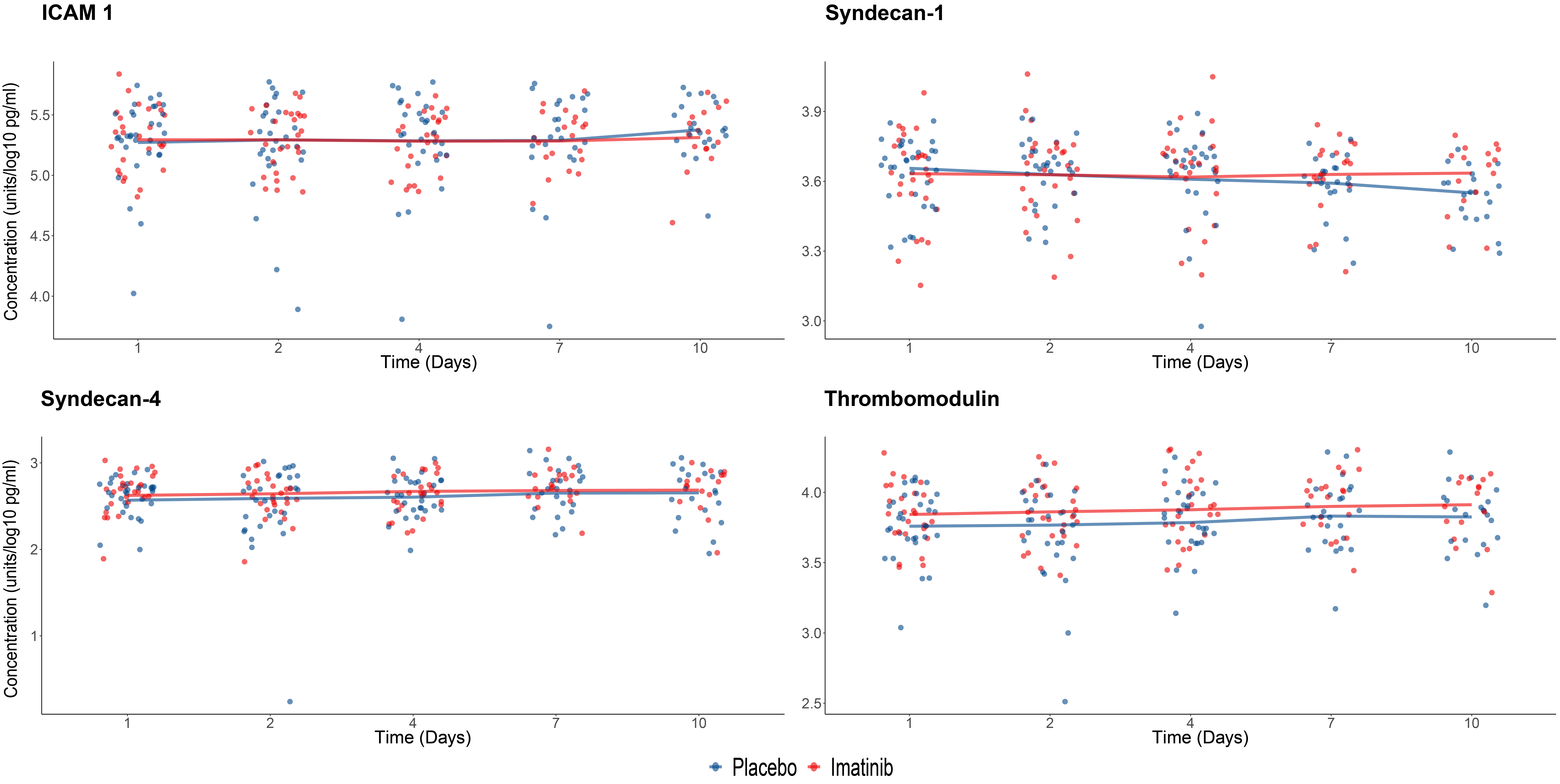


**D – Cytokines**


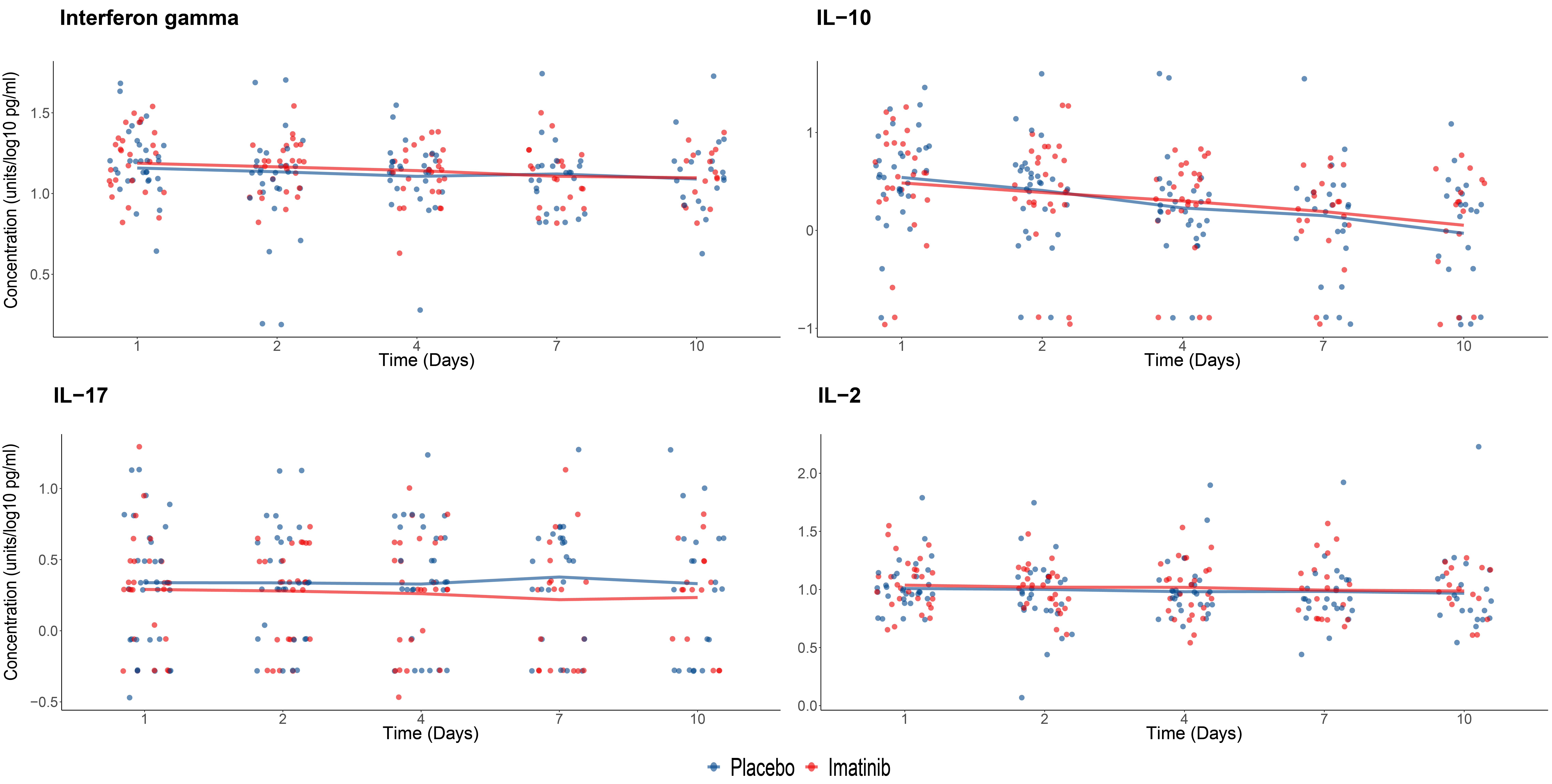


**E – Coagulation**


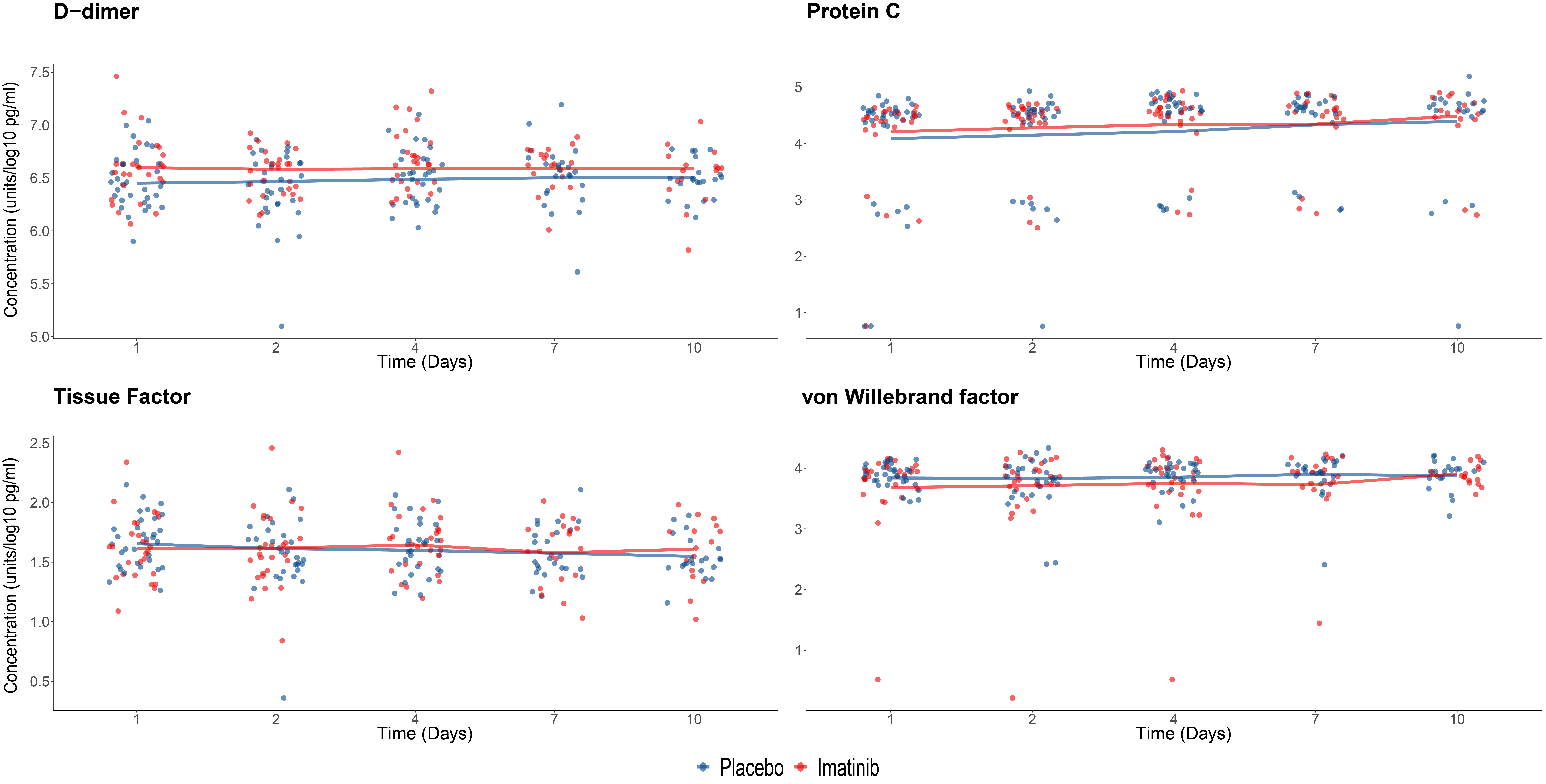


*Figure panel depicting dynamic changes of plasma biomarker concentrations over the study period, stratified by imatinib versus placebo and grouped by biomarkers representative of (A) inflammation, (B) epithelial injury, (C) endothelial dysfunction, (D) inflammatory cytokines and (E) coagulation. Y-axis is shown on a log10 scale. ICAM = intercellular adhesion molecule; IFN = interferon; IL = interleukin; PDGF-AB = platelet-derived growth factor, subunits A and B; RAGE = receptor for advanced glycation end-products; SP-D = surfactant protein-D; TNF = tumour necrosis factor; TNFR1 = tumour necrosis factor receptor 1; VCAM = vascular adhesion molecule; vWF-A2 = von Willebrand factor A2 domain.*

## Figure S6: Dynamic changes of ventilation parameters and SOFA score over time, stratified by treatment group


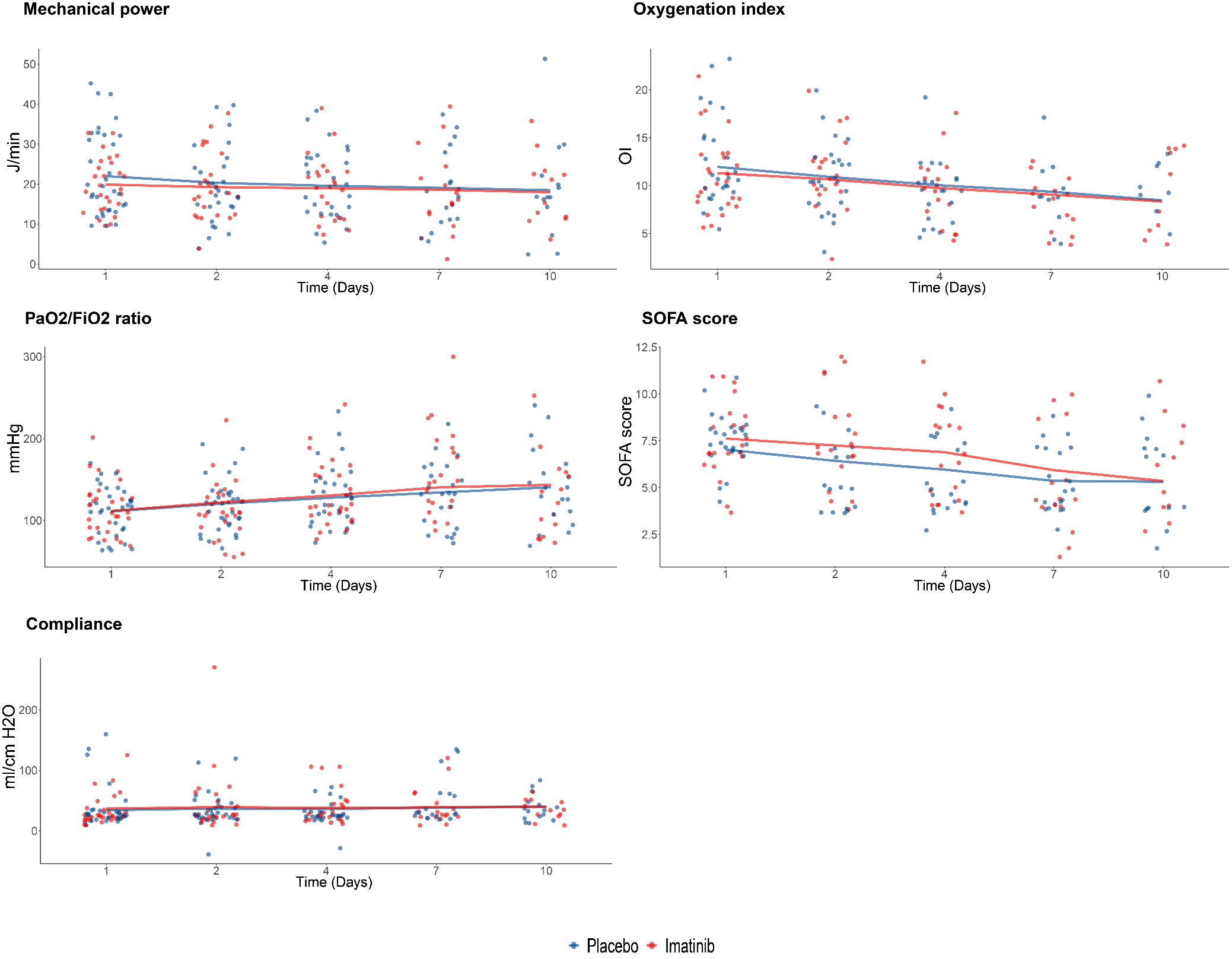


*Figure panel depicting dynamic changes of ventilation parameters over the study period, stratified by imatinib versus placebo. FiO_2_ = fraction of inspired oxygen; PaO_2_ = partial pressure of oxygen; SOFA = Sequential Organ Failure Assessment.*
