# Supplementary material for: Efficacy and safety of intravenous imatinib in COVID-19 ARDS: a randomized, double-blind, placebo-controlled clinical trial
Source: Crit Care. 2023 Jun 8;27:226. doi: 10.1186/s13054-023-04516-4 (PMC10249575; doi:10.1186/s13054-023-04516-4)
Supplement: Supplementary file 2 — Additional file 2. The study protocol. [file 13054_2023_4516_MOESM2_ESM.pdf]

RESEARCH PROTOCOL IMPENTRI STUDY

TITLE '*A randomised, double-blind, placebo-controlled study to investigate the safety and efficacy of intravenous imatinib mesylate (Impentri®) in subjects with Acute Respiratory Distress Syndrome induced by COVID-19*'

**Protocol ID**	INVENT COVID
**Short title**	Intravenous imatiNib in mechanically VENTilated COVID-19 patients
**EudraCT number**	2020-005447-23
**Version**	4.1
**Date**	24 February 2022
**Coordinating investigator/project leader:**	Dr. J. Aman, Dept. of Pulmonary Diseases<br>Secretary Dept. Pulmonary Disease | ZH 4F 04 | De Boelelaan 1117, 1081 HV Amsterdam.<br>Post address: Postbus 7057, 1007 MB Amsterdam<br>T: 020-4441896 | E: j.aman@amsterdamumc.nl
**Principal investigator(s)**	
Location AMC:	Dr. L.D.J. Bos<br>Dept. of Pulmonary Diseases & Dept. of Intensive Care, Amsterdam UMC, Amsterdam, The Netherlands
Location VUMC	Dr. P.R. Tuinman<br>Dept. of Intensive Care, Amsterdam UMC, location VUMC, Amsterdam, The Netherlands
Location OLVG Oost	Dr. N. Juffermans<br>Dept. of Intensive Care, OLVG Oost & Amsterdam UMC, Amsterdam, The Netherlands
**Sponsor**	Amsterdam UMC, location VUMC
**Subsidising party**	European Union

**Independent expert (s)**	Dr. J. Horn, Dept. of Intensive Care<br>Amsterdam UMC, location AMC<br>Amsterdam, The Netherlands
**Laboratory sites**	Amsterdam UMC, location VUMC<br><br>Amsterdam UMC, location AMC<br><br>Onze Lieve Vrouwe Gasthuis, location Oost
**Pharmacy**	I.H. Bartelink, AUMC location VUMC<br>P. Bet, AUMC location VUMC<br>N. Bouwhuis, AUMC location AMC
**Steering Committee Members**	Dr. J. Aman, Dept. of Pulmonary Diseases<br>Amsterdam UMC, Amsterdam, The Netherlands<br><br>Prof. Dr. H.J. Bogaard, Dept. of Pulmonary Diseases<br>Amsterdam UMC, Amsterdam, The Netherlands<br><br>Dr. L.D.J. Bos, Dept. of Pulmonary Diseases & Dept. of Intensive Care<br>Amsterdam UMC, Amsterdam, The Netherlands<br><br>Prof. dr. L. Heunks, Dept. of Intensive Care<br>Amsterdam UMC, location VUMC, Amsterdam, The Netherlands<br><br>Dr. N. Juffermans, Dept. of Intensive Care<br>OLVG Oost & Amsterdam UMC, Amsterdam, The Netherlands<br><br>Prof.dr. M.J. Schultz, Dept. of Intensive Care<br>Amsterdam UMC, location AMC, Amsterdam, The Netherlands<br><br>Prof. dr. P.R. Tuinman, Dept. of Intensive Care<br>Amsterdam UMC, location VUMC, Amsterdam, The Netherlands
**Pharmacovigilance, Medical Monitoring and Data Management:**	Simbec-Orion. Merthyr Tydfil Industrial Park, Merthyr Tydfil, CF48 4DR, United Kingdom
**IMP Supply:**	Exvastat Ltd, 5 Tenison Ave, Cambridge, CB1 2DX UK.

PROTOCOL SIGNATURE SHEET

Name	Signature	Date
**Head of Department:**<br><br>Prof. dr. M. Vroom<br>Head of Intensive Care<br>Amsterdam UMC, location AMC	DocuSigned by:<br>Prof.dr.M. Vroom<br>Naam ondertekenaar: Prof.dr.M. Vroom<br>Reden voor ondertekening: Ik keur dit document goed<br>Ondertekentijd: 01-apr-2022 | 11:22 BST<br>8AC9D8C3FEEC4F489C3E0D327457419A	
Prof. dr. A. Girbes<br>Head of Intensive Care<br>Amsterdam UMC, location AMC	DocuSigned by:<br>Pieter Roel Tuinman<br>Naam ondertekenaar: Pieter Roel Tuinman<br>Reden voor ondertekening: Ik keur dit document goed<br>Ondertekentijd: 23-apr-2022 | 08:58 BST<br>139557A0944B4C47B2021F0CFB01799E	On behalf of Prof Girbes
Prof.dr. A. Vonk Noordegraaf<br>Head-of-Department of Pulmonary Diseases, Amsterdam UMC	DocuSigned by:<br>Prof.dr.A. Vonk Noordegraaf<br>Naam ondertekenaar: Prof.dr.A. Vonk Noordegraaf<br>Reden voor ondertekening: Ik keur dit document goed<br>Ondertekentijd: 30-mrt-2022 | 12:48 CEST<br>3A0CA5AAC6314B2AA99B9343A04DBC7E	
**Coordinating Investigator/Project leader:**<br><br>J. Aman, MD PhD<br>Dept. of Pulmonary Diseases, Amsterdam UMC	DocuSigned by:<br>Jurjan Aman<br>Naam ondertekenaar: Jurjan Aman<br>Reden voor ondertekening: Ik keur dit document goed<br>Ondertekentijd: 30-mrt-2022 | 11:40 BST<br>3BE720AB11614B42B0789CDBD0B82B36	

TABLE OF CONTENTS

SUMMARY 8
1. INTRODUCTION AND RATIONALE 9
1.1 Background of ARDS due to COVID-19 9
1.2 Rationale for intervention 10
2. OBJECTIVES 13
3. STUDY DESIGN 13
4. STUDY POPULATION 13
4.1 Population (base) 13
4.2 Inclusion criteria 13
4.3 Exclusion criteria 14
4.4 Sample size calculation 15
5. TREATMENT OF SUBJECTS 15
5.1 Investigational product/treatment 15
5.2 Use of co-intervention (if applicable) 15
5.3 Escape medication (if applicable) 15
5.4 Interactions 15
6. INVESTIGATIONAL PRODUCT 16
6.1 Name and description of investigational product(s) 16
6.2 Dosage and Administration 16
6.3 Summary of findings from non-clinical studies 16
6.4 Summary of findings from clinical studies 17
6.5 Summary of known and potential risks and benefits 18
6.6 Description and justification of route of administration and dosage 18
6.7 Dose modifications 18
6.8 Preparation and labeling of Investigational Medicinal Product 18
6.9 Treatment compliance 19
6.10 Drug accountability 19
7. NON-INVESTIGATIONAL PRODUCT 19
8. METHODS 19
8.1 Study parameters/endpoints 19
8.1.1 Main study parameter/endpoint 19
8.1.2 Secondary study parameters/endpoints (if applicable) 20
8.1.3 Other study parameters (if applicable) 21
8.2 Randomisation, blinding and treatment allocation 21
8.3 Study procedures 21
8.4 Concomitant Medication & Supportive Care 23
8.4.1 Concomitant Medication 23
8.4.2 Concomitant Supportive Care 23
8.5 Withdrawal of individual subjects 24
8.6 Replacement of individual subjects after withdrawal 25
8.7 Follow-up of subjects withdrawn from treatment 25

8.8 Premature termination of the study ... 26
9. SAFETY REPORTING ... 26
9.1 Temporary halt for reasons of subject safety ... 26
9.2 AEs, SAEs and SUSARs ... 26
9.2.1 Definitions ... 26
9.2.2 Reporting of Adverse Events (AE) and Serious Adverse Events (SAE) ... 27
9.2.3 Reporting of Suspected unexpected serious adverse reactions (SUSARs) ... 28
9.2.4 Reporting to Competent Authority and Medical Ethics Committee ... 28
9.3 Annual safety report ... 29
9.4 Data Safety Monitoring Board (DSMB) / Safety Committee ... 29
10. STATISTICAL ANALYSIS ... 29
10.1 Primary study parameter(s) ... 30
10.2 Secondary study parameter(s) ... 30
10.3 Other study parameters ... 31
10.4 Interim analysis (if applicable) ... 32
11. ETHICAL CONSIDERATIONS ... 32
11.1 Regulation statement ... 32
11.2 Recruitment and consent ... 32
11.2.1 Recruitment ... 32
11.2.2 Deferred consent ... 32
11.2.3 No consent in subjects who die before obtaining deferred consent ... 34
11.2.4 Conclusion deferred consent ... 34
11.2.5 Consent procedure ... 34
11.3 Objection by minors or incapacitated subjects (if applicable) ... 35
11.4 Benefits and risks assessment, group relatedness ... 35
11.5 Compensation for injury ... 35
11.6 Incentives (if applicable) ... 35
12. ADMINISTRATIVE ASPECTS, MONITORING AND PUBLICATION ... 36
12.1 Handling and storage of data and documents ... 36
12.2 Monitoring and Quality Assurance ... 36
12.3 Amendments ... 37
12.4 Annual progress report ... 37
12.5 Temporary halt and (prematurely) end of study report ... 37
12.6 Public disclosure and publication policy ... 37
13. STRUCTURED RISK ANALYSIS ... 38
13.1 Potential issues of concern ... 38
13.2 Synthesis ... 39
APPENDIX 1 SCHEDULE OF ACTIVITIES ... 41
APPENDIX 2 STUDY SAMPLE ANALYSES ... 43
APPENDIX 3 EXAMPLE EMAIL FOR CONSENT OF LEGAL REPRESENTATIVE ... 44
APPENDIX 4 PROTOCOL AMENDMENT HISTORY ... 46
14. REFERENCES ... 47

LIST OF ABBREVIATIONS AND RELEVANT DEFINITIONS

Abbreviation	Definition
ABR	General Assessment and Registration form (ABR form), the application form that is required for submission to the accredited Ethics Committee; in Dutch: Algemeen Beoordelings- en Registratieformulier (ABR-formulier)
AE	Adverse Event
AR	Adverse Reaction
ARDS	Acute Respiratory Distress Syndrome
CA	Competent Authority
CCMO	Central Committee on Research Involving Human Subjects; in Dutch: Centrale Commissie Mensgebonden Onderzoek
COVID-19	Coronavirus disease caused by infection with severe acute respiratory syndrome coronavirus 2 (SARS-CoV-2)
CV	Curriculum Vitae
DSMB	Data Safety Monitoring Board
EU	European Union
EudraCT	European drug regulatory affairs Clinical Trials
EVLWi	Extravascular lung water indexed against predicted body weight or surface area
GCP	Good Clinical Practice
GDPR	General Data Protection Regulation; in Dutch: Algemene Verordening Gegevensbescherming (AVG)
IB	Investigator’s Brochure
IC	Informed Consent
IQR	Interquartile Range
IMP	Investigational Medicinal Product
IMPD	Investigational Medicinal Product Dossier
METC	Medical research ethics committee (MREC); in Dutch: medisch-ethische toetsingscommissie (METC)
PiCCO	Pulse Contour Cardiac Output. Used to measure a patient's haemodynamic status.
PVPi	Pulmonary vascular permeability indexed against predicted body weight or surface area
(S)AE	(Serious) Adverse Event

SPC	Summary of Product Characteristics; in Dutch: officiële productinformatie IB1-tekst
Sponsor	The Sponsor is the party that commissions the organisation or performance of the research, for example a pharmaceutical company, academic hospital, scientific organisation or investigator. A party that provides funding for a study but does not commission it is not regarded as the sponsor, but referred to as a subsidising party.
SUSAR	Suspected Unexpected Serious Adverse Reaction
UAVG	Dutch Act on Implementation of the General Data Protection Regulation; in Dutch: Uitvoeringswet AVG
WMO	Medical Research Involving Human Subjects Act; in Dutch: Wet Medisch-wetenschappelijk Onderzoek met Mensen

SUMMARY

Rationale: The SARS-CoV2 pandemic and resulting COVID-19 infection has led to a disruptive increase in the number of patients with ARDS. Acute respiratory distress syndrome (ARDS) is a severe, life-threatening medical condition characterised by widespread inflammation and vascular leak in the lungs. Although there is no proven therapy to reduce pulmonary vascular leak in ARDS, recent studies from our department have discovered that the tyrosine kinase inhibitor imatinib reinforces the endothelial barrier and prevents vascular leak in inflammatory conditions, while leaving the immune response intact. We hypothesize that imatinib mesylate limits the pulmonary oedema observed in ARDS due to COVID-19, and may thus help to reverse hypoxemic respiratory failure and to hasten recovery.

Objective: The current study aims to investigate the safety and effectiveness of imatinib mesylate solution for intravenous (iv) infusion in mechanically-ventilated subjects with COVID-19-related ARDS, where oral administration is not preferred due to unpredictable absorption.

Study design: Randomised, double-blind, parallel-group, placebo-controlled multi-centre clinical study of intravenous imatinib mesylate in 90 mechanically-ventilated subjects with COVID-19-related ARDS.

Study population: Subjects (>18years) admitted to the ICU for mechanical ventilation meeting the Berlin criteria for moderate-severe ARDS with a positive PCR for SARS-CoV2.

Intervention (if applicable): 1:1 randomisation to either imatinib 200mg b.i.d. (administered as an 8 mg/mL solution for i.v. infusion) or placebo (identical volume of solution for i.v. infusion [0.01M acetate buffer with 1.9% glycerol]) for 7 days, or until patient is discharged from the ICU, if earlier.

Main study parameters/endpoints: The primary study outcome is the change in Extra Vascular Lung Water Index (EVLWi) between baseline (Day 1) and Day 4. Secondary outcome parameters include change in oxygenation and ventilation parameters, length of invasive mechanical ventilation/ventilator-free days, length of ICU stay and mortality during a 28-day period after randomisation, as well as parameters of pathophysiology, safety, tolerability and pharmacokinetics.

Nature and extent of the burden and risks associated with participation, benefit and group relatedness: Participation involves randomisation to either the intervention or the placebo group. For efficacy evaluation, a PiCCO catheter will be placed and subjects will undergo regular EVLW measurements. For evaluation of pharmacokinetics and safety/tolerability subjects will undergo blood sampling at 5-7 time-points (at each time-point 1-4 tubes of 5mL will be drawn), and an ECG performed at Day 1, 2, 4, 7 and 10. Physical discomfort associated with participation involves possible side effects of the study medication, which are considered mild (predominantly gastro-intestinal discomfort). Anticipated benefits from the study medication involve faster resolution of pulmonary oedema and recovery from ARDS due to COVID-19 leading to a reduction in length of invasive mechanical ventilation and length of stay.

1. INTRODUCTION AND RATIONALE

1.1 Background of ARDS due to COVID-19

The COVID-19 pandemic has led to an unanticipated increase of the number of patients with ARDS admitted to the ICU, contributing to high morbidity and mortality, as well as an unprecedented consumption of medical resources. COVID-19 is caused by a coronavirus (scientific name: SARS-CoV-2), a non-segmented, positive sense RNA virus. Although most SARS-CoV-2 infections have an asymptomatic or mild course of disease (80%), COVID-19 has a detrimental course in a minority of patients (20%) [Wu 2020], particularly in older patients or patients with pulmonary or cardiovascular comorbidities [Zhou 2020]. In these cases, COVID-19 infection is characterized by damage to the alveolocapillary wall and extensive pulmonary capillary leak. The alveolar flooding results in impairment of oxygen diffusion and severe hypoxemic respiratory failure. In Chinese registries these cases of COVID-19 disease were classified as either 'severe' in case of low oxygen saturation or 'critical' when invasive mechanical ventilation was needed or multi-organ failure occurred [Wu 2020]. Radiological imaging demonstrates extensive ground glass opacities, consistent with alveolar oedema [Shi 2020].

Radiological as well as pathological examination [Shi 2020; Tuan 2020; Xu 2020] demonstrated that critical COVID-19 infections closely mimic Acute Respiratory Distress Syndrome (ARDS), a condition characterized by damage to the alveolo-capillary membrane by various insults [Matthay 2012]. Mortality in ICU treated *'critical'* COVID-19 patients is comparable to mortality in ARDS patients [Ferguson 2013; Yong 2013]. Of the patients admitted with COVID-19 infection to hospital, 17-35% develop ARDS, requiring ICU admission or even invasive mechanical ventilation (29-91%). According to recent reviews, mortality may mount up to 15-20% (hospitalized patients) to even 40% in ICU patients [Wiersinga 2020]. For these reasons, the current COVID-19 pandemic has resulted in a huge increase in the incidence of ARDS with a homogenous aetiology i.e., SARS-CoV2 infection.

According to the Berlin definition, ARDS is '*…an acute diffuse, inflammatory lung injury, leading to increased pulmonary vascular permeability, increased lung weight, and loss of aerated lung tissue…[with] hypoxemia and bilateral radiographic opacities, associated with increased venous admixture, increased physiological dead space and decreased lung compliance*' [JAMA 2012]. It is characterised by an acute onset, with bilateral infiltrates on chest imaging due to pulmonary oedema, and with severe hypoxemia despite mechanical ventilation. Pathophysiologically, ARDS results from an overwhelming inflammatory process involving alveolar epithelial and vascular endothelial injury in the lung which can be infective and non-infective in origin. The early phase of ARDS is characterized by alveolar flooding with protein-rich fluid due to increased vascular permeability. Pulmonary oedema then leads to the clinical manifestation of poor lung compliance, severe hypoxaemia, and bilateral infiltrates on chest radiograph. It also leads to alveolar epithelial injury of type I cells, which contributes further to the pulmonary oedema [Matthay, J Clin Invest 2012]. Despite decades of efforts, there is currently no registered drug to target pulmonary vascular permeability in ARDS [Matthay, Lancet Respir Med 2017].

Current treatment of patients with '*critical*' COVID-19 consists of supportive measures, including oxygen supplementation, diuretics and (non)-invasive mechanical ventilation.

Current guidelines prescribe dexamethasone [RECOVERY Collaborative Group 2020] and remdesivir [Beigel 2020] for patients with hypoxemia. Although remdesivir and dexamethasone target key pathophysiological processes of COVID-19 like viral replication and inflammatory damage, respectively, there is currently no proven benefit of pharmacological intervention to reverse pulmonary vascular leak and oedema.

1.2 Rationale for intervention

Studies from our department demonstrate that the anti-leukemic drug imatinib effectively and consistently protects against pulmonary vascular leak and alveolar oedema during inflammatory stimuli. In 2008, a patient with acute respiratory failure due to widespread pulmonary oedema was treated with imatinib mesylate for another indication than the acute respiratory failure itself. However, initiation of the imatinib treatment (200mg/day) was followed by a surprisingly fast reversal of respiratory failure. The reversal of respiratory failure was paralleled by decrease in pulmonary oedema on radiological imaging, resulting in the hypothesis that imatinib directly protects the pulmonary endothelial barrier. This unexpected effect of imatinib was published in 2008 [Overbeek 2008]. The hypothesis was tested in an extensive preclinical study, evaluating the effect of imatinib on endothelial barrier function in several conditions. These studies demonstrated that imatinib protects the endothelial barrier under inflammatory conditions, both in in vitro models using several types of endothelial cells and various inflammatory mediators, and in vivo, using various animal models for vascular leak [Aman 2012]. The protective effects of imatinib were confirmed by several studies by independent research groups. These effects are reviewed in [Rizzo 2015]; a summary is provided in Table 1.

After the publication of the first case report [Overbeek 2008] and the preclinical evidence for a protective effect of imatinib on the endothelial barrier [Aman 2012], imatinib (300mg/day) was associated with clinical improvement of acute respiratory failure in 1 patient in an independent hospital [Carnevale-Schianca 2011]. In addition, imatinib has been used in a compassionate use setting in 2 additional patients in our hospital – in both cases initiation of imatinib therapy (200-400mg/day) was followed by reversal of vascular leak and/or respiratory failure [Aman 2013, 2 unpublished cases]. Although these cases should be interpreted with caution, they consistently suggest a beneficial effect of imatinib on pulmonary vascular leak and/or respiratory failure (Table 1). To date, one case report using oral imatinib to successfully treat COVID-19 pneumonia has been published [Morales-Ortega 2020]. The protective effects of imatinib at the endothelial level 2-10μM were found at concentrations that are comparable to plasma levels found in patients treated with imatinib for CML (2-5μM) [Aman 2012], indicating that regular dosing schemes of imatinib are sufficient to induce its protective effect on the endothelial barrier. Indeed, data from the COUNTER-COVID study, which evaluates the effect of oral imatinib mesylate in hospitalized patients with COVID-19, demonstrate that dosing of 400mg/day orally is sufficient to reach target levels described above [Bartelink 2021], and that treatment with 400mg/day resulted in reduced mortality, duration of mechanical ventilation and ICU length of stay [Aman 2021].

Based on our observations that the optimal protective effect of imatinib on the endothelial barrier ranges between 2-10μM, together with previous pharmacokinetic studies after intravenous imatinib [Peng 2004], we propose a dosing scheme of 200mg b.i.d. This dosing scheme was shown to provide plasma levels that correspond to the 2-10μM range found

in our in vitro studies. The current study will evaluate the effect of intravenous imatinib on pulmonary oedema as measured by EVLWi. Several arguments drive the use of intravenous imatinib in the current study: 1) since most ICU patients are intubated, oral administration is suboptimal. 2) Intestinal uptake in mechanically ventilated ICU patients is often impaired due to intestinal oedema. 3) by intravenous administration, imatinib directly reaches the target organ, i.e., the endothelium, requiring lower dosing and fewer side-effects. A detailed analysis on dosing and route of administration is provided in the Investigators Brochure.

Concerning safety, imatinib was shown to have mild or no effects on the immune response. Although developed to target leukemic cells, imatinib hardly affects healthy leucocytes [Deininger 1997] and a normal lymphocytic response was observed in lymphocytes from patients treated with imatinib [Maggio 2011]. Of particular relevance for the current protocol, it was shown that treatment with imatinib does not affect the control of primary viral infections [Mumprecht 2006]. We demonstrated in a healthy volunteer model of lung injury that imatinib did not affect the immune response to LPS inhalation in healthy human subjects [Unpublished data]. Finally, the COUNTER-COVID study which tested oral imatinib in COVID-19 patients admitted to hospital with hypoxemia, did not reveal safety concerns [Aman 2021].

Altogether, substantial evidence indicates that imatinib is an ideal candidate for treatment of the pulmonary complications of SARS-CoV2 like ARDS, since it protects against vascular leak and alveolar oedema thereby reducing hypoxemic respiratory failure. The current study seeks to directly measure the effect of intravenous imatinib on pulmonary vascular leak in COVID-19 ARDS. COVID-19 offers a homogenous cause of ARDS, however, the available evidence indicates that ARDS due to other causes will respond similarly to imatinib. Therefore, the identification of a compound that reverses pulmonary vascular leak may benefit well beyond COVID-19 ARDS and provide a first treatment for other forms of ARDS as well.

Table I

Model	Effect of imatinib	Reference
***In vitro* studies**		
Rat aortic endothelial cells	Protects endothelial barrier	Kurimoto 2004
Human umbilical vein endothelial cells	Protects endothelial barrier<br>Improves cell-matrix adhesion	Aman 2012
Human lung microvascular endothelial cells	Protects endothelial barrier	Aman 2012
Immortalized endothelial cells	Protects endothelial barrier	Chislock 2013
Human umbilical vein endothelial cells	Protects endothelial barrier	Kim 2013
Mouse lung microvascular endothelial cells	Protects endothelial barrier	Stephens 2015
***In vivo* studies**		
Bleomycin-induced lung injury	Anti-inflammatory<br>Anti-fibrotic	Rhee 2011
Isolated perfused lung model (mouse)	Inhibits lung vascular leak	Aman 2012
Miles assay (mouse)	Attenuates vascular leak in skin	Aman 2012
Cecal Ligation & Puncture (Sepsis) (mouse)	Attenuates vascular leak in lungs, kidneys	Aman 2012
Intratracheal LPS (mouse)	Attenuates pulmonary oedema	Kim 2013
Ischemia-reperfusion in reperfusion lung	Reduces endothelial cytotoxicity	Stephens 2015
Miles assay (mouse)	Attenuates vascular leak in skin	Chislock 2013
Intratracheal LPS (mouse)	Attenuates vascular leak and inflammation	Letsiou 2015
Cardiac bypass surgery	Attenuates vascular leak, improves perfusion, improves oxygenation	Koning, BJA 2016
**Clinical case-reports (human)**		
Pulmonary veno-occlusive disease	Resolution of pulmonary oedema<br>Improvement of oxygenation	Overbeek 2008
Bleomycin-induced pneumonitis / lung injury	Resolution of pulmonary oedema	Carnevale-Schianca 2011
Idiopathic pulmonary vascular leak	Resolution of generalized oedema<br>Reduction of pulmonary vascular leak	Aman 2013
Severe systemic capillary leak syndrome	Resolution of generalized oedema	Unpublished
Pulmonary graft versus host disease with GGO and severe hypoxemia	Improvement of SaO2, weaning from ventilator support	Unpublished
Drug-induced pneumonitis	Resolution of pneumonitis, case series	Landberg, Acta Oncol 2018

2. OBJECTIVES

Primary:

- Efficacy: To evaluate the effect of intravenous imatinib compared to standard of care on limiting development of extravascular lung water in invasively mechanically-ventilated subjects with COVID-19-related ARDS.

Secondary:

- Efficacy: To evaluate the effect of intravenous imatinib compared to standard of care on patient outcomes in mechanically-ventilated subjects with COVID-19-related ARDS.
- Safety: To evaluate the safety and tolerability of intravenous imatinib compared to standard of care in mechanically-ventilated subjects with COVID-19-related ARDS
- Pharmacokinetics: To determine imatinib pharmacokinetics in subjects with COVID-19-related ARDS.

3. STUDY DESIGN

This is a randomised, double-blind, parallel-group, placebo-controlled, multi-centre clinical study comparing intravenous imatinib mesylate with placebo in invasively mechanically-ventilated subjects with COVID-19-related ARDS. The study will enrol 90 subjects (45 subjects/treatment arm; see sample size calculation). In this two-arm study, eligible subjects will be randomly allocated to receive imatinib mesylate or placebo in a 1:1 ratio. Importantly, the inclusion will be extended to all-cause ARDS if recruitment drops to below a predefined threshold (see inclusion criteria). Besides the randomisation to treatment of placebo, all subjects will be cared for using local treatment protocols as standard of care. At each of the participating centres, standard treatment protocols may include: COVID-19-specific medication (e.g. dexamethasone, tocilizumab/sarilumab, anti-COVID antibodies), low tidal volume ventilation, conservative fluid management and prone positioning in case of persistently low PaO2/FiO2.

4. STUDY POPULATION

4.1 Population (base)

The research population will be recruited from subjects admitted to the ICU with moderate-severe COVID-19 ARDS, as defined by the Berlin criteria. Patient recruitment will take place in the AUMC, locations AMC and VUMC, the OLVG location Oost (Amsterdam), Dijklander Ziekenhuis (Hoorn/Purmerend) and the Erasmus MC (Rotterdam). Additional locations can be considered to support recruitment.

4.2 Inclusion criteria

In order to be eligible to participate in this study, a subject must meet all of the following criteria:

1. Age ≥ 18 years;
2. Moderate-severe ARDS, as defined by Berlin definition for ARDS (Table 1), and intubated for mechanical ventilation.
3. PCR positive for SARS-CoV2 within the current disease episode.
4. Provision of signed written informed consent from the patient or patient's legally authorised representative;

Table 1 Berlin definition of acute respiratory distress syndrome

Timing	Within 1 week of a known clinical insult or new or worsening respiratory symptoms
Chest imaging[a]	Bilateral opacities — not fully explained by effusions, lobar/lung collapse, or nodules
Origin of oedema	Respiratory failure not fully explained by cardiac failure or fluid overload.
	Need objective assessment (e.g., echocardiography) to exclude hydrostatic oedema if no risk factors present
Oxygenation[b]	
Mild	200 mmHg < PaO_2/FIO_2 ≤300 mmHg with PEEP or CPAP ≥5 cmH_2O[c]
Moderate	100 mmHg < PaO_2/FIO_2 ≤200 mmHg with PEEP ≥5 cmH_2O
Severe	PaO_2/FIO_2 ≤100 mmHg with PEEP ≥5 cmH_2O

Abbreviations: CPAP, continuous positive airway pressure; FIO2, fraction of inspired oxygen; PaO_2, partial pressure of arterial oxygen; PEEP, positive end-expiratory pressure;
[a] Chest radiograph or computed tomography scan;
[b] If altitude is higher than 1,000 m, the correction factor should be calculated as follows: [PaO_2/FIO_2 - (barometric pressure/760)];
[c] This may be delivered noninvasively in the mild acute respiratory distress syndrome group.

Reproduced from: ARDS Definition Task Force, Ranieri VM, Rubenfeld GD, et al. Acute respiratory distress syndrome: the Berlin Definition. JAMA 2012;307:2526-33

4.3 Exclusion criteria

A potential subject who meets any of the following criteria will be excluded from participation in this study:

1. Persistent septic shock (>24h) with a Mean Arterial Pressure (MAP) ≤ 65 mm Hg and serum lactate level > 4 mmol/L (36 mg/dL) despite adequate volume resuscitation and vasopressor use (norepinephrine > 0.2 µg/kg/min) for > 6 hours;
2. Pre-existing chronic pulmonary disease, including:
   - Known diagnosis of Interstitial Lung disease
   - Known diagnosis of COPD GOLD Stage IV or FEV_1<30% predicted
   - DLCO <45% (if test results are available)
   - Total lung capacity (TLC) < 60% of predicted (if test results are available);
3. Chronic home oxygen treatment;
4. Pre-existing heart failure with a known left ventricular ejection fraction <40%;
5. Active treatment of haematological or non-haematological cancer with targeted immuno- or chemotherapy, or thoracic radiotherapy in the last year;
6. Currently receiving extracorporeal life support (ECLS);
7. Severe chronic liver disease with Child-Pugh score > 12;
8. Subjects in whom a decision to withdraw medical care is made (e.g. palliative setting);
9. Inability of the ICU staff to initiate IMP administration within 48 hours of intubation;
10. Known to be pregnant or breast-feeding;
11. Enrolled in a concomitant clinical trial of an investigational medicinal product;
12. White blood count < $2.5x10^9$/l;
13. Haemoglobin < 4.0 mmol/l;

14. Thrombocytes < $50x10^9/l$;
15. The use of strong CYP3A4 inducers, including the following drugs:
    - Carbamazepine, efavirenz, enzalutamide, fenobarbital, fenytoine, hypericum, mitotaan, nevirapine, primidon, rifabutine, rifampicine;
16. The presence of an intra-aortic balloon pump (IABP).
17. Known medical history of aortic aneurysm in the trajectory of the PiCCO measurement between central venous line and arterial detector.
18. Known medical history of an intracardiac shunt.

4.4 Sample size calculation

The number of subjects scheduled for inclusion in the study is 90, including 45 subjects in the placebo arm and 45 subjects in the imatinib mesylate arm.

Sample size calculations were done with the formula: zα/2 - zπ = (n/2)1/2 * (μ1 – μ2)/σ, in which zα/2-zπ = 2,8 (α = 5% en π = 80%). The change (Δ) in EVLWi between Day 0 and Day 4 is the primary outcome. We expect the baseline EVLWi to be around 17ml/kg, as previously described for patients with moderate-severe ARDS [Kushimoto 2013; Kaneko 2014]. In the placebo group we expect the ΔEVLW Day 0 and Day 4 to be 0.5 (μ1), based on previously literature from collaborating groups [Perkins 2006; Craig 2011]; in the imatinib group we expect the ΔEVLWi to be -4 (μ2). This is considered a clinically relevant difference as this difference was found to independently predict ARDS mortality in another clinical study [Brown, Ann Intens Care 2013]. The expected treatment effect is based on the imatinib effect observed in preclinical data: 25% reduction in vascular leak [Aman 2012]. The sigma (σ) for ΔEVLWi is set at 7.0, based on previous EVLWi studies [Craig 2011; Kaneko 2014]. Using the equation above, this yields 76 subjects with 38 subjects/arm. Taking into account a drop-out rate of 15%, a total of 90 subjects will be recruited. A blinded sample size re-estimation may be performed if patient recruitment falls to an unacceptably low level. This may reduce the overall number of patients needed to be recruited into this study. This blinded SSR would be fully described in the statistical analysis plan.

5. TREATMENT OF SUBJECTS

5.1 Investigational product/treatment

Subjects will undergo 1:1 randomisation to receive intravenous imatinib (200mg bid) or placebo for 7 days.

5.2 Use of co-intervention (if applicable)

There are no co-interventions

5.3 Escape medication (if applicable)

Not applicable

5.4 Interactions

Imatinib is a substrate for CYP3A4 and P-gp. It inhibits CYP3A4, CYP2D6 and CYP2C9. Most relevant interactions for our study population include:

- Strong induction of CYP3A4. The following drugs are contra-indicated for the study: Carbamazepine, efavirenz, enzalutamide, phenobarbital, phenytoin, hypericum, mitotane, nevirapine, primidone, rifabutin, rifampicin.

- CYP3A4 inhibition. In general, imatinib is relatively insensitive to CYP3A4 inhibition, as imatinib may rely on enzymes other than CYP3A4 for its metabolism. Therefore, there is no need for adjustment, but recording of the following drugs in the eCRF is important: azoles.
- Imatinib may change the concentration of other CYP3A4, CYP2D6 and CYP2C9-dependent drugs, including anticoagulants, cyclosporine, simvastatin etc.

All co-medication will be registered.

6. INVESTIGATIONAL PRODUCT

6.1 Name and description of investigational product(s)

- Imatinib: Isotonic sterile solution of Impentri (imatinib) 8mg/ml, equivalent to 9.557 mg/mL of imatinib mesylate, buffered with 0.01M acetate (adjusted to pH 5.0) and the tonicity adjustment is by use of glycerol (1.9% v/v).
- Placebo: Isotonic sterile solution buffered with 0.01M acetate (adjusted to pH 5.0) and the tonicity adjustment is by use of glycerol (1.9% v/v).

6.2 Dosage and Administration

A 25ml volume of IMP will be administered over 2-hours as an intravenous infusion. This corresponds to a dose of 200mg imatinib (100mg/h), or 25ml placebo (12.5ml/h). Treatment will be administered twice daily (400 mg total daily imatinib dose) for up to 7 days, or until patient is discharged from critical care, if earlier. The first dose of IMP will occur on Day 1, as soon as possible after randomisation. IMP should be administered 12h (± 2h) apart between 06:00 - 12:00 in the morning and 18:00 - 24:00 in the evening. On Day 1, the first dose of IMP may be administered between 04:00 and 12:00 (08:00± 4h) and the second dose between 16:00 and 24:00 (20:00± 4h). Patients commencing treatment after 12:00 on Day 1 should receive only one dose of IMP (between 16:00 and 24:00).

Administration should be by a separate central venous catheter, which is part of standard patient care. During administration of the IMP, concomitant medication should not be given via the same central venous catheter but via other indwelling venous catheters.

Full details on the formulations are enclosed in the Investigational Medicinal Product Dossier (IMPD). The IB and IMPD will be available to the Site Investigator and will also be kept on file in the trial master file (TMF).

6.3 Summary of findings from non-clinical studies

Protection against vascular leak: We have demonstrated that imatinib protects against disruption of the endothelial barrier under inflammatory conditions in a concentration-dependent fashion, with an optimal effect ranging between 2-10μM. These effects were observed in various types of endothelial cells, and during stimulation with various inflammatory stimuli. We demonstrated that imatinib exerts its protective effect by inhibition of Abl tyrosine kinases. In this study, the protective effects of imatinib were validated in three different models of vascular leak, including vascular leak in the lungs [Aman 2012]. These preclinical studies were repeated and validated by several independent research groups (see Table I).

Antiviral effects: Various high-impact preclinical studies have shown that Abl kinases are key mediators of viral replication, e.g., demonstrating that Abl1 kinases drives replication

of Ebola virus [Garcia 2012], and that Abl2 kinases drives replication of coronavirus like MERS and SARS [Coleman 2016]. In both studies, imatinib was shown to reduce viral replication, albeit at high concentrations. Likewise, imatinib was demonstrated to have antiviral activity in Poxvirus infection *in vitro* and *in vivo* [Reeves 2005]. A recent drugs screen testing clinically available compounds on SARS-CoV2 replication, demonstrated imatinib as one of the strongest inhibitors of SARS-CoV2 [Han 2020]. These data suggest that different virus species use common pathways for host cell infection, in which Abl kinases play a central role.

Safety: Imatinib was shown to have mild or no effects on the immune response. Although developed to target leukemic cells, imatinib hardly affects healthy leucocytes [Deininger 1997] and a normal lymphocytic response was observed in lymphocytes from patients treated with imatinib [Maggio 2011]. In mice, it was shown that treatment with imatinib does not affect the control of primary viral infections [Mumprecht 2006].

A detailed description of preclinical evidence is provided in the Investigators Brochure.

6.4 Summary of findings from clinical studies

Protection against vascular leak: Case-based evidence: As discussed in the Rationale (§1), we have demonstrated that initiation of imatinib treatment was associated with fast resolution of alveolar oedema in a patient with pulmonary veno-occlusive disease that was treated with imatinib for another indication [Overbeek 2008]. In a second clinical case, a patient was successfully treated with imatinib for capillary leak syndrome. [Aman 2013]. Also reports from other groups have shown that imatinib gave fast resolution of drug-induced pneumonitis [Carnevale-Schianca 2011] or acute interstitial pneumonia [Fenocchio 2016] (both syndromes mimicking ARDS). Recently, a case series was published showing that imatinib effectively reversed pneumonitis in patients suffering from chemotherapy-induced pneumonitis [Landberg 2018]. Addendum October 2021: in a large randomized clinical trial, imatinib was shown to significantly reduce mortality and the length of mechanical ventilation [Aman 2021].

Antiviral effect: During the current COVID-19 pandemic, two Italian registries demonstrated that incidence of COVID-19 was lower than expected in a cohort of Philadelphia chromosome positive patients with Acute Lymphocytic Leukemia (Ph+ ALL) [Foa 2020] and in a cohort of CML patients treated with imatinib [Breccia 2020]. Although the protective effect suggested by these studies is prone to bias, these studies indicate that the use of imatinib does not confer a higher susceptibility for COVID-19 or for a worse outcome.

Safety: A human volunteer study has investigated the effect of oral imatinib during a human experimental model of lipopolysaccharide (LPS) inhalation. In this study, volunteers were pre-treated with imatinib 400mg/day for 4 days, followed by inhalation of LPS. In this study, a normal immune response was observed, and no safety issues were met [NCT03328117]. The COUNTER-COVID study tests the effect of oral imatinib in patients with COVID-19 pneumonitis, admitted to the ward. Although the double-blind nature of the COUNTER-COVID study precludes preliminary efficacy analyses, the first three DMSB meetings have not revealed any safety issues thus far (i.e., after inclusion of up to 155 patients).

Intravenous infusion: In 2004, a study tested the feasibility, tolerability and pharmacokinetics of imatinib in healthy volunteers. Apart from irritation at the infusion site, no adverse events were reported [Peng 2004].

6.5 Summary of known and potential risks and benefits

Common side effects of imatinib include flushing, cough, flatulence, gastro-oesophageal reflux, and gastritis. Relevant uncommon side effects include palpitations, cardiac failure, pleural effusion, acute renal failure, melena, chest pain and pancytopenia (although the pancytopenia was observed in patients in CML was shown to result from apoptosis of leukemic cells, and that repopulation with non-affected leucocytes was undisturbed under imatinib treatment). In general side effects are mild, and usually occur after chronic use (i.e., starting from 2 weeks and onwards). As the imatinib treatment in this study is relatively short (days) compared to the chronic use in CML (months-years), we anticipate that the side effects observed in CML studies are less frequent in the study proposed here. An extensive discussion of relevant side effect and imatinib toxicity is provided in the investigator's brochure. Altogether, the potential benefit of preventing mechanical ventilation and reducing health care consumption outweighs the mild side effects observed in imatinib use.

6.6 Description and justification of route of administration and dosage

Justification for route of administration: Imatinib will be administered intravenously, since the target population includes subjects admitted to the ICU who are intubated and ventilated. This condition not only hampers oral administration – only nasogastric feeding is possible – but is also associated with impaired bioavailability due to e.g., gastroparesis and intestinal oedema. In addition, during intravenous administration, imatinib directly reaches the target organ, i.e., the endothelium, requiring lower dosing and less side-effects.
Justification for dose: Twice daily intravenous administration of 200mg imatinib infused over 2 hours has been selected for investigation in this clinical study. This dose is deemed sufficiently high to maintain a suitably large therapeutic window, yet not be too high that there is a risk of effects which would not be relevant at therapeutic doses are uncovered. The 200mg dose has been selected with reference to non-clinical studies with imatinib, and published pharmacokinetic, pharmacodynamic and safety data for oral imatinib in the oncology setting. This dose (200mg bid) equals the daily dose tested in the COUNTER-COVID study, in which no safety issues were met thus far (EudraCT 2020-001236-10, based on DSMB safety analysis after inclusion of 155 patients). The infusion rate (100mg/h) does not exceed the infusion rate that was tested and found safe in healthy volunteers [Peng 2004]. Additional details on the justification of the intravenous dose are provided in the Investigators Brochure §5.6.

6.7 Dose modifications

No dose modifications are permitted. Should the patient experience any intolerable side-effects that are considered related to the IMP, treatment should be discontinued and the patient withdrawn – see §8.5. The reason should be recorded in the CRF.

6.8 Preparation and labeling of Investigational Medicinal Product

The investigational medicinal product (IMP) is presented within a 50ml clear glass vial with a chlorobutyl stopper and aluminium flip-off seal. Each vial contains 320mg of imatinib per 40mL (imatinib 8mg/ml, equivalent to 9.557mg/ml of imatinib mesylate) or placebo.
Medication labels for each vial containing imatinib and placebo solution for infusion will be in the local language and comply with the legal requirements for Netherlands. They will include storage conditions for the drug and the kit identification number, with a space to record the Patient ID code after the IMP is assigned.
IMP will be supplied by Exvastat to the Pharmacy of the AUMC, location AMC (Dr. N. van

Rein, Pharmacist), for distribution to participating centres. Additional dispensing instructions will be provided in the Pharmacy Manual.

6.9 Treatment compliance

The dose, timing, and mode of IMP administration may not be changed. Any departures from the intended regimen must be recorded in the case report form (CRF).

Each dose of candidate agent will be administered by a member of the clinical study team that is qualified and licensed to administer the study product. Date and time of each administration will be entered into the CRF.

6.10 Drug accountability

On receipt, all IMP will be transferred to a secure storage area, appropriately environmentally controlled, and batch numbers and quantities recorded in an inventory. All participating centres have a satellite pharmacy in the ICU ward, appropriate for storage and preparation of the study medication. Usage of materials will be recorded on Batch Manufacturing Records generated for each production batch.

At the close of the study, reconciliation will be performed by comparison between residual quantities, usage, and the initial inventory. Reconciliation of numbers of unused manufactured dosage units will be performed by comparison between Batch Manufacturing Records and Dosage Administration Records. Unused dosage units will be transferred to secure storage. Usage of correct quantities is assured by verification of the administered dose at the time of dosing.

After reconciliation, all unused drugs, placebos, devices and dosage units will be transferred to the central pharmacy of the sponsor or destroyed under supervision. The storage conditions are temperature controlled and monitored.

The pharmacist shall maintain records of the IMP delivery to the site, an inventory at the site, the distribution to each subject, and the return to the sponsor or alternative disposition of unused IMP. These records will include dates, quantities received, batch / serial numbers, expiration dates, and, for each dose of IMP dispensed to a subject, the subject's unique Patient ID code. Investigators and /or the local site QP will maintain records that document adequately that subjects were provided with the correct study medication. These records will be part of each patient’s eCRF.

7. NON-INVESTIGATIONAL PRODUCT

Not applicable (NA)

8. METHODS

8.1 Study parameters/endpoints

8.1.1 Main study parameter/endpoint

The main study parameter is the change in extravascular lung water index (EVLWi) between baseline (Day 1) and Day 4. See §8.3.

8.1.2 Secondary study parameters/endpoints (if applicable)

Pulmonary oedema, gas-exchange and respiratory mechanics:

- EVLWi (Day 1, 2, 4 and 7)
- Pulmonary vascular permeability index (PVPi: Day 1, 2, 4 and 7)
- Oxygenation index (OI: Day 1, 2, 4, 7, 10 and Day 28, if available)
- PaO2/FiO2 ratio (Day 1, 2, 4, 7, 10 and Day 28, if available)
- Airway driving pressure (Day 1, 2, 4, 7, 10 and Day 28, if available)
- Compliance of the respiratory system (Day 1, 2, 4, 7, 10 and Day 28, if available)
- Mechanical power (Day 1, 2, 4, 7, 10 and Day 28, if available)

Inflammation, endothelial injury and lung epithelial injury:

- Plasma biomarkers of inflammation, endothelial injury and lung epithelial injury (Day 1, 2, 4, 7 and 10)

Organ function and outcome:

- Sequential Organ Failure Assessment (SOFA) score (Day 1, 2, 4, 7, 10 and Day 28, if available);
- Number of ventilator-free days and alive at Day 28;
- Duration of mechanical ventilation (days) between Day 1 and 28;
- Length of ICU stay (days) between Day 1 and 28;
- Hospital length of stay (days) between Day 1 and 28;
- 28-day mortality.
- 9-point WHO ordinal Scale for Clinical Improvement

Safety parameters

- Blood cell count, i.e., haemoglobin, thrombocytes and leucocytes (Day 1, 2, 4, 7 and 10);
- Kidney function, estimated glomerular filtration rate, sodium and potassium (Day 1, 2, 4, 7 and 10);
- Liver enzymes, i.e., AST, ALT, Alkaline Phosphatase, γ-glutamyl transferase, bilirubin (Day 1, 2, 4, 7 and 10);
- NT-proBNP (Day 1, 2, 4, 7 and 10);
- SAEs / AE;
- Corrected QT interval at ECG (Day 1, 2, 4, 7 and 10).

Pharmacokinetics

- Day 1: Imatinib, albumin and AGP plasma levels at 4h and between 7h and 8h after start of IMP infusion. If for logistical reasons PK sampling cannot be performed at these times on Day 1, PK samples should be collected 3h and 7h after the first IMP infusion on Day 2 instead.
- Day 1 for a subgroup of patients: Imatinib, albumin and AGP plasma levels during IMP infusion and 2h after the start of the infusion (i.e., at the end of the infusion).
- Day, 2, 4 and 7: Imatinib, albumin and AGP plasma levels pre-IMP infusion (am or pm

dose).

Thoracic ultrasound

- Thoracic ultrasound will be performed at Day 1 and 4 in a sub-group of patients.

8.1.3 Other study parameters (if applicable)

- Baseline demographics (age, sex, intoxications);
- Medical history and comorbidity;
- Vital parameters;
- Use of other drugs;
- COVID-19 vaccination status

8.2 Randomisation, blinding and treatment allocation

Randomisation: Before randomisation, the study physician will check all inclusion and exclusion criteria and will enters these in the web-based application (Castor). Screening and placement of the PiCCO catheter is performed as part of a deferred consent procedure. For subjects who meet the eligibility criteria, the patient's legal representative will be asked for informed consent (§11.2.2). When informed consent is provided, subjects will be randomized 1:1 to receive placebo or imatinib. Randomisation will take place via Castor using variable block sizes with stratification per participating centre. The pharmacist will check relevant drug interactions before dispensing medication.

Blinding: This is a double-blind study - Subjects, clinical staff (nurses and physicians) and investigators will be blinded for study medication. Blinding will be guaranteed be covering the syringes containing the IMP so that the content will not be visible from the outside.
Code breaking is allowed in any of the following circumstances:

- Treatment of an individual in a medical emergency where knowledge of the treatment allocation is required.
- In the event of a Suspected Unexpected Serious Adverse Reaction (SUSAR) the subject will be unblinded, if this is required for treatment of the SUSAR.
- In the event that the ICU staff / researcher is accidentally exposed to study medication.

Pregnancy testing will be performed in all eligible premenopausal subjects before inclusion in the study.

8.3 Study procedures

Unless otherwise specified, all Day 1 assessments should be performed before the first dose of IMP.

Some study procedures are scheduled during the deferred consent window. With the exception of administration of IMP, placement of a PiCCO catheter and pharmacokinetic

blood sampling, all other procedures are part of routine patient monitoring, for which consent is not required. The PICCO catheter will replace a standard care arterial line, while PK blood samples will be obtained from standard care venous lines inserted in all patients. Deferred consent and handling of data obtained from subjects who subsequently fail to consent are described in §11.2.2.

Extravascular lung water (EVLW) measurements

In the deferred consent time window, a PiCCO catheter [Pulsion Medical Systems, Feldkirchen, Germany] will be placed in the radial or femoral artery. This will replace a standard of care arterial line, and will not pose additional burden on top of standard care. For EVLW measurements, a 20mL bolus of 0.9% saline at 4°C will be injected via a jugular/subclavian vein central venous catheter into the right atrium and the thermodilution curve recorded in the aorta used to calculate intrathoracic blood volume index and EVLW. The thermodilution curve will also provide the pulmonary vascular permeability (PVP). The average result from three (if within 15% variance) or five 20mL bolus injections will be used for each measurement. Both EVLW and PVP will be indexed against predicted body weight or body surface area (EVLWi and PVPi) [Hofkens, 2014]. EVLW and PVP measurements will be performed on Day 1-7, the difference (delta) between Day 1 and Day 4 will be used for the primary outcome. Further details on the EVLW measurements are provided in a Standard Operations Procedure for PiCCO measurements.

Blood sampling

- 1 EDTA tubes (of 5mL) and 1 heparin-gel tube (of 5mL) will be drawn on Day 1, 2, 4, 7 and 10 for clinical safety laboratory measurements.
- 1 Paxgene tube (of 2.5mL) will be drawn on Day 1 and 4 to serve as study material.
- A lithium heparin tube (5mL) will be drawn for PK at 4h and between 7h or 8h after the start of IMP infusion on Day 1. If for logistical reasons PK sampling cannot be performed at these times on Day 1, PK samples should be collected 3h and 7h after the start of the first IMP infusion on Day 2 instead. In addition, PK samples will be collected pre-dose (am or pm dose) on Day 2, 4 and 7. In a subgroup of subjects, a lithium heparin tube (5mL) will also be drawn during IMP infusion and 2h after the start of IMP infusion (i.e., at the end of the infusion) on Day 1 for pharmacokinetic measurements.
- A lithium heparin tube (5mL), a citrate tube (5mL) and a serum gel tube (5mL) will be drawn on Day 1, 2, 4, 7 and 10 to serve as study material.
- Arterial blood gas on Day 1, 2, 4, 7 and 10.

The serum and plasma samples (referred to as 'study material') will be used for analyses of pharmacokinetics, immune responses, inflammatory parameters and parameters of endothelial and epithelial injury. These analyses will largely be performed after the last study subject has completed the study. An overview of the analyses is provided in Appendix 2.

Clinical Safety Laboratory Tests

The following laboratory evaluations will be done at the local laboratories at screening and on Day 1, 2, 4, 7 and 10:

- Haematology, i.e., haemoglobin, thrombocytes and leucocytes

- Biochemistry: Creatinine, estimated glomerular filtration rate, sodium and potassium, AST (SGOT), ALT (SGPT), Alkaline Phosphatase, γ-glutamyl transferase, bilirubin, NT-proBNP.

Electrocardiogram (ECG):
An ECG will be taken at screening and at Day 1,2,4,7 and 10 and the corrected QT interval (QTc) recorded in the CRF. Any other clinically significant ECG abnormalities will be captured in the patient’s electronic health record and, if applicable, as an SAE (See §9.2.2).

Phone call
In case subjects are discharged from the hospital before Day 28, subjects will be contacted by phone (Day 28 – 31) to monitor clinical status. During the phone call the patient's clinical status will be recorded according to the 9-point WHO COVID19 ordinal scale of clinical improvement. In case patients are still hospitalized at Day 28, the clinical status according to the 9-point WHO COVID19 ordinal scale of clinical improvement will be obtained from the patient’s electronic health record, together with vital parameters, ventilator settings (in case of mechanical ventilation) and concomitant medication.

An overview of study interventions is provided in Appendix 1.

8.4 Concomitant Medication & Supportive Care

8.4.1 Concomitant Medication

Subjects included in the study will be otherwise treated by the ICU staff not involved in the study, according to institutional and international guidelines. Throughout the study Investigators/Treating Physicians may prescribe any concomitant medications or treatments deemed necessary to provide adequate supportive care. Concomitant medications prescribed for the patient will be reviewed daily whilst the patient is in the ICU. Use of anti-coagulants, systemic steroids, drugs that inhibit CYP3A4, antibiotics, tocilizumab or other clinically relevant medications (e.g., vasopressors or drugs used to treat AEs) will be recorded daily in the CRF between Day 1 and Day 10.

8.4.2 Concomitant Supportive Care

Supportive measures for ARDS include lung-protective ventilation [ARDS Network 2000] and, where indicated, ventilation in prone-positioning [Guerin 2013], treatment with neuromuscular blockers [Papazian 2010], extracorporeal membrane oxygenation (ECMO) [EOLIA 2018; Schmidt 2020], and restrictive fluid management [Wiedemann 2006], may be applied at the treating physicians’ discretion. Application of prone-positioning, neuromuscular blockers or extra-corporeal membrane oxygenation (ECMO) should be recorded in the eCRF, together with the duration of the procedure in hours. Directions for concomitant supportive care include:

8.4.2.1 Ventilator settings

Ventilation setting should in accordance with existing guidelines for subjects with ARDS. This minimally consists of low tidal volumes (±6 ml/kg PBW), and titrated PEEP levels.

Also, a low driving pressure could be considered.

8.4.2.2 Oxygenation targets

The oxygenation target ranges for SpO2 and PaO2 are 92% to 96%, and 8 kPa to 11.5 kPa, respectively.

8.4.2.3 Sedation

Sedation follows the local guidelines for sedation in each participating unit. In general, these guidelines favor the use of analgo–sedation over hypno–sedation, use of bolus over continuous infusion of sedating agents, and the use of sedation scores.

8.4.2.4 Fluid balance

A fluid balance targeted at normovolemia and a diuresis of ≥ 0.5 ml/kg/hour should be maintained, with low-threshold use of furosemide to maintain normovolemia. Crystalloid infusions are preferred over colloid infusions.

8.5 Withdrawal of individual subjects

Each subject has the right to withdraw from the study at any time. Subjects may be withdrawn from the study either at their own request, at the request of their proxy or at the discretion of the Investigator. The subjects will be made aware that this will not affect their future care. Subjects will be made aware (via the information sheet and consent form) that should they withdraw the data collected to date cannot be erased and may still be used in the final analysis.

The Investigator may withdraw a subject from the study at any time if he/she considers that the subject's health is compromised by remaining in the study or the subject is not sufficiently cooperative.

The reasons for any subject withdrawal will be recorded on the study completion form of the CRF.

Subjects withdrawn for reasons attributed to the study treatment will not be replaced. All other withdrawals will be replaced at the discretion of the Sponsor, following discussion with the Investigator.

In the event of any abnormalities considered to be clinically significant, the Investigator will decide upon the appropriate course of action. In addition, the investigator may discontinue a subject from the study at any time if the investigator considers it necessary for any reason including:

- Ineligibility (either arising during the study or retrospective having been overlooked at screening)
- Significant protocol deviation
- Significant non-compliance with treatment regimen or study requirements
- An adverse event which requires discontinuation of the study medication or results in inability to continue to comply with study procedures
- Disease progression which requires discontinuation of the study medication or results in inability to continue to comply with study procedures: Effectively the development of

any exclusion criterion.

- Consent withdrawn
- Lost to follow up

The reason for withdrawal will be recorded in the eCRF. If the subject is withdrawn due to an adverse event, the investigator will arrange for follow-up visits or telephone calls until the adverse event has resolved or stabilised, in addition to scheduled visits/contacts.

Imatinib is associated with a number of well-described adverse drug reactions (see Investigators Brochure). Based on these adverse drug reactions possible following treatment with imatinib mesylate, study medication will be discontinued if one or more of the following criteria are met:

- Leukocytes <2.0 x 10^9/L; Thrombocytes <50 x 10^9/L
- AST/ALT: elevation of >10x ULN in case of AST/ALT within reference values at baseline/inclusion or an elevation of >10x baseline in case of elevated AST/ALT at baseline/inclusion; bilirubin: elevation of >3x ULN in case of bilirubin levels within reference values at baseline/inclusion or an elevation of >3x baseline in case of elevated bilirubin levels at baseline/inclusion.
- Occurrence of life-threatening arrythmias, including Torsade-de-Pointe, ventricular fibrillation or ventricular tachycardia.

Subjects in whom study medication is discontinued will remain in the study for further monitoring. In addition to the criteria described above, the investigator/treating physician is allowed to discontinue study medication at their discretion if he/she judges the patient to be negatively affected by study medication. The reason for discontinuation should be recorded in the CRF.

8.6 Replacement of individual subjects after withdrawal

A total of 90 subjects will be included for the full study. Subjects withdrawn for reasons attributed to the study treatment will not be replaced. All other withdrawals (e.g. loss of study subjects due to national/international relocation of subjects following COVID-19 relocation programmes) will be replaced at the discretion of the Sponsor, following discussion with the Investigator.

8.7 Follow-up of subjects withdrawn from treatment

In case a patient or his/her relatives decide to stop the study medication (e.g. due to side effects / non-tolerance), the patient will be asked to stay in the study. In case a patient decides to stay in the study, follow-up will continue according to the study protocol. In case a patient decides to leave the study, follow-up will be discontinued while all collected data are stored.

8.8 Premature termination of the study

Premature termination of the study will take place:

- On instigation of the data safety monitoring board
- On decision of the principal investigator

9. SAFETY REPORTING

9.1 Temporary halt for reasons of subject safety

In accordance to section 10, subsection 4, of the WMO, the sponsor may suspend the study if there are sufficient grounds that continuation of the study will jeopardise subject health or safety. The sponsor will notify the accredited METC without undue delay of a temporary halt including the reason for such an action. The study will be suspended pending a further positive decision by the accredited METC. The investigator will take care that all subjects are kept informed.

9.2 AEs, SAEs and SUSARs

9.2.1 Definitions

Adverse events (AE) are defined as any undesirable experience occurring to a subject during the study, whether or not considered related to the study treatment.

A serious adverse event (SAE) is any untoward medical occurrence or effect that

- results in death;
- is life threatening (at the time of the event);
- Requires in-patient hospitalization or prolongation of existing hospitalization
- results in persistent or significant disability or incapacity;
- Is a congenital abnormality or birth defect;
- Is an important medical event that may jeopardize the subject or may require medical treatment to prevent one of the outcomes listed above.

Adverse reactions are all untoward and unintended responses to an investigational product related to any dose administered. Unexpected adverse reactions are SUSARs if the following three conditions are met:

1. the event must be serious (see §9.2.2);
2. there must be a certain degree of probability that the event is a harmful and an undesirable reaction to the medicinal product under investigation, regardless of the administered dose;
3. the adverse reaction must be unexpected, that is to say, the nature and severity of the adverse reaction are not in agreement with the Reference Safety Information as recorded in the Investigator's Brochure.

9.2.2 Reporting of Adverse Events (AE) and Serious Adverse Events (SAE)

Due to the nature of disease, the incidence of AEs and SAEs, as well as the risk of death due to the underlying condition is high (the hospital mortality in ventilated ICU patients is 21%). In clinical studies conducted in patients with ARDS, changes in vital parameters are frequent, including temporary changes in blood pressure and gas exchange parameters, as are deviations in laboratory values and ECG values.

AE Reporting

Given the high incidence of adverse events inherent to the nature of the underlying condition (i.e., ARDS), given the safety of imatinib as observed in COVID-19 pneumonitis before (COUNTER-COVID study), and given the regular recording of vital signs and blood and ECG parameters as safety indicators in the eCRF, we propose not to report AEs in a standard fashion, except for the following events:

- Pulmonary embolism, as detected on contrast enhance chest CT, not leading to circulatory of pulmonary instability.
- Occurrence of infections, requiring initiation of antibiotic therapy
- Non-life-threatening infusion reactions, including but not limited to skin rash.

SAE Reporting

Given the high incidence of adverse events inherent to the nature of the underlying condition (i.e., ARDS), and given the safety of imatinib as observed in COVID-19 pneumonitis before (COUNTER-COVID study), we propose not to report all serious events as SAEs.

The following SAEs will be reported:

- Death due to any cause
- Cardiopulmonary:
    - The need for extracorporeal membrane oxygenation.
    - Cardiac events like arrythmias requiring CPR or medical resuscitation.
    - Thrombo-embolic events with life-threatening circulatory or pulmonary instability
    - Spontaneous bleeding, requiring blood transfusion or surgical intervention.
    - Myocardial infarction
- Renal: the need for renal replacement therapy
- Hepatic: Liver failure (hepatic SOFA score >4)
- Haematology: Thrombocytopenia (<50^10^9/L), diffuse intravascular coagulation, leukocytopenia (<2^10^9/L), anaemia (haemoglobin <4mmol/L).
- Central Nervous System: Intracranial bleeding or ischemic stroke.
- Life-threatening infusion reactions, requiring intensification of existing intensive care treatment, including additional vasopressor, fluid support, corticosteroids and antihistamines.
- Any unexpected serious event judged as an “untoward medical occurrence”.

If the trial site personnel are unable to complete the electronic SAE form within 24 h after

receiving information about the event, the initial reporting must be done on the paper SAE report and emailed to Simbec-Orion Pharmacovigilance (within 24 h of awareness) at pharmacovigilance@simbecorion.com. The trial site should report the event in the electronic SAE form of the eCRF as soon as possible after that.

A medically qualified person at the trial site identified on the delegation log with this responsibility must assess the SAE. The Principal Investigator or delegated sub-investigators are responsible for the SAE reporting procedures at the site during the trial and must always sign-off on each SAE (regardless of whether reported using the electronic or paper form) even if other site staff have reported the event on behalf of the investigators.

The following events will not be reported as SAEs:

- Cardiopulmonary:
    - Fall in blood pressure, requiring fluid resuscitation or inotropic medication.
    - Respiratory failure, requiring intensification of mechanical ventilation, prone positioning or bronchial intervention.
- Renal:
    - Decrease in eGFR or urine output, not requiring renal replacement therapy

9.2.3 Reporting of Suspected unexpected serious adverse reactions (SUSARs)

Simbec-Orion PV holds the responsibility of submissions to Netherlands Competent Authority (CA) and Central Ethics committee (EC), and will report the following SUSARs to the METC:

- SUSARs that have arisen during this clinical trial that was assessed by the METC;
- SUSARs that have arisen in other clinical trials of the same sponsor and with the same medicinal product, and that could have consequences for the safety of the subjects involved in this clinical trial that was assessed by the METC.

The sponsor will provide expedited reports for all SUSARs to the competent authorities in other Member States, according to the requirements of the Member States.

The expedited reporting will occur not later than 15 calendar days after the sponsor has first knowledge of the adverse reactions. For fatal or life-threatening cases, the term will be maximal 7 calendar days for a preliminary report with another 8 calendar days for completion of the report.

9.2.4 Reporting to Competent Authority and Medical Ethics Committee

Given the high incidence of SAE inherent to the nature of the underlying condition (i.e., ARDS), and given the safety of imatinib in COVID-19 pneumonitis before, we propose to report the SAEs in a line listing every six months to the METC. These will be specified per study arm in the line listing without disclosing the specific arms.

An overview list (line-listing) of all SUSARs will be submitted once every six months to the METC. This line-listing provides an overview of all SUSARs that have occurred since the last update of the Investigator's Brochure. The expedited reporting of SUSARs through the web portal Eudravigilance or ToetsingOnline is sufficient as notification to the competent

authority.

9.3 Annual safety report

In addition to the expedited reporting of SUSARs, the sponsor will submit, once a year throughout the clinical trial, a safety report to the accredited METC and competent authorities of the concerned Member States. This safety report consists of a listing of the following safety indicators specified per study arm without disclosing the specific arms.

- 28-day mortality
- ICU length of stay
- Ventilator free days and alive at Day28.
- Cardiopulmonary: the need for extracorporeal membrane oxygenation.
- Renal: the need for renal replacement therapy
- Hepatic: Liver failure (hepatic SOFA score >4)
- Haematology: Thrombocytopenia (<50^10^9/L), diffuse intravascular coagulation, leukocytopenia (<2^10^9/L), anaemia (haemoglobin <4mmol/L).
- Central Nervous System: Intracranial bleeding or ischemic stroke.

9.4 Data Safety Monitoring Board (DSMB) / Safety Committee

A DSMB will be installed consisting of a chairman, an epidemiologist, and two expert members including a pharmacist (intravenous formulation) and an ICU physician (experience in ARDS). For a full description of the DSMB and its members, we refer to the DSMB charter.

For safety reasons, a safety report will be produced after:

- 10 subjects included (±5/arm) – DSMB meeting 1
- 30 subjects included (±15/arm) – DSMB meeting 2
- 60 subjects included (±30/arm) – DSMB meeting 3

This safety report includes the safety parameters mentioned under §8.1.2, and will be presented to the DSMB.

The advice(s) of the DSMB will only be sent to the Sponsor of the study. Should the Sponsor decide not to fully implement the advice of the DSMB, the sponsor will send the advice to the reviewing METC, including a note to substantiate why (part of) the advice of the DSMB will not be followed.

10. STATISTICAL ANALYSIS

The statistical analysis will be performed using the program SPSS version 22.0 or R. Sample size calculations are presented in §4.4. Continuous data will be tested for normal distribution using a Kolmogorov-Smirnov test. In case of normal distribution, continuous data will be presented as mean ± standard deviation, in case of non-normal distribution, continuous data will be presented as median ± interquartile (IQR) range. Categorical data will be presented as absolute number (%). The continuous variables and the categorical data will be presented quantitatively in tables and in figures. Missing data will be accepted

after maximal efforts to retrieve data.

10.1 Primary study parameter(s)

The primary endpoint is the change in extravascular lung water index (ΔEVLWi) between Day 1 (baseline) and Day 4. The primary endpoint will be represented mean ± standard deviation, in case of non-normal distribution, continuous data will be presented as median ± interquartile (IQR) range, and tested for statistical difference using a t-test or a Mann-Whitney U test in case of non-normal distribution. In addition, ANCOVA analysis will be performed.

10.2 Secondary study parameter(s)

For all parameters below, statistical comparison will be performed between placebo and imatinib group.

Parameter	Presentation	Test
PVPi (Day 1, 2, 4, 7)	Mean±SD	Linear mixed model
Oxygenation index (Day 1, 2, 4, 7, 10 and 28)	Mean±SD	Linear mixed model
PaO2/FiO2 ratio (Day 1, 2, 4, 7, 10 and 28)	Mean±SD	Linear mixed model
Airway driving pressure (Day 1, 2, 4, 7, 10 and 28)	Mean±SD	Linear mixed model
Compliance (Day 1, 2, 4, 7, 10 and 28)	Mean±SD	Linear mixed model
Mechanical power (Day 1, 2, 4, 7, 10 and 28)	Mean±SD	Linear mixed model
SOFA score (Day 1, 2, 4, 7, 10 and 28)	Mean±SD	Linear mixed model
Number of ventilator-free days (Day 1 to 28)	Mean±SD	T-test
Duration of mechanical ventilation (days) (Day 1 to 28)	Mean±SD	T-test
Length of ICU stay (days) (Day 1 to 28)	Mean±SD	T-test
Hospital length of stay (days) (Day 1 to 28)	Mean±SD	T-test
28-day mortality	Number (%)	Cox prop. Hazards
Plasma biomarkers (Day 1, 2, 4, 7, 10)	Median±IQR	MWU / Linear mixed model
Blood cell counts (Day 1, 2, 4, 7 and 10)		
RBC	Mean±SD	Linear mixed model
WBC	Mean±SD	Linear mixed model
Thrombocytes	Mean±SD	Linear mixed model
Kidney function (Day 1, 2, 4, 7 and 10)		
Creatinine	Mean±SD	Linear mixed model
eGFR	Mean±SD	Linear mixed model
Liver enzymes (Day 1, 2, 4, 7 and 10)		
ALT	Mean±SD	Linear mixed model
AST	Mean±SD	Linear mixed model
Bilirubin	Mean±SD	Linear mixed model
γ-glutamyl transferase	Mean±SD	Linear mixed model

Parameter	Presentation	Test
Alkaline phosphatase	Mean±SD	Linear mixed model
NT-proBNP (Day 1, 2, 4, 7 and 10)	Median±IQR	Linear mixed model
SAEs / AE	Number (%)	Descriptive
ECG (ΔQTc time Day 1 versus Day 4)	Mean±SD	T-test
IQR = Interquartile range, MWU = Mann-Whitney U, SD = standard deviation		

Further details on the statistical analyses, including prespecified posthoc stratifications and subgroup analyses are described in the statistical analysis plan.

10.3 Other study parameters

Plasma concentrations of imatinib at C_{trough} will be described by descriptive statistics, including mean, SD, minimum, maximum, and median.

Nonlinear mixed effect modelling as implemented in the NONMEM program (Version 6, Globomax LLC, Hanover, MD, USA) will be used for data-analysis. S-Plus (Version 6.2, Insightful Software, Seattle WA) an R (version 7.2) will be used to visualize the data.

Using a previously validated imatinib PK-model the population pharmacokinetics of imatinib will be characterized in terms of clearance, volume of distribution, and parameters that describe protein binding/ free fraction. Between-patient and, if applicable, within-patient variability in these parameters will be estimated.

Goodness-of-fit of the population PK models will be judged by the goodness-of-fit plots (created by using Pirana and Xpose (version 4.3.2, Niclas Jonsson and Mats Karlsson, Uppsala, Sweden) as well as the precision of the parameter estimates and the magnitude of residual variability. In addition, the objective function value (OFV) will be used to statistically assess the goodness of fit; whether addition of a parameter in the model statistically significantly improves the fit will be determined with the likelihood ratio test. A p-value below 0.05 in a Chi-squared-distribution with one degree of freedom will be considered statistically significant.

Covariate analysis: To explain pharmacokinetic between- and within-patient variability covariates will be tested in a two-step approach. In the first step all different covariates will be introduced to the structural model separately and tested for their significance and improvement of the fit. A p-value of <0.05, determined with the likelihood ratio test (OFV drop of at least 3.8 units), will be considered statistically significant during this step of the analysis. In the second step, all covariates selected during the first step will be included in the model, yielding the intermediate model. A backward elimination procedure (multivariate analysis) will subsequently be used to develop the final model. A covariate will be retained in the model if exclusion results is a statistically significant worsening of the fit, again determined with the likelihood ratio test. A p-value <0.01, corresponding to a OFV increase of at least 6.6 units will be applied for this purpose to correct for the multiple testing phenomenon. Covariates that will be considered include eGFR, creatinine, sex, age, body weight and length (BSA, BMI), comorbidity, concomitant medication, AGP and albumin

levels. The resulting PK model or PK derivatives (AUC C_{max} or $C_{through}$ free/total) will be used for further outcome analysis.

10.4 Interim analysis (if applicable)

There will be no interim analyses on efficacy. Interim analyses on safety will be performed in accordance with the DSMB and will be performed as described under §9.4.

Stopping rules (if applicable):

- DSMB advice based on safety reports.

11. ETHICAL CONSIDERATIONS

11.1 Regulation statement

The general principles of informed consent, ethics review and data management will be in line with good clinical practice (GCP). The study will be conducted according to the principles of the Declaration of Helsinki (2013) and in accordance with the Medical Research Involving Human Subjects Act (WMO).

11.2 Recruitment and consent

11.2.1 Recruitment

Subjects will be recruited at the emergency unit of the AUMC, locations AMC and VUMC, the Onze Lieve Vrouwe Gasthuis, location Oost (Amsterdam), Dijklander Ziekenhuis (Hoorn,Purmerend) and Erasmus MC (Rotterdam). Additional centres may be considered. With a minimum of 5 active centres and an anticipated duration of the trial of 18 months, and a target number of inclusions of 90 subjects, this means that 90/5=18 subjects per centre need to be enrolled, which amounts to <0.25 subjects/centre/week. When considering the AMC and VUMC as representative hospitals, the required rate of inclusion/centre is far below the current rate of presentations/centre.

11.2.2 Deferred consent

For this trial we ask for deferred consent and we appeal to the emergency procedure for consent in medical research as stated in article 6, paragraph 4 of the WMO, similar to a presently running trial of ventilation in a similar patient cohort, the '*REstricted versus Liberal positive end–expiratory pressure in patients without Acute respiratory distress syndrome (RELAx)' – a multicenter randomized controlled trial* (NL60402.018.17).

Pulmonary vascular leak is a phenomenon that is observed early in the course of ARDS. Previously, an early 'exudative' phase (the first 3-5 days in the course of ARDS) was distinguished from a 'reorganising/proliferative' phase (Day 5 and further in the course of disease) [Tomashefski 2000]. Although this distinction is formally abandoned, it is generally accepted that the early course of ARDS is characterised by pulmonary vascular leak and alveolar exudates, in which the pulmonary oedema is the main cause of hypoxemia [Matthay 2012; Huppert 2019]. Based on preclinical and early clinical data there is a reasonable chance that patients will benefit from the vasculoprotective effect of imatinib, especially in the early, exudative phase. The short extent of this exudative phase in time (days) indicates a limited window-of-opportunity to apply therapies directed at preserving

or restoring the alveolocapillary barrier. For this reason, we consider it of utmost importance to initiate the vasculoprotective treatment with imatinib as soon as possible (i.e., within 48 hours after ICU admission), not doing so would largely reduce validity of this trial. Additionally, the potential benefit from the intervention (which would not be available outside of given trial) is likely highest in the early phases.

Subjects admitted to the ICU with ARDS are, without exception, incompetent to give informed consent. Persons who may take the role of legal representative in accordance with the WGBO are: a predefined representative, husband or wife, registered partner or other life partner, a parent or child, brother or sister, and incidentally a curator appointed the judge. However, obtaining informed consent from a legal representative in this situation usually takes much time, even by an experienced research team (see Textbox A). Reasons include the absence of a legal representative within the first 48h after admission, and early after admission to the ICU the legal representatives are far more concerned about the wellbeing of the patient then participation in a trial.

Textbox A – Experiences with deferred consent in critically ill patients

Most critically ill patients who need ventilation cannot be approached for informed consent for a study at ICU admission. Indeed, those patients are usually in severe respiratory distress, sedated or in coma. A prospective observational study on study recruitment practices in critically ill patients performed by a respected and experienced research group in Canada showed that the time from recognizing study eligibility to obtaining informed consent by a legal representative was as high as 12 hours, even while time from recognition to the first contact with a legal representative was as short as 2 hours [Verhaeghe 2005]. A similar procedure was followed in another drug trial in COVID-19 ICU patients that was performed in the Amsterdam UMC, location AMC and VUMC [Vlaar 2020].

The experience of ICU patients enrolled under deferred consent is mainly positive. To investigate contentment of patients that were included using deferred consent, a questionnaire was designed [Jansen 2009] and distributed to participants in the large NICE–SUGAR trial, a trial compared a strict blood glucose control strategy with one that accepts higher blood glucose levels [NICE-SUGAR Study Investigators 2009]. Of the responders (79% of all participants), a large majority (96%) said to have granted consent if they would have been asked. A large majority (93%) mentioned they were happy with the decision made by the representative at the moment they were incapable of giving informed consent [Jansen 2009].

This is in line with our personal experience from the PReVENT trial [PREVENT Investigators 2018], a study that compares two other ventilation strategies. From the PReVENT study we learned that it is very well possible to inform legal representatives about the trial within 24 hours. However due to longer travel distances for some of the legal representatives, obtaining written informed consent was sometimes not possible within the 24 hours: in as many as 19 out of 174 patients (11%) this was a reason for exclusion of the patient. Interestingly, informed consent could have been obtained within 48 hours in all these cases. Therefore, 48 hours is the timeframe that will be used for this study.

For these reasons, we opt for using deferred consent, where informed consent from a legal representative must be obtained as soon as possible, but always within 48 hours after

intubation. While awaiting deferred consent, subjects will undergo screening and first baseline measurements. A PiCCO catheter will be placed (if not already in place), and the baseline EVLW and PCP measurement, as well as thoracic ultrasound in a subpopulation will be performed. The placement of a central venous catheter is part of standard care in intubated subjects. When deferred consent is provided the patient will continue study participation by randomisation and administration of the first IMP dose. When deferred consent is not obtained due to absence of a legal representative in the first 48 hours, or if a legal representative denies participation within the time window of 48 hours, the patient will not be enrolled and screening data will no longer be used. In that case, the PiCCO catheter will be used as arterial line as part of standard care, resulting in no net additional study procedures on top of standard care with the exception of the procedures needed to assess eligibility for the study.

Further consent will be sought from the patient if they recover sufficiently and regain decision-making capacity while in hospital.

11.2.3 No consent in subjects who die before obtaining deferred consent

In case a patient dies before informed consent could be obtained from the legal representative, we propose that the data collected during screening and at baseline will no longer be used. This will be recorded as a screening failure, and the reason for failure will be recorded.

11.2.4 Conclusion deferred consent

Critically ill subjects in need of ventilation are, without exception, incapable to give informed consent at the moment of ICU admission. Patient screening, eligibility checking and placing the PICCO catheter instead of a “normal arterial line” are part of the study procedures but cannot wait until informed consent is obtained from a legal representative. Therefore, a deferred consent procedure is relevant for the current study. Study medication will only be administered after informed consent.

11.2.5 Consent procedure

The recruitment of study subjects is as follows:

<u>1. Approach of subjects (if patient is a candidate for study participation)</u>

a. In cases where a legal representative is present / accessible at ICU admission:
The treating physician asks the legal representative if they would consider participation in a clinical trial for which they may be eligible and obtains their permission, for a member of the study team to contact them

b. If their legal representative is not present / accessible at ICU admission:
The treating physician contacts the study team who initiate the deferred consent procedure. During the deferred consent period, a member of the study team will regularly attempt to contact the patient's legal representative to obtain consent.

<u>2. Informed consent</u>

The investigator gives the patient information and consent statement to the patient's legal representative answering any questions about the study they may have and providing

additional information when requested. The legal representative has at least 6 hours to read and consider whether to provide consent. The informed consent form is signed within 48 hours from ICU admission.
Due to the Covid-19 pandemic, some legal representatives are in isolation. They either have a Covid-19 infection or have to be tested for Covid-19. For this reason, they cannot visit the hospital. If this is the case, the consent procedure will be done by phone and e-mail. The legal representative will first be informed by phone. After the phone call, the legal representative will receive a copy of the participant information sheet, including the informed consent form, by e-mail and will have a reflection period of 6 hours. To confirm participation, the legal representative will sign the informed consent form and send a copy by e-mail. The investigator will sign this copy and return to the legal representative the completed informed consent form containing both signatures. An example of the e-mail is provided in Appendix 3.

11.3 Objection by minors or incapacitated subjects (if applicable)

See §11.2.2.

11.4 Benefits and risks assessment, group relatedness

Despite the devastating consequences of COVID-19 on patients and relatives as well as a huge economic and societal burden on society, there is currently no proven pharmacological treatment that protects the alveolocapillary barrier. The same holds true for subjects with ARDS due to causes other than COVID-19, for which different therapeutic approaches, including statins, β-agonists, steroids and other immunomodulators, have failed to yield any benefit for the ARDS patient over the past 40 years. We propose imatinib as a drug that has a proven protective effect on vascular leak in preclinical and early clinical cases of pulmonary vascular leak. Prevention of development of ARDS (in COVID-19) may have a huge benefit for patient health and for consumption of health resources. There is a longstanding experience with imatinib, and its side effects are considered mild. Whereas most side effects are based on chronic use of imatinib in daily doses of 400-800mg, we propose a dosing scheme of 200mg twice daily (400mg total daily dose) for 7 days.

11.5 Compensation for injury

The sponsor/investigator has a liability insurance which is in accordance with article 7 of the WMO.

The sponsor (also) has an insurance which is in accordance with the legal requirements in the Netherlands (Article 7 WMO). This insurance provides cover for damage to research subjects through injury or death caused by the study. The insurance applies to the damage that becomes apparent during the study or within 4 years after the end of the study.

11.6 Incentives (if applicable)

There are no incentives

12. ADMINISTRATIVE ASPECTS, MONITORING AND PUBLICATION

12.1 Handling and storage of data and documents

The data generated in this study will be encoded, not based on the patient initials and birth-date. A Patient ID code unique to each subject will be used to identify data and the key to the code will be available to the investigators. Furthermore, personal data will comply to the AVG/GDPR. Study data will be stored for the required minimum period of 15 years.

Preservation and registration of human material is performed at the Pulmonary Hypertension biobank. By saving serum and blood plasma at -80˚C, analyses will be performed on biomarkers of metabolism, inflammation, epithelial and endothelial injury, as well as transcriptomics, metabolomics and proteomics. Human material will be stored for 5 years after enrolment and follow-up of the last study subject.

Source Data

Source documents are original documents, data, and records from which subjects' CRF data are obtained. These include, but are not limited to, hospital records (from which medical history and previous and concurrent medication may be summarised into the CRF), clinical and office charts, laboratory and pharmacy records, diaries, microfiches, radiographs, and correspondence.

CRF entries will be considered source data if the CRF is the site of the original recording (i.e., there is no other written or electronic record of data).

A number of data fields in the eCRF (Castor^{edc}) are considered as source documents, in particular the following data fields:

- Smoking
- ECG
- Parts of the in- and exclusion e.g. chronic oxygen use and concomitant use of other medication.
- Adverse events and severity of adverse events. Although the electronic patient file is considered the primary source document, the grading of severity is not always mentioned in the electronic patient file, and therefore subject to interpretation of the study physician.
- Severe adverse events and severity of severe adverse events. Although the electronic patient file is considered the primary source document, the grading of severity is not always mentioned in the electronic patient file, and therefore subject to interpretation of the study physician.

All documents will be stored safely in confidential conditions. On all study-specific documents, other than the signed consent, the subject will be referred to by the study Patient ID code, not by name.

Only trial staff as listed on the Delegation Log shall have access to trial documentation other than the regulatory requirements listed below.

12.2 Monitoring and Quality Assurance

Monitoring will be done by an independent clinical research team of the Clinical Trial

Bureau of the Amsterdam UMC, location VUMC.

12.3 Amendments

A ‘substantial amendment’ is defined as an amendment to the terms of the METC application, or to the protocol or any other supporting documentation, that is likely to affect to a significant degree:

- the safety or physical or mental integrity of the subjects of the trial;
- the scientific value of the trial;
- the conduct or management of the trial; or
- the quality or safety of any intervention used in the trial.

All substantial amendments will be notified to the METC and to the competent authority.
Non-substantial amendments will not be notified to the accredited METC and the competent authority, but will be recorded and filed by the sponsor.

12.4 Annual progress report

The sponsor/investigator will submit a summary of the progress of the trial to the accredited METC once a year. Information will be provided on the date of inclusion of the first subject, numbers of subjects included and numbers of subjects that have completed the trial, serious adverse events/ serious adverse reactions, other problems, and amendments.

12.5 Temporary halt and (prematurely) end of study report

The sponsor will notify the accredited METC and the competent authority of the end of the study within a period of 90 days. The end of the study is defined as the last patient’s last visit.

The sponsor will notify the METC immediately of a temporary halt of the study, including the reason of such an action. In case the study is ended prematurely, the sponsor will notify the accredited METC and the competent authority within 15 days, including the reasons for the premature termination. Within one year after the end of the study, the investigator/sponsor will submit a final study report with the results of the study, including any publications/abstracts of the study, to the accredited METC and the Competent Authority.

12.6 Public disclosure and publication policy

The data generated by this study will be presented at international conferences and published in key journals. Publication will be in accordance with the basic principles of Central Committee on Research Involving Human Subjects (CCMO) statement on publication policy (https://www.ccmo.nl/publicaties/publicaties/2002/03/15/ccmo-notitie-publicatiebeleid).

13. STRUCTURED RISK ANALYSIS

13.1 Potential issues of concern

For a detailed risk analysis, we refer to the Investigators Brochure.

a. Level of knowledge about mechanism of action

The mechanism of action underlying the protective effect of imatinib against pulmonary vascular leak has been clarified by previous preclinical studies from our group. We have demonstrated that imatinib exerts its protective effect by inhibiting Arg/Abl2 a non-receptor tyrosine kinase, which is activated under inflammatory conditions. During inflammation Arg/Abl2 signals to reduce binding of the endothelial cell to the subcellular matrix and to reduce Rac1 activity. Both events contribute to intercellular gap formation and endothelial barrier disruption when the endothelium is exposed to inflammatory mediators. By inhibiting Arg/Abl2 imatinib reinforces the endothelial barrier in inflammatory conditions [Aman 2012]. This mechanism was validated in in vivo models of ARDS/pulmonary vascular leak (Table I), and recent unpublished studies from our group show that Arg/Abl2 knockout mice are protected against vascular leak in a similar fashion as mice treated with imatinib [unpublished data J.Aman].

b. Previous human exposure to the test product(s) and/or products with a similar biological mechanism

We have demonstrated that initiation of imatinib treatment was associated with fast resolution of alveolar oedema in a patient with pulmonary veno-occlusive disease that was treated with imatinib for another indication [Overbeek 2008]. In a second clinical case, a patient was successfully treated with imatinib for capillary leak syndrome. [Aman 2013]. Reports from other groups have also shown that imatinib gave fast resolution of drug-induced pneumonitis [Carnevale-Schianca 2011] or acute interstitial pneumonia [Fenocchio 2016] (both syndromes mimicking ARDS). Recently, a case series was published showing that imatinib effectively reversed pneumonitis in patients suffering from chemotherapy-induced pneumonitis [Landberg 2018]. In 2004, Peng and colleagues have tested the safety and tolerability of intravenous administration of imatinib, which did not yield any safety of tolerability issues.

c. Can the primary or secondary mechanism be induced in animals and/or in *ex-vivo* human cell material?

Yes

d. Selectivity of the mechanism to target tissue in animals and/or human beings

It is currently unknown whether to mechanism of action is selective for the endothelium.

e. Analysis of potential effect

The potential effect of imatinib is based on the protective effect of imatinib on the endothelial barrier, thus preventing pulmonary vascular leak and alveolar flooding during inflammation.

f. Pharmacokinetic considerations

Patients treated with a total daily dose of 400mg/day show imatinib plasma levels ranging between 2-5μM [Sing 2009]. This is supported by preliminary pharmacokinetic analyses in

COVID-19 patients who were treated with imatinib 400mg [Counter-COVID study, interim analysis for 3rd DSMB meeting]. These levels correspond to the protective effect that we have seen on the endothelium in preclinical studies [Aman 2012].

g. Study population
There are no indications that imatinib may have unexpected side effects in this population. We have performed a healthy volunteer study, in which healthy volunteers were subjected to a lung injury model (by lipopolysaccharide inhalation). Treatment of healthy volunteers in this model did not provide any undue effects, and did not impair the immune response [unpublished data].

h. Interaction with other products
see §5.4

i. Predictability of effect
Common side effects of imatinib include flushing, cough, flatulence, gastro-oesophageal reflux, and gastritis. Relevant uncommon side effects include palpitations, cardiac failure, pleural effusion, acute renal failure, melena, chest pain and pancytopenia (although the pancytopenia observed in patients in CML was shown to result from apoptosis of leukemic cells, and that repopulation with non-affected leucocytes was undisturbed under imatinib treatment). In general side effects are mild, and usually occur after chronic use. As the imatinib treatment in this study is relatively short (days) compared to the chronic use in CML (months-years), we anticipate that the side effects observed in CML studies will be less frequent in the study proposed here.

j. Can effects be managed?
All common side effects are generally mild or can be managed. Most of the measurements included in this study protocol involve measurements to monitor safety (cardiac, renal function and liver enzymes). Toxicity is managed by discontinuation of study medication, according to the following rules:

- Leukocytes <2.0 x 10^9/L; Thrombocytes <50 x 10^9/L
- AST/ALT: elevation of >10x ULN in case of AST/ALT within reference values at baseline/inclusion or an elevation of >10x baseline in case of elevated AST/ALT at baseline/inclusion; bilirubin: elevation of >3x ULN in case of bilirubin levels within reference values at baseline/inclusion or an elevation of >3x baseline in case of elevated bilirubin levels at baseline/inclusion.
- Occurrence of life-threatening arrythmias, including Torsade-de-Pointe, ventricular fibrillation or ventricular tachycardia.

13.2 Synthesis

Altogether, this study will test the efficacy of intravenous imatinib on moderate-severe ARDS. The vasculoprotective properties of imatinib suggest that imatinib forms a promising candidate to reduce disease burden and consumption of health care resources in ARDS. The protective effects of imatinib can be obtained at plasma levels observed in patients treated with imatinib for CML, indicating that regular dosing schedules can be used for treatment of ARDS with imatinib. Although side effects of imatinib have been reported to

be mild in CML, the dosing scheme proposed here is much shorter, indicating that side effects may be less common. Safety measures are put in place to monitor potential severe side effects.

APPENDIX 1 SCHEDULE OF ACTIVITIES

**Study Period**	**Screening**	**Baseline**	**Treatment**							**Follow up**	
**Study Day (± Window)**	**0-1[a]**	**1**	**2**	**3**	**4**	**5**	**6**	**7**	**10**	**11-27[b]**	**28[c] (±3)**
ELIGIBILITY											
(Deferred) Informed consent	X										
Demographics	X										
Relevant medical history[d]	X										
SARS-CoV-2 diagnostic test review[e]	X										
Assess ARDS diagnosis and severity[f]	X										
Inclusion and exclusion criteria	X										
STUDY INTERVENTION											
Randomisation		X[g]									
IMP administration		X[g]	X	X	X	X	X	X			
Treatment with SoC	X	X	X	X	X	X	X	X	X	X	X
STUDY PROCEDURES											
12-lead Electrocardiogram (corrected QT interval)	X	X	X		X			X	X		
Height and weight		X							X		
Targeted physical examination[h]		X	X	X	X	X	X	X			
Vital signs: temperature, pulse rate, blood pressure, respiratory rate, SpO_2 and FiO_2	X	X	X	X	X	X	X	X	X		X
Placement of central venous catheter and PiCCO catheter	X										
EVLWi and PVPi		X	X	X	X	X	X	X			
Mechanical ventilation parameters[i]		X	X		X			X	X		X
Arterial blood gas		X	X		X			X	X		
SOFA score and OI[j]		X	X		X			X	X		X
Clinical status[k]		X	X	X	X	X	X	X	X		X
PK sampling		X[l]	X[m]		X[m]			X[m]			
Blood, plasma and serum sampling for biomarker studies		X	X		X			X	X		
Thoracic ultrasound (optional)		X			X						
Targeted medication review (including use of vasopressors)		X	X	X	X	X	X	X	X		X

Study Period	Screening	Baseline	Treatment							Follow up	
**Study Day (± Window)**	**0-1[(a)]**	**1**	**2**	**3**	**4**	**5**	**6**	**7**	**10**	**11-27[(b)]**	**28[(c)] (±3)**
Adverse event evaluation		X	X	X	X	X	X	X	X	X	X
SAFETY LABORATORY											
Haematology, chemistry, liver function tests, coagulation	X[(n)]	X	X		X			X	X[(o)]		
Pregnancy test for females of childbearing potential	X										

EVLWi =Extravascular lung water index; FiO_2=Fraction of inspired oxygen; PaO_2=partial pressure of oxygen; SARS-CoV-2= severe acute respiratory syndrome coronavirus 2; SoC=standard of care; SpO2 = peripheral oxygen saturation; OI = Oxygenation Index PVPi = pulmonary vascular permeability index; SOFA score = Sequential Organ Failure Assessment score.

(a). To be performed prior to randomisation.

(b). Or until hospital discharge, if earlier.

(c). If patient has been discharged before Day 28, this assessment may be conducted by telephone or with a home visit by study staff. For visits conducted by telephone, it will not be possible to perform some scheduled assessments (e.g., ECG). Where patients have discontinued the study prematurely, Day 28 assessments should be performed, where possible.

(d). Medical history includes an estimate of date and time of first signs and symptoms and presence of co-morbidities (e.g., respiratory, cardiovascular, metabolic, malignancy, endocrine, gastrointestinal, immunologic, renal).

(e). Tests performed prior to hospital admission are acceptable provided test result is from a laboratory or validated point of care test.

(f). Documentation of evidence to confirm diagnosis and ARDS severity according to Berlin definition.

(g). Randomisation and first dose of IMP must take place within 48 hours of intubation. IMP should be administered twice daily, 12h (± 2h) apart between 06:00 - 12:00 in the morning and 18:00 - 24:00 in the evening. Exceptions apply on Day 1 – see § 6.2 Dosage and Administration.

(h). Where clinically indicated.

(i). Air driving pressure; respiratory system compliance, PEEP, Tidal volume, and mechanical power.

(j). OI: Assess once daily, in case of more measurements per calendar day, the worst OI, and all related measurements will be taken. Manual techniques should be used only if automated devices are not available.

(k). Record any change in clinical status: change in extubation or reintubation; first unassisted breathing or death; discharge from ICU, hospital or death; WHO ordinal Scale for Clinical Improvement.

(l). Plasma samples for determination of imatinib, albumin and AGP taken at 4h and between 7 and 8h after the start of the first IMP infusion. Where PK sampling cannot be performed on Day 1, samples will be taken 3h and 7h after the start of the first IMP infusion on Day 2 instead. For a subgroup of patients, extra samples will be taken during the IMP infusion and 2h after the start of the first infusion (i.e., at the end of the infusion).

(m). Pre-IMP infusion (am or pm dose).

(n). Laboratory tests performed in the 48 hours prior to first dose of study treatment will be accepted for determination of eligibility. If multiple tests are performed during this time, the test closest to dosing will be regarded as the formal sample to confirm eligibility.

(o). Any laboratory tests performed as part of routine clinical care within ±1 day of Day 10 assessment while hospitalized can be used.

APPENDIX 2 STUDY SAMPLE ANALYSES

The following analyses will be performed as post-hoc analysis on the study material obtained during the study as described in §8.3.

1 – Pharmacokinetics:
Measurement of imatinib total concentration, imatinib free fraction, imatinib metabolite(s) AGP and albumin, where practicable.

2 – Inflammation and coagulation:
Measurement of inflammatory biomarkers and measures of coagulation including, but not limited to, proinflammatory cytokines (e.g., IL-6, IL-8), matrix metalloproteinases, elastase, lactoferrin, proteinase-3, NGAL, D-dimer, protein C, PAI-1.

3 - Markers of endothelial activation and injury:
Measurement of proteins implicated in endothelial cell activation and injury including, but not limited to, angiopoietin-1 and -2, syndecan, endocan, V-CAM, soluble E-selectin, soluble thrombomodulin.

4 – Markers of epithelial injury:
Measurement of proteins implicated in epithelial injury including, but not limited to, surfactant, protein D, soluble RAGE, soluble Fas (ligand).

5 – Omic studies:
Transcriptomics (RNA sequencing) and proteomics will be performed to identify molecular signatures that relate to disease outcome and treatment response. No DNA is taken and no genetic studies will be performed.

APPENDIX 3 EXAMPLE EMAIL FOR CONSENT OF LEGAL REPRESENTATIVE

Geachte mevrouw/meneer,

Zojuist hebben we via de telefoon met u gesproken over deelname van uw partner of familielid aan wetenschappelijk onderzoek. Uw partner of familielid is opgenomen op de Intensive Care door een COVID-19 infectie. De behandelend arts was van mening dat de infectie dringend behandeld moest worden om de ademhaling te verbeteren. Uw partner of familielid heeft hiervoor de standaard behandeling gekregen. Inmiddels bent u door de behandelend arts over de medische toestand van uw partner of familielid op de hoogte gesteld.

Op de Intensive Care wordt wetenschappelijk onderzoek gedaan om de behandeling van Covid-19 te verbeteren. Immers, een deel van de patiënten overlijdt of moet langdurig beademd worden. Zoals we per telefoon al hebben besproken, willen we vragen of u toestemming geeft dat uw partner of familielid meedoet aan een wetenschappelijk onderzoek.

Dit onderzoek wordt uitgevoerd om te bepalen of het medicijn imatinib uw partner of familielid helpt om sneller te herstellen van de Covid-19 infectie. Eerder onderzoek heeft laten zien dat imatinib in tablet vorm veilig is voor patiënten die op de zaal of de Intensive care zijn opgenomen met Covid-19. Dit onderzoek heeft ook laten zien dat patiënten die behandeld worden met imatinib minder lang beademd hoeven worden en een lagere kans op overlijden hebben. Het innemen van tabletten is moeilijk in patiënten die beademd worden. In het huidige onderzoek willen we daarom kijken of imatinib via infuus het herstel van Covid-19 versnelt. De effecten van imatinib vergelijken we met de effecten van een placebo. Een placebo is een stof zonder actieve stof, een 'nepmedicijn'.

Tot op heden werden 193 ernstige COVID-19 patiënten met longontsteking behandeld met imatinib. Bij deze patiënten werd aangetoond dat het veilig is en goed wordt verdragen. Uw partner of familielid kan rechtstreeks baat hebben bij deelname aan dit onderzoek. Het is echter ook mogelijk dat uw partner of familielid te maken krijgt met bijwerkingen, die hij of zij niet zou hebben gehad als hij of zij niet behandeld was met imatinib.

Met behulp van loting wordt bepaald of bij uw partner/familielid het medicijn imatinib of de placebo (nepmedicijn) via infuus wordt toegediend. De onderzoeker weet niet of uw partner of familielid het medicijn imatinib of placebo krijgt. In beide gevallen krijgt uw partner of familielid de standaard behandeling.

Omdat uw partner/familielid nu niet zelf kan beslissen over deelname vragen wij nu aan u om te bevestigen dat we het onderzoek mogen uitvoeren. In een later stadium zullen we uitgebreidere toelichting geven aan uw partner of familielid en uzelf. **Zou u ons willen laten weten of u er mee akkoord gaat dat uw partner/familielid deelneemt aan dit onderzoek?**

Wij wensen u veel sterkte in deze moeilijke tijd. Bij vragen kunt u altijd contact met ons opnemen.

Met vriendelijke groet,

Dear Sir / Madam,

We contacted you by phone about the participation of your partner or family member in scientific research. Your partner or family member has been admitted to the Intensive Care Unit because of a COVID-19 infection. The treating physician deemed urgent treatment to improve breathing necessary. Your partner or family member has received standard treatment for this. You have been informed by the attending physician about the medical condition of your partner or family member.

In the Intensive Care Unit scientific research is being done to improve the treatment of Covid-19. Despite standard treatments, some of the patients die or require mechanical ventilation for a long time. As we have already discussed over the phone, we would like to ask if you consent to your partner or family member taking part in a scientific study.

This study is performed to determine if the drug imatinib is helping your partner or family member recover from the Covid-19 infection more quickly. Previous research has shown that imatinib in tablet form is safe for patients admitted to the ward or intensive care unit with Covid-19. This study also showed that patients treated with imatinib need less ventilation and have a lower risk of death. In patients who are ventilated, administration of tablets is difficult. In the current study, we therefore want to test whether imatinib via intravenous drip accelerates the recovery of Covid-19. We compare the effects of imatinib with the effects of a placebo. A placebo is a substance without an active substance, a 'fake medicine'.

To date, 193 severe COVID-19 pneumonia patients have been treated with imatinib. It has been shown to be safe and well tolerated in these patients. Your partner or family member may directly benefit from participating in this study. However, your partner or family member may also experience side effects that he or she would not have had if he or she had not been treated with imatinib.

By chance it will be determined whether your partner / family member will be given the drug imatinib or the placebo (dummy drug) by intravenous drip. The investigator does not know whether your partner or family member is receiving the drug imatinib or placebo. In both cases, your partner or family member will receive standard treatment.

Because your partner / family member cannot now decide for himself whether to participate, we now ask you to confirm that we are allowed to carry out the research. At a later stage we will provide more detailed information to your partner or family member and yourself.

Would you please let us know if you agree that your partner / relative will participate in this study?

We wish you all the best in this difficult time. If you have any questions, you can always contact us.

Best regards,

APPENDIX 4 PROTOCOL AMENDMENT HISTORY

Protocol Version 3.1 (dated 16 April 2021 replaces protocol version 2.2, dated 22 January 2021 approved by METc VUmc 22 January 2021).

The amendment incorporates the following main changes:

- Clarification of blood sampling times for Pharmacokinetics on Day 1, 2, 4 and 7.
- Removal of optional ultrasound assessment on Day 7.
- Amended statistical testing of the secondary endpoint Number of ventilator-free days (Day 1 to 28) from a linear mixed modelling analysis to a T-test
- Inclusion of the option to obtain consent by email from legal representatives isolating due to COVID-19
- Extend EVLWi and PVPi measurements from Day 1,2,4,7 to Day 1-7.
- Extend QTc measurements from Day 1,2,4,7 to Day 1-7.
- Clarification on concomitant medication data to be recorded in the CRF.

Protocol version 4.1, dated 24th Feb 2022 (replaces protocol version 3.1, dated 16 April 2021 approved by METc VUmc 26 April 2021)

The amendment incorporates the following main changes:

- A general update of the background information in the protocol, incorporating the outcomes of the COUNTER COVID study.
- An adaptation of the study protocol, adding 3 exclusion criteria, and adding COVID vaccination status to the baseline characteristics that are recorded.
- Adaptation of the study protocol with regard to the planning of the ECGs. The previous protocol stated at some places that ECGs were performed on days 1-7 and 10, while elsewhere it was stated that ECGs were performed on days 1,2,4,7,10. We have aligned this throughout the protocol to day 1,2,4,7,10.
- An additional phrase in §4.4 allows for a blinded sample size re-estimation in case patient recruitment falls to an unacceptably low level.
- Relaxation of medication administration times from 06.00-10.00 to 06.00-12.00 and 18.00-22.00 to 18.00-24.00.
- The local PI of the VUMC was changed from prof.dr. L. Heunks to Dr. P.R. Tuinman.

14. REFERENCES

Aman J, van Bezu J, Damanafshan A, et al. Effective treatment of edema and endothelial barrier dysfunction with imatinib. Circulation. 2012;126(23):2728-2738.

Aman J, Duijvelaar E, Botros L, et al. Imatinib in patients with severe COVID-19: a randomised, double-blind, placebo-controlled, clinical trial. Lancet Respir Med. 2021 Sep;9(9):957-968. doi: 10.1016/S2213-2600(21)00237-X. Epub 2021 Jun 18. Erratum in: Lancet Respir Med. 2021 Aug;9(8):e84.

Acute Respiratory Distress Syndrome Network, Brower RG, Matthay MA, Morris A, Schoenfeld D, Thompson BT, Wheeler A. Ventilation with lower tidal volumes as compared with traditional tidal volumes for acute lung injury and the acute respiratory distress syndrome. N Engl J Med. 2000 May 4;342(18):1301-8.

Bartelink IH, Bet PM, Widmer N, et al. Elevated acute phase proteins affect pharmacokinetics in COVID-19 trials: Lessons from the CounterCOVID - imatinib study. CPT Pharmacometrics Syst Pharmacol. 2021 Dec;10(12):1497-1511. doi: 10.1002/psp4.12718. Epub 2021 Oct 24.

Beigel JH, Tomashek KM, Dodd LE, et al; ACTT-1 Study Group Members. Remdesivir for the Treatment of Covid-19 - Preliminary Report. N Engl J Med. 2020 May 22. Epub ahead of print. PMID: 32445440.

Bernard GR, Luce JM, Sprung CL, et al. High-dose corticosteroids in patients with the adult respiratory distress syndrome. N Engl J Med. 1987;317:1565–1570.

Blackwood B, Ringrow S, Clarke M, et al. A Core Outcome Set for Critical Care Ventilation Trials. Crit Care Med. 2019;47(10):1324-1331.

Breccia M, Abruzzese E, Bocchia M, Bonifacio M, Castagnetti F, Fava C, Galimberti S, Gozzini A, Gugliotta G, Iurlo A, Latagliata R, Luciano L, Pregno P, Rege-Cambrin G, Rosti G, Stagno F, Tiribelli M, Foà R, Saglio G; Campus CML working group. Chronic myeloid leukemia management at the time of the COVID-19 pandemic in Italy. A campus CML survey. Leukemia. 2020 Aug;34(8):2260-2261.

Cao B, Wang Y, Wen D, et al. A Trial of Lopinavir-Ritonavir in Adults Hospitalized with Severe Covid-19 [published online ahead of print, 2020 Mar 18]. N Engl J Med. 2020;10.1056/NEJMoa2001282.

Chislock EM, Pendergast AM. Abl family kinases regulate endothelial barrier function in vitro and in mice. PLoS One. 2013;8:e85231.

Coleman CM, Sisk JM, Mingo RM, Nelson EA, White JM, Frieman MB. Abelson Kinase Inhibitors Are Potent Inhibitors of Severe Acute Respiratory Syndrome Coronavirus and Middle East Respiratory Syndrome Coronavirus Fusion. J Virol. 2016 Sep 12;90(19):8924-33.

Craig TR, Duffy MJ, Shyamsundar M, McDowell C, O'Kane CM, Elborn JS, McAuley DF. A randomized clinical trial of hydroxymethylglutaryl- coenzyme a reductase inhibition for acute lung injury (The HARP Study). Am J Respir Crit Care Med. 2011 Mar 1;183(5):620-6.

Deininger MW, Goldman JM, Lydon N, Melo JV. The tyrosine kinase inhibitor CGP57148B selectively inhibits the growth of BCR-ABL-positive cells. Blood. 1997;90(9):3691–3698.

Combes A, Hajage D, Capellier G, et al; EOLIA Trial Group, REVA, and ECMONet. Extracorporeal Membrane Oxygenation for Severe Acute Respiratory Distress Syndrome. N Engl J Med. 2018 May 24;378(21):1965-1975.

Fenocchio E, Depetris I, Campanella D, et al. Successful treatment of gemcitabine-induced acute interstitial pneumonia with imatinib mesilate: a case report. BMC Cancer. 2016;16(1):793.

Ferguson, N.D., Cook, D.J., Guyatt, G.H., Mehta, S., Hand, L., Austin, P., et al. High-Frequency Oscillation in Early Acute Respiratory Distress Syndrome. N Engl J Med. 2013; 368: 795–805.

Foà R, Bonifacio M, Chiaretti S, Curti A, Candoni A, Fava C, Ciccone M, Pizzolo G, Ferrara F. Philadelphia-positive acute lymphoblastic leukaemia (ALL) in Italy during the COVID-19 pandemic: a Campus ALL study. Br J Haematol. 2020 Jul;190(1):e3-e5.

García M, Cooper A, Shi W, et al. Productive replication of Ebola virus is regulated by the c-Abl1 tyrosine kinase. Sci Transl Med. 2012;4(123):123ra24.

Guérin C, Reignier J, Richard JC, Beuret P, Gacouin A, Boulain T, Mercier E, Badet M, Mercat A, Baudin O, Clavel M, Chatellier D, Jaber S, Rosselli S, Mancebo J, Sirodot M, Hilbert G, Bengler C, Richecoeur J, Gainnier M, Bayle F, Bourdin G, Leray V, Girard R, Baboi L, Ayzac L; PROSEVA Study Group. Prone positioning in severe acute respiratory distress syndrome. N Engl J Med. 2013 Jun 6;368(23):2159-68.

Han Y, Yang L, Duan X, et al. Identification of Candidate COVID-19 Therapeutics using hPSC-derived Lung Organoids. bioRxiv [Preprint]. 2020 May 5:2020.05.05.079095.

Huppert LA, Matthay MA, Ware LB. Pathogenesis of Acute Respiratory Distress Syndrome. Semin Respir Crit Care Med. 2019 Feb;40(1):31-39.

Jansen TC, Kompanje EJO, Bakker J. Deferred proxy consent in emergency critical care research: ethically valid and practically feasible. Crit Care Med. 2009;37(1 Suppl):S65-S68.

Kaneko T, Kawamura Y, Maekawa T, et al; PiCCO Pulmonary Edema Study Group. Global end-diastolic volume is an important contributor to increased extravascular lung water in patients with acute lung injury and acuterespiratory distress syndrome: a multicenter observational study. J Intensive Care. 2014 Apr 1;2(1):25.

Kim IK, Rhee CK, Yeo CD, Kang HH, Lee DG, Lee SH, Kim JW. Effect of tyrosine kinase inhibitors, imatinib and nilotinib, in murine lipopolysaccharide-induced acute lung injury during neutropenia recovery. Crit Care. 2013 Jun 20;17(3):R114.

Kurimoto, N., Nan, Y.-S., Chen, Z.-Y., Feng, G.-G., Komatsu, T., Kandatsu, N., et al. (2004). Effects of specific signal transduction inhibitors on increased permeability across rat endothelial monolayers induced by neuropeptide Y or VEGF. American Journal of Physiology - Heart and Circulatory Physiology 287: H100–H106.

Langberg MK, Berglund-Nord C, Cohn-Cedermark G, Haugnes HS, Tandstad T, Langberg CW. Imatinib may reduce chemotherapy-induced pneumonitis. A report on four cases from the SWENOTECA. Acta Oncol. 2018;57(10):1401–1406.

Letsiou, E., Rizzo, A.N., Sammani, S., Naureckas, P., Jacobson, J.R., Garcia, J.G.N., et al. Differential and opposing effects of imatinib on LPS- and ventilator-induced lung injury. Am. J. Physiol. Lung Cell Mol. Physiol. 2015;308: L259–269.

Maggio R, Peragine N, De Propris MS, et al. Immunocompetent cell functions in Ph+ acute lymphoblastic leukemia patients on prolonged Imatinib maintenance treatment. Cancer Immunol Immunother. 2011;60(4):599–607.

Matthay MA, Ware LB, Zimmerman GA. The acute respiratory distress syndrome. J Clin Invest. 2012;122:2731-40.

Matthay MA, McAuley DF, Ware LB. Clinical trials in acute respiratory distress syndrome: challenges and opportunities. Lancet Respir Med. 2017 Jun;5(6):524-534.

Morales-Ortega A, Bernal-Bello D, Llarena-Barroso C, et al. Imatinib for COVID-19: A case report. Clin Immunol. 2020 Sep;218:108518.

Mumprecht S, Matter M, Pavelic V, Ochsenbein AF. Imatinib mesilate selectively impairs expansion of memory cytotoxic T cells without affecting the control of primary viral infections. Blood. 2006 Nov 15;108(10):3406-13.

NICE-SUGAR Study Investigators, Finfer S, Chittock DR, Su SY, et al. Intensive versus conventional glucose

control in critically ill patients. N Engl J Med. 2009 Mar 26;360(13):1283-97.

Overbeek MJ, van Nieuw Amerongen GP, Boonstra A, Smit EF, Vonk-Noordegraaf A. Possible role of imatinib in clinical pulmonary veno-occlusive disease. Eur Respir J. 2008;32(1):232–235.

Papazian L, Forel JM, Gacouin A, et al; ACURASYS Study Investigators. Neuromuscular blockers in early acute respiratory distress syndrome. N Engl J Med. 2010 Sep 16;363(12):1107-16.

Peng B, Dutreix C, Mehring G, Hayes MJ, Ben-Am M, Seiberling M, Pokorny R, Capdeville R, Lloyd P. Absolute bioavailability of imatinib (Glivec) orally versus intravenous infusion. J Clin Pharmacol. 2004a Feb;44(2):158-62.

Perkins GD, McAuley DF, Thickett DR, Gao F. The beta-agonist lung injury trial (BALTI): a randomized placebo-controlled clinical trial. Am J Respir Crit Care Med. 2006 Feb 1;173(3):281-7.

Peter JV, John P, Graham PL, Moran JL, George IA, Bersten A. Corticosteroids in the prevention and treatment of acute respiratory distress syndrome (ARDS) in adults: meta-analysis. BMJ. 2008;336:1006–1009.

Writing Group for the PReVENT Investigators, Simonis FD, Serpa Neto A, Binnekade JM, et al. Effect of a Low vs Intermediate Tidal Volume Strategy on Ventilator-Free Days in Intensive Care Unit Patients Without ARDS: A Randomized Clinical Trial. JAMA. 2018 Nov 13;320(18):1872-1880.

RECOVERY Collaborative Group, Horby P, Lim WS, Emberson JR, et al. Dexamethasone in Hospitalized Patients with Covid-19 - Preliminary Report. N Engl J Med. 2020 Jul 17. Epub ahead of print. PMID: 32678530.

Reeves PM, Bommarius B, Lebeis S, McNulty S, Christensen J, Swimm A, Chahroudi A, Chavan R, Feinberg MB, Veach D, Bornmann W, Sherman M, Kalman D. Disabling poxvirus pathogenesis by inhibition of Abl-family tyrosine kinases. Nat Med. 2005 Jul;11(7):731-9. Epub 2005 Jun 26. Erratum in: Nat Med. 2005 Dec;11(12):1361.

Rhee, C.K., Lee, S.H., Yoon, H.K., Kim, S.C., Lee, S.Y., Kwon, S.S., et al. Effect of Nilotinib on Bleomycin-Induced Acute Lung Injury and Pulmonary Fibrosis in Mice. Respiration 2011;82:273–287.

Rizzo AN, Aman J, van Nieuw Amerongen GP, Dudek SM. Targeting Abl kinases to regulate vascular leak during sepsis and acute respiratory distress syndrome. Arterioscler Thromb Vasc Biol. 2015;35(5):1071–1079.

Schmidt M, Hajage D, Lebreton G, et al.; Groupe de Recherche Clinique en REanimation et Soins intensifs du Patient en Insuffisance Respiratoire aiguE (GRC-RESPIRE) Sorbonne Université; Paris-Sorbonne ECMO-COVID investigators. Extracorporeal membrane oxygenation for severe acute respiratory distress syndrome associated with COVID-19: a retrospective cohort study. Lancet Respir Med. 2020 Nov;8(11):1121-1131.

Shi H, Han X, Jiang N, et al. Radiological findings from 81 patients with COVID-19 pneumonia in Wuhan, China: a descriptive study [published online ahead of print, 2020 Feb 24]. Lancet Infect Dis. 2020;S1473-3099(20)30086-4.

Singh N, Kumar L, Meena R, Velpandian T. Drug monitoring of imatinib levels in patients undergoing therapy for chronic myeloid leukaemia: comparing plasma levels of responders and non-responders. Eur J Clin Pharmacol. 2009;65:545–549.

Steinberg KP, Hudson LD, Goodman RB, et al. Efficacy and safety of corticosteroids for persistent acute respiratory distress syndrome. N Engl J Med. 2006;354:1671–1684.

Stephens RS, Johnston L, Servinsky L, Kim BS, Damarla M. The tyrosine kinase inhibitor imatinib prevents lung injury and death after intravenous LPS in mice. Physiol Rep. 2015;3. pii: e12589.

Tian S, Hu W, Niu L, Liu H, Xu H, Xiao SY. Pulmonary Pathology of Early-Phase 2019 Novel Coronavirus (COVID-19) Pneumonia in Two Patients With Lung Cancer [published online ahead of print, 2020 Feb 28]. J Thorac Oncol. 2020;S1556-0864(20)30132-5.

Tomashefski JF Jr. Pulmonary pathology of acute respiratory distress syndrome. Clin Chest Med. 2000 Sep;21(3):435-66.

Verhaeghe S, Defloor T, Van Zuuren F, Duijnstee M, Grypdonck M. The needs and experiences of family members of adult patients in an intensive care unit: a review of the literature. Journal of Clinical Nursing. 2005;14(4):501-509.

Vlaar APJ, de Bruin S, Busch M, et al. Anti-C5a antibody IFX-1 (vilobelimab) treatment versus best supportive care for patients with severe COVID-19 (PANAMO): an exploratory, open-label, phase 2 randomised controlled trial. Lancet Rheumatol. 2020 Sep 28.

National Heart, Lung, and Blood Institute Acute Respiratory Distress Syndrome (ARDS) Clinical Trials Network, Wiedemann HP, Wheeler AP, Bernard GR, Thompson BT, Hayden D, deBoisblanc B, Connors AF Jr, Hite RD, Harabin AL. Comparison of two fluid-management strategies in acute lung injury. N Engl J Med. 2006 Jun 15;354(24):2564-75.

Writing Group for the PReVENT Investigators, Simonis FD, Serpa Neto A, Binnekade JM, et al. Effect of a Low vs Intermediate Tidal Volume Strategy on Ventilator-Free Days in Intensive Care Unit Patients Without ARDS: A Randomized Clinical Trial. JAMA. 2018 Nov 13;320(18):1872-1880.

Wu Z, McGoogan JM. Characteristics of and Important Lessons From the Coronavirus Disease 2019 (COVID-19) Outbreak in China: Summary of a Report of 72 314 Cases From the Chinese Center for Disease Control and Prevention [published online ahead of print, 2020 Feb 24]. JAMA. 2020;10.

Xu Z, Shi L, Wang Y, et al. Pathological findings of COVID-19 associated with acute respiratory distress syndrome [published online ahead of print, 2020 Feb 18] [published correction appears in Lancet Respir Med. 2020 Feb 25;:]. Lancet Respir Med. 2020;S2213-2600(20)30076-X.

Young, D., Lamb, S.E., Shah, S., MacKenzie, I., Tunnicliffe, W., Lall, R., et al. High-Frequency Oscillation for Acute Respiratory Distress Syndrome. N Engl J Med. 2013; 368: 806–813.

Zhou F, Yu T, Du R, et al. Clinical course and risk factors for mortality of adult inpatients with COVID-19 in Wuhan, China: a retrospective cohort study [published online ahead of print, 2020 Mar 11] [published correction appears in Lancet. 2020 Mar 12;:]. Lancet. 2020;S0140-6736(20)30566-3.
