# Supplementary material for: Efficacy and safety of intravenous imatinib in COVID-19 ARDS: a randomized, double-blind, placebo-controlled clinical trial
Source: Crit Care. 2023 Jun 8;27:226. doi: 10.1186/s13054-023-04516-4 (PMC10249575; doi:10.1186/s13054-023-04516-4)
Supplement: Supplementary file 3 — Additional file 3. Statistical Analysis Plan. [file 13054_2023_4516_MOESM3_ESM.pdf]

Statistical Analysis Plan

2020-005447-23

A randomised, double-blind, placebo-controlled study to investigate the safety and efficacy of intravenous imatinib mesylate (Impentri®) in subjects with Acute Respiratory Distress Syndrome induced by COVID-19

Prepared for:

Amsterdam UMC

Subsidised by the European Union

Prepared by:

Simbec Orion Clinical Services Ltd
Merthyr Tydfil Industrial Park
Cardiff Road
Merthyr Tydfil
CF48 4DR
United Kingdom

CONFIDENTIAL

**Version:** Final 1.0	**Author**
**Date:** 24May2022	R Morton

Statistical Analysis Plan

2020-005447-23

A randomised, double-blind, placebo-controlled study to investigate the safety and efficacy of intravenous imatinib mesylate (Impentri®) in subjects with Acute Respiratory Distress Syndrome induced by COVID-19

Author: R Morton

Version: Final 1.0

The undersigned have reviewed and revised this SAP and find it to be consistent with the study requirements:

DocuSigned by:
Richard Morton
Signer Name: Richard Morton
Signing Reason: I approve this document
Signing Time: 24-May-2022 | 17:37 BST
[illegible]425C849F940546C5FB03

Richard Morton, Head of Statistics, Simbec-Orion Group Ltd.	Date

DocuSigned by:
christelle grall
Signer Name: christelle grall
Signing Reason: I approve this document
Signing Time: 24-May-2022 | 19:48 BST
DD4B2DF65A6241C195A2ABDAC81AE309

Christelle Grall, Project Director on behalf of Alexia Flamarion, Project Manager, Simbec-Orion Clinical Services	Date

DocuSigned by:
James McGinlay
Signer Name: james McGinlay
Signing Reason: I approve this document
Signing Time: 25-May-2022 | 09:25 BST
D4E896BE86BE4398A10248CE7C0804EB

James McGinlay, Senior Technical Writing Associate, Simbec-Orion Clinical Services	Date

DocuSigned by:
Jurjan Aman
Naam Ondertekenaar: Jurjan Aman
Reden voor ondertekening: Ik keur dit document goed
Ondertekentijd: 24-mei-2022 | 19:09 BST
3BE720AB11614B42B0789CDBD0B82B36

Dr Jurjan Aman, Coordinating Investigator, VUmc representative	Date

TABLE OF CONTENTS

1 INTRODUCTION 9
1.1 General 9
1.2 Changes from Protocol 9
2 STUDY OBJECTIVES 9
3 STUDY DESIGN 10
3.1 Overview 10
3.2 Inclusion and Exclusion Criteria 10
3.3 Study Treatment 10
3.4 Study Timepoints 10
3.5 Sample Size Considerations 11
3.6 Randomisation 11
4 STUDY VARIABLES AND COVARIATES 11
4.1 Primary Variable 11
4.2 Secondary Efficacy Variables 11
4.3 Pharmacokinetic Variables 12
4.4 Pharmacodynamic Variables 12
4.5 Other Outcome Variables 12
4.6 Safety Variables 13
5 DEFINITIONS 14
6 ANALYSIS SETS 14
6.1 Intent to Treat Set 14
6.2 Safety Set 15
6.3 Per Protocol Set 15
6.4 PK Analysis Set (PKS) 15
6.5 PD Analysis Set (PDS) 15
7 SAFETY MONITORING 15
8 INTERIM ANALYSES 15
9 DATA 16
9.1 eCRF Data 16
9.2 External Data 16
9.2.1 Laboratory Data 16
9.2.2 Other non-CRF data 16
9.3 Randomisation list 16

9.4 Programming and Data Review 16
10 STATISTICAL METHODS 17
10.1 General Principles 17
10.2 Stratification and Covariate Adjustment 18
10.3 Interactions 18
10.4 Missing Data 18
10.5 Pooling of Sites 18
10.6 Multiplicity 18
10.7 Subgroup Analyses 19
10.8 Statistical Issues 19
11 STATISTICAL OUTPUT 19
11.1 Patient Disposition 19
11.2 Patient Characteristics at Baseline 19
11.2.1 Demographic and Baseline Characteristics 19
11.2.2 Medical History and Current Medical Conditions 20
11.3 Efficacy Analyses 21
11.3.1 Primary Variable 21
11.3.2 Secondary Variables 21
11.4 PK Analyses 24
11.5 PD analyses 24
11.6 Safety Analyses 24
11.6.1 Adverse Events 24
11.6.2 Laboratory Data 26
11.6.3 Vital Signs 26
11.6.4 Electrocardiogram 27
11.7 Study Drug Exposure and Compliance 27
11.8 Prior and Concomitant Medication 28
11.9 Ventilation Parameters 28
11.10 SOFA Score 29
11.11 PICCO 29
12 LITERATURE CITATIONS/REFERENCES 29
13 LIST OF TABLES, FIGURES AND LISTINGS 30
13.1 List of Tables 30
13.2 List of Figures 33
13.3 List of Listings 33
14 SHELLS FOR TABLES, FIGURES AND LISTINGS 35
15 APPENDICES 89
15.1 Study flowchart and/or schedule 89

GLOSSARY OF ABBREVIATIONS

%CV	Coefficient of Variation
AE	Adverse Event
ALT	Alanine Transaminase (also SGPT)
AM	Arithmetic Mean
ANCOVA	Analysis of Covariance
ANOVA	Analysis of Variance
AST	Aspartate Transaminase (also SGOT)
ATC	Anatomical Therapeutic Chemical
AUC	Area Under the Plasma Concentration Curve
AUCinf	Area Under the Plasma Concentration-time Curve calculated from the time of dosing to infinity
AUCt	Area Under the Plasma Concentration-time Curve calculated from the time of dosing to the last measurable concentration
AUCtau	Area Under the Plasma Concentration-time Curve calculated over one dosing interval at steady state
BMI	Body Mass Index
CDISC	Clinical Data Interchange Standards Consortium
CI	Confidence Interval
Cmax	Maximum observed plasma concentration
Cmaxss	Maximum observed plasma concentration over one dosing interval at steady state
Cminss	Minimum observed plasma concentration over one dosing interval at steady state
CR	Complete Response
CRF	Case Report Form
CRO	Clinical Research Organisation
CS	Clinically Significant
CSR	Clinical Study Report
CTCAE	Common Toxicity Criteria Adverse Event
CV	Coefficient of Variation
DHM	Data Handling Manual
DMP	Data Management Plan
DOB	Date of Birth

DRM	Data Review Meeting
DSMB	Data Safety Monitoring Board
ECG	Electrocardiogram
GM	Geometric Mean
ICH	International Conference on Harmonisation
IDMC	Independent Data Monitoring Committee
IMP	Investigational Medicinal Product
IQR	Interquartile Range
ITT	Intent to Treat Set
IV	Intravenous
LambdaZ	Terminal phase rate constant
LOCF	Last Observation Carried Forward
LS Mean	Least Squares Mean
MedDRA	Medical Dictionary for Regulatory Activities
Mg	Milligram
Ml	Millilitre
MMRM	Mixed Models Repeated Measures
N	Number of Patients
N	Number of Events
NCS	Not clinically significant
Od	Once daily
OFV	Objective Function Value
PD	Pharmacodynamics
PDS	Pharmacodynamics Set
PK	Pharmacokinetics
PKS	Pharmacokinetics Set
PPS	Per Protocol Set
PR	Partial Response
PT	Preferred Term
q12h	Every 12 hours
QC	Quality Control
QoL	Quality of Life
RA	Accumulation ratio
RBC	Red Blood Cell
SAF	Safety Set
SAP	Statistical Analysis Plan

SAS	Statistical Analysis System
SD	Standard Deviation
SDTM	Study Data Tabulation Model
SOC	System Organ Class
$t_{1/2}Z$	Terminal phase half life
TEAE	Treatment Emergent Adverse Event
tmax	Time from dosing to the maximum observed plasma concentration
tmaxss	Time from dosing to the maximum observed plasma concentration over one dosing interval at steady state
VAS	Visual Analogue Scale
VFD_{Surv}	Number of days free of mechanical ventilation and survival
WBC	White Blood Cell
WHO	World Health Organisation
WHODD	World Health Organisation Drug Dictionary
µg	Microgram

1 INTRODUCTION

1.1 General

This statistical analysis plan (SAP) describes the statistical methods to be used during the reporting and analyses of data collected under Amsterdam UMC Protocol 2020-005447-23 and should be read in conjunction with the study protocol and case report form (CRF).

This version of the plan has been developed using the protocol Version 4.1 dated 24/02/2022 and CRF/ Electronic Data Capture (EDC) specifications dated 08/04/2021. Any further changes to the protocol or CRF will be reviewed for potential impact on the SAP which will be amended if it is deemed necessary.

At the time of writing this version of the SAP, recruitment stopped in March 2022.

Draft 1 was reviewed by the Simbec-Orion project manager, medical writer and statistical reviewer. The analysis plan will be finalised and approved by the sponsor prior to database lock.

1.2 Changes from Protocol

The protocol states that a physical examination will be conducted at Baseline and at Day 1-10 where clinically indicated; however, this is not present in the CRF, thus no analysis will be undertaken within this SAP of the physical examinations.

2 STUDY OBJECTIVES

Primary:

• Efficacy: To evaluate the effect of intravenous imatinib compared to standard of care on limiting development of extravascular lung water in invasively mechanically ventilated subjects with COVID-19-related ARDS.

Secondary:

• Efficacy: To evaluate the effect of intravenous imatinib compared to standard of care on patient outcomes in mechanically ventilated subjects with COVID-19-related ARDS.

• Safety: To evaluate the safety and tolerability of intravenous imatinib compared to standard of care in mechanically ventilated subjects with COVID-19-related ARDS

• Pharmacokinetics: To determine imatinib pharmacokinetics in subjects with COVID-19-related ARDS.

3 STUDY DESIGN

3.1 Overview

This is a randomised, double-blind, parallel-group, placebo-controlled, multi-centre clinical study comparing intravenous imatinib mesylate with placebo in invasively mechanically ventilated subjects with COVID-19-related ARDS. The study plans to enrol up to 90 subjects (45 subjects/treatment arm; see sample size calculation). In this two-arm study, eligible subjects will be randomly allocated to receive imatinib mesylate or placebo in a 1:1 ratio. Besides the randomisation to treatment of placebo, all subjects will be cared for using local treatment protocols as standard of care. At each of the participating centres, standard treatment protocols may include: COVID-19-specific medication (e.g. tociluzimab/sarilumab, anti-COVID antibodies and dexamethasone), low tidal volume ventilation, conservative fluid management and prone positioning in case of persistently low PaO2/FiO2. All subjects (and/or their LARs) in this study have consented for their data to be used by the sponsor for the protocol-defined research and publication purposes, and therefore, all subjects' data will be included in the analyses as per this SAP. There is provision in the study for subjects and/or their LARs to opt out of the further use of this data by Exvastat as a commercial entity, whose intent is to use their data as part of a regulatory submission for purposes of market registration. If any subject opts out, their data will not be used by Exvastat for market registration purposes.

3.2 Inclusion and Exclusion Criteria

To be eligible for inclusion into this study, each patient must fulfil all inclusion criteria and not violate any exclusion criteria (for the protocol under which they are entered) during screening prior to randomisation. Details of the inclusion and exclusion criteria are presented in the protocol and amendments.

3.3 Study Treatment

A total of 90 patients will be recruited sequentially to receive the following doses:

- Imatinib 200 mg b.i.d. (administered as an 8 mg/mL solution for i.v. infusion) or
- Placebo (identical volume of solution for i.v. infusion [0.01M acetate buffer with 1.9% glycerol])

for 7 days, or until patient is discharged from the ICU.

3.4 Study Timepoints

There is a Screening period of 48 hours and a Follow-up period of 17 days. Visits and visit windows will be as follows:

Visit	Day	Window
Screening to Day 10 inc. has no window allocation		
Day 11-27	11-27 Follow Up	or hospital discharge if earlier
Day 28	28 Follow Up	± 3 days

If more than one visit occurs within a window, the nearest to the scheduled time will be presented within the summaries.

See Section 15.1 for the Study Schedule.

3.5 Sample Size Considerations

Sample size calculations were done with the formula: $z\alpha/2 - z\pi = (n/2)1/2 * (\mu 1 - \mu 2)/\sigma$, in which $z\alpha/2\text{-}z\pi$ = 2,8 (α = 5% en π = 80%). The change (Δ) in EVLWi between Day 1and Day 4 is the primary outcome. The baseline EVLWi is expected to be around 17ml/kg, as previously described for patients with moderate-severe ARDS [Kushimoto 2013; Kaneko 2014]. The placebo group is expected to have a ΔEVLW at Day 1 and Day 4 to be 0.5 (µ1), based on previously literature from collaborating groups [Perkins 2006; Craig 2011]; in the imatinib group the ΔEVLWi to be -4 (µ2). This is considered a clinically relevant difference as this difference was found to independently predict ARDS mortality in another clinical study [Brown, Ann Intens Care 2013]. The expected treatment effect is based on the imatinib effect observed in preclinical data: 25% reduction in vascular leak [Aman 2012]. The sigma (σ) for ΔEVLWi is set at 7.0, based on previous EVLWi studies [Craig 2011; Kaneko 2014]. Using the equation above, this yields 76 subjects with 38 subjects/arm. Considering a drop-out rate of 15%, a total of 90 subjects will be recruited. A blinded sample size re-estimation may be performed if patient recruitment falls to an unacceptably low level. The blinded sample size re-estimation will be done by reestimating the sigma (σ) based on the baseline EVLWi measurements done in patients already included in the study.

3.6 Randomisation

Subjects will undergo 1:1 randomisation to receive intravenous imatinib (200mg bid) or placebo for 7 days. When deferred consent is provided the patient will continue study participation by randomisation and administration of the first IMP dose. When deferred consent is not obtained due to absence of a legal representative in the first 48 hours, or if a legal representative denies participation within the time window of 48 hours, the patient will not be enrolled, and screening data will no longer be used.

Randomisation will occur on day 1.

4 STUDY VARIABLES AND COVARIATES

4.1 Primary Variable

The primary variable for statistical comparison between treatment groups will be:

- change in extravascular lung water index (EVLWi) between baseline (Day 1) and Day 4.

4.2 Secondary Efficacy Variables

The following secondary efficacy variables will be analysed:

Pulmonary oedema, gas-exchange and respiratory mechanics:

- EVLWi (Day 1, 2, 4 and 7)

- Pulmonary vascular permeability index (PVPi: Day 1, 2, 4 and 7)
- Oxygenation index (OI: Day 1, 2, 4, 7, 10 and Day 28, if available)
- PaO2/FiO2 ratio (Day 1, 2, 4, 7, 10 and Day 28, if available)
- Airway driving pressure (Day 1, 2, 4, 7, 10 and Day 28, if available)
- Compliance of the respiratory system (Day 1, 2, 4, 7, 10 and Day 28, if available)
- Mechanical power (Day 1, 2, 4, 7, 10 and Day 28, if available)

Organ function and outcome:

- Sequential Organ Failure Assessment (SOFA) score (Day 1, 2, 4, 7, 10 and Day 28, if available);
- Number of ventilator-free days and alive at Day 28 (VFD_{surv}).

  Death will be assigned a score of 0. A sensitivity analysis where death is assigned a score of -1 will be conducted.

  Extubation will be defined at having been extubated for at least 48 hours.

- Duration of mechanical ventilation (days) between Day 1 and 28.
- Length of ICU stay (days) between Day 1 and 28.
- Hospital length of stay (days) between Day 1 and 28.
- 28-day mortality.
- 9-point WHO ordinal Scale for Clinical Improvement

4.3 Pharmacokinetic Variables

Pharmacokinetics will be measured via serum levels of:

- Day 1: Imatinib, albumin and AGP plasma levels at 4h and between 7h and 8h after start of IMP infusion. If for logistical reasons PK sampling cannot be performed at these times on Day 1, PK samples should be collected 3h and 7h after the first IMP infusion on Day 2 instead.
- Day 1 for a subgroup of patients: Imatinib, albumin and AGP plasma levels during IMP infusion and 2h after the start of the infusion (i.e., at the end of the infusion).
- Day, 2, 4 and 7: Imatinib, albumin and AGP plasma levels pre-IMP infusion (am or pm dose).

The PK analysis will be performed as a post-hoc analysis.

4.4 Pharmacodynamic Variables

The following pharmacodynamic variables will be measured:

- Thoracic ultrasound
- Thoracic ultrasound will be performed at Day 1 and 4 in a sub-group of patients.

4.5 Other Outcome Variables

- It is hypothesised that there may be an interaction between baseline albumin/bicarbonate levels and the degree at which imatinib has a treatment effect. The EVLWi/VFDsurv versus albumin/bicarbonate levels assessed as continuous variables will be presented graphically with those variables defined as outlined below.

Parameter	Presentation	Test
ΔEVLW (Day 1 to 4)	Individual data points per patient	Albumin level at Day 0 assessed as a continuous variable within ITT analysis set. Comparison of placebo and imatinib groups.
VFDsurv	Individual data points per patient	Albumin level at Day 0 assessed as a continuous variable within ITT analysis set. Comparison of placebo and imatinib groups.
ΔEVLW (Day 1 to 4)	Individual data points per patient	Bicarbonate level at Day 0 assessed as a continuous variable within ITT analysis set. Comparison of placebo and imatinib groups.
VFDsurv	Individual data points per patient	Bicarbonate level at Day 0 assessed as a continuous variable within ITT analysis set. Comparison of placebo and imatinib groups.

As the cutoff levels cannot be determined prior to the final SAP version being signed these are to be defined as ad-hoc analyses.

Graphical representations of change in EVLW vs Albumin or Bicarbonate will be presented with each treatment group summarised separately on the same plot.

- Baseline demographics (age, sex, intoxications);
- Medical history and comorbidity;
- Vital parameters;
- Use of other drugs
- COVID-19 vaccination status

4.6 Safety Variables

Safety will be evaluated by the following:

- Blood cell count, i.e., haemoglobin, thrombocytes and leucocytes (Day 1, 2, 4, 7 and 10).
- Kidney function, estimated glomerular filtration rate, sodium and potassium (Day 1, 2, 4, 7 and 10).
- Liver enzymes, i.e., AST, ALT, Alkaline Phosphatase, γ-glutamyl transferase, bilirubin (Day 1, 2, 4, 7 and 10).
- NT-proBNP (Day 1, 2, 4, 7 and 10).
- SAEs / AE.
- Corrected QT interval at ECG (Day 1, 2, 4, 7 and 10).

5 DEFINITIONS

Study Drug: Study drug is taken to mean either Imatinib or placebo.

Baseline: Baseline is defined by patient and by variable as the last non-missing not on treatment value (including repeat and unscheduled) before the first dose of study drug. This will normally be the pre-dose assessment on Day 1 but if this assessment is missing (or not planned) then the assessment at the Screening visit will be used instead if available.

Study Day: Study day is the number of days since start of treatment where the date of first dose is counted as Day 1.

Treatment Exposure: Treatment exposure is the number of days during the treatment period that the patient was exposed to the study treatment and is calculated as:

(Date_of_last_dose) – (Date_of_first_dose) + 1

Compliance: Compliance is the number of doses actually taken divided by the scheduled number of doses expressed as a percentage that is taken on trial, before death or early withdrawal.

Non-compliance: Non-compliance is defined as taking less than 80% or more than 120% of study medication that is taken on trial, before death or early withdrawal.

Protocol Deviation: a deviation related to study inclusion or exclusion criteria, conduct of the trial, patient management or patient assessment. This refers to any change, divergence, or departure from the study design or procedures defined in the protocol. Deviations recorded by the Project Manager or CRA, or detected by Data Management or by Statistical programming checks as described in Section 6.3 will be identified and discussed at the Data Review Meeting before database lock to agree which should be included in Listing 16.2.2.

Evaluable: Evaluable is defined in the protocol and is a guide to the need for continued recruitment. The term is not used in the statistical context, but is approximately equivalent to the set of patients with a result for the primary efficacy endpoint.

6 ANALYSIS SETS

Membership of the analysis sets will be reviewed and agreed at a Blind Data Review Meeting (BDRM) before database lock.

6.1 Intent to Treat Set

The Intent to Treat (ITT) is defined as those patients who are randomised and provide post-baseline efficacy data.

The ITT will be used for all efficacy analyses. Patients who receive the wrong treatment in error will be analysed according to the treatment received.

6.2 Safety Set

The Safety Set (SAF) is defined as all patients who are randomised and receive at least one dose of investigational drug.

The Safety Set will be used for all safety analyses.

6.3 Per Protocol Set

The Per-Protocol Set (PP) is defined as all patients in the ITT who had ≤1 of protocolized IMP doses missing during Day 1-4 of the study period. Patients with no PICCO data at Baseline (Day 1) and Day 4 will also be excluded. Any exclusions from the PP must be agreed and signed prior to DBL.

Baseline data and the primary analysis will be repeated using the PP Set. If either the numbers in ITT/PP are identical or if it is agreed at the time of the BDRM meeting that the difference between the populations is sufficiently negligible, tables using the PP set will not be produced.

6.4 PK Analysis Set (PKS)

PK analysis is not part of the current analysis, and will be performed in a prespecified PK/PD post-hoc analysis

6.5 PD Analysis Set (PDS)

PD analysis is not part of the current analysis, and will be performed in a prespecified PK/PD post-hoc analysis.

7 SAFETY MONITORING

There will be three DSMB meetings where data will be supplied masked as specified in the DSMB Charter.

Programs to generate outputs, will be undertaken blinded, once reviewed and QCed the programs will be transferred to a unblinded restricted area and rerun. Outputs will be sent only to those respondents indicated in the DSMB Charter.

8 INTERIM ANALYSES

There will be no interim analyses on efficacy. Interim analyses on safety will be performed in accordance with the DSMB.

9 DATA

9.1 eCRF Data

CRF data will be provided by Simbec-Orion data management to the statistics department as SAS data sets in Simbec-Orion standard format which will be used for programming the outputs to be included in the CSR. Populated data sets will be available when programming starts. These may contain dummy data if real data is not yet available.

9.2 External Data

9.2.1 Laboratory Data

Cumulative monthly transfers of local lab data will be received as ASCII files in an agreed format and uploaded to a SAS dataset by Simbec-Orion Data Management. Populated test transfers (using dummy data if necessary) will be received before programming can start. The following results will be included:

- Safety Haematology: i.e., haemoglobin, thrombocytes and leucocytes
- Safety Biochemistry: Creatinine, estimated glomerular filtration rate, sodium and potassium, AST (SGOT), ALT (SGPT), Alkaline Phosphatase, γ-glutamyl transferase, bilirubin, NTproBNP.
- Biomarker analysis is not part of the current analysis, and will be performed in a prespecified post-hoc biomarker study
- Serum pregnancy test

9.2.2 Other non-CRF data

PK data will be analysed by VUmc/vendor.

9.3 Randomisation List

The randomisation list will be uploaded to a SAS dataset following database lock.

9.4 Programming and Data Review

Programming of analysis datasets, tables, figures and listings will be ongoing during the data management of the study. Outputs for the DSMB will be reviewed, but not fully QCd. Blinding information for the DSMB will be masked.

When the final data is considered clean, key listings (to be agreed) will be run and distributed to the study team for review. A blind data review meeting will be held to discuss the outcome of this review, the imputations for the primary endpoint and the protocol deviations. Once all data issues have been resolved and the analysis populations approved, the database will be locked. The final run of outputs and quality control (QC) will then take place.

10 STATISTICAL METHODS

10.1 GENERAL PRINCIPLES

All statistical methods will be based on the International Conference on Harmonisation (ICH) E9 document “Statistical Principles for Clinical Trials”.

Data will be summarised by treatment group. A total column showing all patients will be included for baseline and safety summaries. Where appropriate, data will also be summarised by visit with summaries for each visit attended as scheduled and an additional summary for final (last scheduled visit or early withdrawal). The format of the summaries is defined in the shells at the end of this document.

In summary and analysis tables of continuous variables, standard descriptive statistics (N, mean, standard deviation [SD], median, Inter Quartile Range (IQR) minimum and maximum) will be presented. Least squares mean (LS mean), standard error (SE) and 95% confidence interval (CI) will be presented in the statistical analysis outputs as appropriate. For non-parametric analysis median and IQR will be presented as appropriate. The minimum and maximum statistics will be presented in summary tables to the same number of significant figures as the original data. The mean/AM, median, IQR, LS mean, GM, CI, SD and SE will be presented to one more significant than the original data.

For numeric data which includes non-numeric values (e.g., PK data reported as BLQ or lab results reported as < 10 or >100) the following principles will be applied when summarising the data:

- BLQ will be replaced with a value that is ½ of the lower limit of quantification (LLQ)
- Results reported as < x will be treated in the same way as BLQ with LLQ=x
- Otherwise, AM, GM, SD, CI and %CV will not be calculated
- Whenever meaningful, minimum, median and maximum will be presented based on the reported data (e.g., minimum = <10, median = 20, maximum = >100)

In summary tables of categorical variables, the number of non-missing observations by category will be presented with percentages. The number of missing observations will also be presented when non-zero. Unless otherwise specified, the denominator for each percentage will be the number of non-missing observations within the column. All percentages will be presented to one decimal place.

Patient line plots will use actual time on the time axis.

If changes in severity for the same TEAE have been reported separately but with the same AE number, they will be collapsed to a single AE with maximum severity for the summary tables but listed as reported.

Classifications of medical history, concomitant medication and adverse events will be sorted alphabetically within the summary tables.

If any laboratory assessments are repeated at the same visit, the result from the repeat assessment will be used in summaries. Both values will be listed.

CRF data collected/ Data collected on the eCRF will be presented within data listings. The data listings will be sorted by treatment group, country, centre number, patient number and visit/day. Treatment group will be as allocated (randomised). If any patients receive the wrong treatment this will be flagged in all listings. Visits outside the visit windows will be identified within the listings. For patients who

complete the study, the last visit will be described in the listings as Day 28; for those who withdraw early it will be described as 'End of Study'.

The date format for all output presentations will be 'ddMMMyyyy'.

All statistical analysis will be performed using SAS 9.4 / SAS Enterprise Guide 8.3 or higher, or R.

All hypothesis testing will be carried out at the 5% (2-sided) significance level unless stated otherwise.

P-values will be rounded to four decimal places. P-values less than 0.0001 will be reported as <0.0001 in tables.

Continuous data will be tested for normal distribution using Shapiro-Wilk test and observation of the QQ plot. For the case of normal distribution, continuous data will be presented as mean ± standard deviation, for a non-normal distribution, continuous data will be presented as median, interquartile range (IQR). Categorical data will be presented as absolute number (%).

If any of the assumptions underlying the formal statistical methods proposed are violated during the analysis of the final data, alternative statistical methods will be used and any changes documented in the statistical methods section of the clinical study report (CSR), including the rationale for use.

10.2 Stratification and Covariate Adjustment

The randomisation will take place via Castor using variable block sizes with stratification per participating centre. The primary analysis will be adjusted for in case of baseline imbalances (approximately >5% difference).

10.3 Interactions

No analyses of interactions are planned.

10.4 Missing Data

For the primary endpoint, in case of missing data at baseline, the first EVLWi value obtained will carried backward (but no later than from Day 2), for other missing EVLWi values, the last measurement will taken forward (but no earlier than from Day 3). As a sensitivity analysis the primary endpoint analysis will be repeated without data imputation. For other outcomes, there will be no imputation of missing data in this study.

10.5 Pooling of Sites

Sites will be pooled for all analyses. Centres will be treated as stratified.

10.6 Multiplicity

After testing for the primary endpoint, the key secondary endpoints will be considered. The primary endpoint and the key secondary endpoints will thus be tested in a sequential order Table 11.3.2.1. This step-down testing procedure, which obeys the closed testing principle, strongly controls the overall type I error at the 0.05 two-sided level.

For other endpoints, no adjustment for multiplicity will be proposed.

10.7 Subgroup Analyses

Not Applicable

10.8 Statistical Issues

Not Applicable

11 STATISTICAL OUTPUT

General principles for layout of the statistical output are described in Section 10.1, including specification of the table columns, and these are illustrated for each unique table in the table shells in Section 15. For clarity and brevity in this document the phrase "by treatment group" is understood for all summaries and is not included within the text of this section.

11.1 Patient Disposition

Information about screening failures will be provided by the project manager and presented in the CSR as a brief summary of the most frequent reasons for failure.

The patient disposition table will summarise the following data for all randomised patients:

- The number (%) of patients in the ITT
- The number (%) of patients in the SS
- The number (%) of patients in the PK Set
- The number (%) of patients in the PD Set
- The number (%) of patients in the PP Set
- The number (%) of patients who completed the study

The patient disposition table as described above will also be presented by centre.

A table showing the following variables will be generated:

A summary of reasons for study conclusion (number (%)) of patients by treatment and overall will be outputted.

The number (%) of patients who completed Treatment and the reasons for discontinuing treatment will be summarised.

Days since hospital admission, and Days since ICU admission, Days Alive from inclusion, Days since Intubation, estimated number of days since onset of symptoms, Days since positive SARS CoV-2 test descriptive statistics by treatment will be outputted.

A listing of all patients with protocol deviations will be presented. A data listing presenting the eligibility for the analysis sets for each patient will also be presented. A Listing of Patient Status will be generated.

11.2 Patient Characteristics at Baseline

11.2.1 Demographic and Baseline Characteristics

Demographics

Descriptive statistics for age at time of consent will be tabulated.

BMI is the patient's body weight in kilograms divided by the square of the patient's height in metres.

Age, gender, height (m), weight(kg) and BMI (kg/m^2), Admission Type (Medical, Planned Surgical and Emergency surgical) will be summarised using the FAS.

Baseline - General

A Table showing predicted body weights, and the difference of weight upon inclusion and predicted weight.

Social History, Smoking details and Alcohol consumption details will be listed only.

Baseline – Admission Condition

The estimated number of days since the onset of symptoms and day since positive SARS CoV-2 Test will be displayed using descriptive statistics by treatment, non -covid-19 infection, non -Covid-19 pneumonia, Pancreatitis, Shock, High-risk trauma, High-risk, surgery will be tabulated by category number and (%) by treatment.

All admission Conditions will be listed.

Baseline QTc and labs will be represented in section 11.6.5 and 11.6.2 respectively and listed with ECG and labs.

11.2.2 Medical History and Current Medical Conditions

The number (%) of patients (Yes, No and Total) reporting each condition will be presented using the FAS. For Diabetes mellitus, Solid Malignancy and Liver disease the following categories will be outputted.

Variable	Categories	Note
Diabetes mellitus	Total<br>None/Diet Controlled<br>Yes	Denominator is number in column<br>Yes = sum of Uncomplicated and End-Organ damage
	Uncomplicated<br>End-Organ damage	Denominator is the number of Yes's
Solid Malignancy	Total<br>No<br>Yes	Denominator is number in column<br>Yes= sum of Localized and Metastastic
	Localized<br>Metastastic	Denominator is the number of Yes's
Liver Disease	Total<br>No<br>Yes	Denominator is number in column<br>Yes = sum of mild and moderate/severe

	Mild<br>Moderate/Severe	Denominator is the number of Yes's

All Medical History data will be listed.

11.3 Efficacy Analyses

Treatment comparisons will be treatment vs, placebo. The main analysis set for the efficacy analyses will be the FAS. The primary analysis will be repeated for the PP Set.

11.3.1 Primary Variable

The primary endpoint is the change in extravascular lung water index (ΔEVLWi) between Day 1 (baseline) and Day 4. The primary endpoint will be represented as mean ± standard deviation, in case of non-normal distribution, continuous data will be presented as median, interquartile range (IQR) and tested for statistical difference using a t-test or a Mood test in case of non-normal distribution. In addition, ANCOVA analysis will be performed.

The primary endpoint analysis below will be double programmed by Simbec-Orion using SAS Enterprise Guide 8.3.

- The difference in EVLW between day 1 and day 4 assessed by a T-test.
- A figure is drawn summarizing the individual trajectories over the first week and boxplots per group per timepoint.

11.3.2 Secondary Variables

All statistical comparison will be performed between placebo and imatinib group. Where applicable Section 4.7 for Covariate analysis will be followed and stratification by Centre.

Table 11.3.2.1 Key and Exploratory Secondary Variables and Order of Analysis

Parameter	Presentation	Test
**Key Secondary Endpoints**		
PaO2/FiO2 ratio (Day 1, 2, 4, 7, 10 and 28)	Mean±SD	Summarised by visit using descriptive statistics and analysed using a MMRM model stratifying for possible baseline imbalances. The treatment effect (LS Means by treatment group and difference in LS Means) will be estimated at each visit with the associated 95% CI and p-value.
Number of ventilator-free days (Day 1 to 28)	Median±IQR	Wilcoxon rank-sum test (Mann Whitney U-test) will be performed.
Length of ICU stay (days) (Day 1 to 28)	Mean±SD	Two Sample, T-test will be performed.
28-day mortality	Number (%)	The summary statistics N and % will be produced. Proportional Hazards Cox model including the same terms as those fitted in the primary endpoint analysis. The Hazard ratio for the treatment effect with the associated 95% CI and p-value will be provided. Kaplan-Meier curves will be presented.<br>The log-rank test, stratified by Centre will be performed

Exploratory Secondary Endpoints		
PVPi (Day 1, 2, 4, 7)	Mean±SD	Summarised by visit using descriptive statistics and analysed using a MMRM model stratifying for possible baseline imbalances. The treatment effect (LS Means by treatment group and difference in LS Means) will be estimated at each visit with the associated 95% CI and p-value.
Oxygenation index (Day 1, 2, 4, 7, 10 and 28)	Mean±SD	Summarised by visit using descriptive statistics and analysed using a MMRM model stratifying for possible baseline imbalances. The treatment effect (LS Means by treatment group and difference in LS Means) will be estimated at each visit with the associated 95% CI and p-value.
Airway driving pressure (Day 1, 2, 4, 7, 10 and 28)	Mean±SD	Summarised by visit using descriptive statistics and analysed using a MMRM model stratifying for possible baseline imbalances. The treatment effect (LS Means by treatment group and difference in LS Means) will be estimated at each visit with the associated 95% CI and p-value.
Compliance (Day 1, 2, 4, 7, 10 and 28)	Mean±SD	Summarised by visit using descriptive statistics and analysed using a MMRM model stratifying for possible baseline imbalances. The treatment effect (LS Means by treatment group and difference in LS Means) will be estimated at each visit with the associated 95% CI and p-value.
Mechanical power (Day 1, 2, 4, 7, 10 and 28)	Mean±SD	Summarised by visit using descriptive statistics and analysed using a MMRM model stratifying for possible baseline imbalances. The treatment effect (LS Means by treatment group and difference in LS Means) will be estimated at each visit with the associated 95% CI and p-value.
Total SOFA score (Day 1, 2, 4, 7, 10 and 28)	Mean±SD	Summarised by visit using descriptive statistics and analysed using a MMRM model stratifying for possible baseline imbalances. The treatment effect (LS Means by treatment group and difference in LS Means) will be estimated at each visit with the associated 95% CI and p-value.
Duration of mechanical ventilation (days) (Day 1 to 28)	Mean±SD	Two Sample, T-test will be performed.
Hospital length of stay (days) (Day 1 to 28)	Mean±SD	Two Sample, T-test will be performed.
**Safety Endpoints**		
Blood cell counts (Day 1, 2, 4, 7 and 10)		
RBC	Mean±SD	Summarised by visit using descriptive statistics and analysed using a MMRM model stratifying for possible baseline imbalances. The treatment effect (LS Means by treatment group and difference in LS Means) will be estimated at each visit with the associated 95% CI and p-value.
WBC	Mean±SD	Summarised by visit using descriptive statistics and analysed using a MMRM model stratifying for possible baseline imbalances. The treatment effect (LS Means by treatment group and difference in LS Means) will be estimated at each visit with the associated 95% CI and p-value.
Thrombocytes	Mean±SD	Summarised by visit using descriptive statistics and analysed using a MMRM model stratifying for possible baseline imbalances. The

		treatment effect (LS Means by treatment group and difference in LS Means) will be estimated at each visit with the associated 95% CI and p-value.
Kidney function (Day 1, 2, 4, 7 and 10)		
Creatinine	Mean±SD	Summarised by visit using descriptive statistics and analysed using a MMRM model stratifying for possible baseline imbalances. The treatment effect (LS Means by treatment group and difference in LS Means) will be estimated at each visit with the associated 95% CI and p-value.
eGFR	Mean±SD	Summarised by visit using descriptive statistics and analysed using a MMRM model stratifying for possible baseline imbalances. The treatment effect (LS Means by treatment group and difference in LS Means) will be estimated at each visit with the associated 95% CI and p-value.
Liver enzymes (Day 1, 2, 4, 7 and 10)		
ALT	Mean±SD	Summarised by visit using descriptive statistics and analysed using a MMRM model stratifying for possible baseline imbalances. The treatment effect (LS Means by treatment group and difference in LS Means) will be estimated at each visit with the associated 95% CI and p-value.
AST	Mean±SD	Summarised by visit using descriptive statistics and analysed using a MMRM model stratifying for possible baseline imbalances. The treatment effect (LS Means by treatment group and difference in LS Means) will be estimated at each visit with the associated 95% CI and p-value.
Bilirubin	Mean±SD	Summarised by visit using descriptive statistics and analysed using a MMRM model stratifying for possible baseline imbalances. The treatment effect (LS Means by treatment group and difference in LS Means) will be estimated at each visit with the associated 95% CI and p-value.
y-glutamyl transferase	Mean±SD	Summarised by visit using descriptive statistics and analysed using a MMRM model stratifying for possible baseline imbalances. The treatment effect (LS Means by treatment group and difference in LS Means) will be estimated at each visit with the associated 95% CI and p-value.
Alkaline phosphotase	Mean±SD	Summarised by visit using descriptive statistics and analysed using a MMRM model stratifying for possible baseline imbalances. The treatment effect (LS Means by treatment group and difference in LS Means) will be estimated at each visit with the associated 95% CI and p-value.
NT-proBNP (Day 1, 2, 4, 7 and 10)	Median, IQR	Summarised by visit using descriptive statistics and analysed using a MMRM model stratifying for possible baseline imbalances. The treatment effect (LS Means by treatment group and difference in LS Means) will be estimated at each visit with the associated 95% CI and p-value.
SAEs/ AE	Number (%)	Descriptive number and percent by SOC and PT for each treatment and overall
ECG (ΔQTc time Day 1 versus Day 4)	Mean±SD	Two Sample, T-test will be performed.

11.4 PK Analyses

PK analysis is not part of the current analysis, and will be performed in a prespecified PK/PD post-hoc analysis

11.5 PD analyses

PD analysis is not part of the current analysis, and will be performed in a prespecified PK/PD post-hoc analysis

11.6 Safety Analyses

11.6.1 Adverse Events

All adverse events (AE) will be classified using the version of the MedDRA coding dictionary specified in the DHM.

Adverse events (AE) are defined on the CRF as

- Pulmonary embolism, as detected a contrast enhanced chest CT, not leading to circulatory arrest.
- Infections requiring initiation of antimicrobial therapy
- Non-life-threatening infusion reactions, including but not limited to rash

occurring to a subject during the study, whether or not considered related to the study treatment.

Serious adverse event (SAE) is defined on the CRF as

- The need for initiation of extracorporeal life support
- Life-threatening arrythmias requiring cardiopulmonary or medical resuscitation
- Thrombo-embolic events leading to life-threatening circulatory or pulmonary instability
- Spontaneous bleeding requiring blood transfusions or surgical intervention
- Myocardial infarction
- The need for initiation of renal replacement therapy
- Liver failure (bilirubin >204 micromol/L)
- Hemoglobin <4 mmol/L
- Thrombocytopenia <50 × 10^9/L
- Leukopenia <2 x 10^9/L
- Serum hemoglobin <4.0 mmol/L
- Intracranial bleeding or ischemic stroke
- Death of any cause
- Life-threatening infusion reactions, requiring intensification of existing threatment, including additional vasopressor use, fluid resuscitation, corticosteroids or antihistamines
- Other AE: any unexpected serious event judged as an untoward medical occurrence

SUSARs: adverse reactions what are all untoward and unintended responses to an investigational product related to any dose administered. These unexpected adverse reactions are SUSARs if the following 3 conditions are met:

- the event must be serious.
- there must be a certain degree of probability that the event is a harmful and an undesirable reaction to the medicinal product under investigation, regardless of the administered dose.
- the adverse reaction must be unexpected, that is to say, the nature and severity of the adverse reaction are not in agreement with the Reference Safety Information as recorded in the Investigator's Brochure.

Drug-Related: all possibly, probably, and definitely related treatment-emergent adverse events

Not Related: Not Related and Unlikely related treatment-emergent adverse events

Events will be classified as treatment-emergent if they started or increased in severity on or after the first date of medication dosing at Day 1 and up to study closure or withdrawal date. The Status of each patient at 8:00 am is asked, there for excluding Day 1(Start of Medication Dosing to 8 am Day 2), all AE days will be 8:00 am to the proceeding 8:00 am. i.e Day 2 will be Day 2 8:00 am to Day 3 8:00 am.

Treatment-emergent adverse events (TEAEs) will be further classified as follows:

***Severe TEAEs**:* Severity classified as 'severe', 'Life-threatening' and 'Death' or missing.

Serious TEAEs: Serious classified as 'yes' or missing.

Drug-related TEAEs: Relationship to study drug classified as 'possible', 'probable' or 'definite' or missing.

***Serious drug-related TEAEs**:* Both serious and drug-related, as specified above.

TEAEs leading to withdrawal from study: Action taken classified as 'discontinued'.

TEAEs overall and in each of the above classifications will be summarised.

Summaries for each level of severity (Mild, Moderate, Severe, Life-threatening and Death) will also be presented.

Summaries by AE Type will also be presented for treatment-emergent events. Similar tables will be presented for each of the classifications of treatment-emergent events above.

All AE summary tables will show the number (%) of patients having at least one event and the number of events in each treatment group and overall. Note: If a patient has sequential AE types these will be summarised once within the count for N (%) of patients, but each event will be counted as a day within the number of reports n of each AE. Changes in severity of the same AE (if collected) will be counted only once within the number of reports n of each AE.

The following will be presented in listing format within the data summaries:

- Deaths
- Serious Adverse Events
- Adverse Events which Led to Withdrawal

All adverse events (including non-treatment-emergent events) recorded on the CRF will be listed within the data listings.

11.6.2 Laboratory Data

Routine clinical laboratory tests will be carried out at Day 1, 2, 4, 7 and 10.

Parameters: - Blood cell count, i.e., haemoglobin, thrombocytes and leucocytes

- Kidney function, Creatinine, estimated glomerular filtration rate, sodium and potassium
- Liver enzymes, i.e., AST, ALT, Alkaline Phosphatase, γ-glutamyl transferase, bilirubin
- NT-proBNP

Laboratory data listings will be presented in two ways:

- Abnormal values (presented within the data summaries)
- All laboratory data (presented within the data listings, each category of lab data will be presented in its own listing)

The absolute values of each parameter will be summarised at each visit.

Shift tables will be presented showing changes from baseline to each post baseline visit for laboratory parameters classified as Low Value/ Normal/High Value.

**Lab Parameter**	**Normal Range**	**Lab Parameter**	**Normal Range**
Hemoglobin	above 4.3 mmol/L	Creatinine	below 350 micromol/L
Leucocytes	2 to 40 *10^9/L	eGFR	above 20 ml/min
Thrombocytes	above 80 *10^9/L	Albumin	20-50 g/L
D-dimer	Below 1.0	NTproBNP	below 100 pg/ml
pH	7.1 to 7.6	Bilirubin (Total)	below 30 micromol/L
pCO2 in kPa	Any	ASAT	below 200 U/L
pCO2 in mmHg	Any	ALAT	below 200 U/L
HCO3	Any	AF	below 200 U/L
Na	120 to 165 mmol/L	gGT	below 200 U/L
K	2 to 7 mmol/L	if Female <45 years<br>Beta HCG	if greater or equal to 25 then Pregnant

If lab results are repeated at the same visit, the repeated result will be used in summaries (instead of the original one) provided the sample was taken within the visit window, otherwise the original result will be used. All results will be listed.

Lab results at unscheduled visits will be included in the listings but will not be summarised.

Serum pregnancy data will be listed only.

11.6.3 Vital Signs

Vital Signs will be conducted at Screening, and at Day 1-10 and Day 28 (Follow Up). Weight and Height is collected at Baseline and Day 10.

The following parameters will be included: Mean Arterial Pressure (mmHg), temperature (°C), heart rate (Beats/min), blood pressure, respiratory rate(/min), SpO_2 (%) and FiO_2, Cumulative Fluid Balance (over 24 hours) (Liters), Cumulative Urine Production (over 24 hours) (Liters). Glasgow Coma Scale.

The absolute values of the vital signs descriptive statistics will be summarised at each visit for continuous data as well as change from baseline to post baseline records. Where data is categorical (Coma Scale) a shift table baseline to post baseline visits will be presented. Height, weight and BMI at baseline will be summarised within the demography data. All vital signs data will be listed.

For Mean Arterial Pressure, Heart Rate, Respiratory Rate, SpO_2, Temperature and Cumulative Fluid Balance will also be used to produce a shift table for high, Normal and low from baseline to post baseline values using the following limits

Parameter	Normal Range	Parameter	Normal Range
Mean Arterial Pressure	45 and above	SpO_2	75 and above
Heart Rate	40 to 180	Temperature	33 to 41
Respiratory Rate	8 to 40	Cumulative Fluid Balance	-5 to 5

11.6.4 Electrocardiogram

A 12-lead ECG will be completed at Screening and at Day 1,2,4,7,10.

The values of QTc descriptive Statistics and change from baseline will be summarised at each visit.

Any other clinically significant ECG abnormalities will be captured in the patient's electronic health record.

11.7 Study Drug Exposure and Compliance

A 25ml volume of IMP will be administered over 2-hours as an intravenous infusion. This corresponds to a dose of 200mg imatinib (100mg/h), or 25ml placebo (12.5ml/h). Treatment will be administered twice daily (400 mg total daily imatinib dose) for up to 7 days, or until patient is discharged from critical care, if earlier. The first dose of IMP will occur on Day 1, as soon as possible after randomisation. IMP should be administered 12h (± 2h) apart between 06:00 - 12:00 in the morning and 18:00 - 24:00 in the evening. On Day 1, the first dose of IMP may be administered between 04:00 and 12:00 (08:00 ±4h) and the second dose between 16:00 and 24:00 (20:00 ±4h). Patients commencing treatment after 12:00 on Day 1 should receive only one dose of IMP (between 16:00 and 24:00).

Treatment exposure and compliance (%) will be summarised for the FAS on a daily basis due to the design of the trial.

The average number of IMP not administered per patient will be summarised using descriptive statistics per treatment and overall.

The reasons IMP was not administered will be summarised (AE, Transfer, Death, Other Total) by treatment and overall.

All IMP data will be listed.

11.8 Prior and Concomitant Medication

Prior medications are defined as medication that started and stopped before Day 1. Only medications where the stop date is prior to Day 1 will be considered prior. If the stop date is unknown or incomplete and the medications cannot definitely be considered as stopped prior to Day 1 then the medications will be considered as concomitant medications at randomisation or change in concomitant medication, depending on the start date.

Concomitant medications at randomisation are defined as medications that started before Day 1 and either stopped on Day 1 or continued into the study. Partial start dates where the medication cannot definitely be considered as starting prior to Day 1 will lead to a categorisation of the medications as having started on or after dosing.

Change in concomitant medication is defined as medication that started on or after Day 1. If the medication start or stop dates are partial then the rules for prior and concomitant medication, detailed above will be observed prior to assigning a category.

The number (%) of patients reporting the use of any prior medications and the number (%) of patients taking each drug by ATC classification and PT will be summarised using the FAS. This table will be repeated for concomitant medications at randomisation and for change in concomitant medication.

A Subset of the Prior and Concomitant Medication tables for Drugs with known interactions will be produced.

All Prior or Concomitant medications will be listed.

11.9 Ventilation Parameters

The following Continuous variables will be displayed using descriptive statistics for each visit and change from baseline to post baseline records by treatment and Overall.

Variable	Unit	Variable	Unit
Fraction of Inspired Oxygen	-	I:E Ration Fraction	
Tidal Volume	mL	Mean Airway Pressure	
Positive end-expiratory Pressure	cmH2O	Inspiratory time	
Pressure above PEEP	cmH2O	pH	
Flow	Liters	Arterial Partial Pressure of Oxygen pO2	kPa
Plateau Pressure	cmH2O	Arterial Partial Pressure of Carbon Dioxide pCO2	kPa
Peak Pressure	cmH2O	Arterial Bicarbonate HCO3	mmol/L

Oxygenation Index (using pO2 in mmHg)		Alveolar pO2	
I:E ratio, where I=1	-		

All other continuous variables will be listed only.

Mechanical Ventilation Mode will be displayed using number and % for each visit, treatment and overall.

All tables, figures and listings will be subject to independent quality control and visual review. Unique tables will be independently programmed. Findings will be documented in a quality control template and actions taken will also be documented.

11.10 SOFA Score

Vasopressor use will be summarised by N and %.

Mean Arterial Pressure and the SOFA Calculations for PF ratio, Bilirubin, Creatinine, Platelets, will have descriptive statistics reported at each visit and change from baseline to each post baseline visit by treatment and Overall.

11.11 PICCO

Extravascular lung water, GEDI, CI. SVI, SVV, SVRi, GEF, dPmx, will be reported using descriptive statistic by treatment and overall for each visit, and change from baseline to each post dose baseline.

12 LITERATURE CITATIONS/REFERENCES

Aman J, van Bezu J, Damanafshan A, et al. Effective treatment of edema and endothelial barrier dysfunction with imatinib. Circulation. 2012;126(23):2728-2738.

Craig TR, Duffy MJ, Shyamsundar M, McDowell C, O'Kane CM, Elborn JS, McAuley DF. A randomized clinical trial of hydroxymethylglutaryl- coenzyme a reductase inhibition for acute lung injury (The HARP Study). Am J Respir Crit Care Med. 2011 Mar 1;183(5):620-6.

Kaneko T, Kawamura Y, Maekawa T, et al; PiCCO Pulmonary Edema Study Group. Global end-diastolic volume is an important contributor to increased extravascular lung water in patients with acute lung injury and acute respiratory distress syndrome: a multicenter observational study. J Intensive Care. 2014 Apr 1;2(1):25.

Perkins GD, McAuley DF, Thickett DR, Gao F. The beta-agonist lung injury trial (BALTI): a randomized placebo controlled clinical trial. Am J Respir Crit Care Med. 2006 Feb 1;173(3):281-7.

13 LIST OF TABLES, FIGURES AND LISTINGS

13.1 List of Tables

Demographic Data

Table 14.1.1.1	Patient Disposition	All Randomised Patients
Table 14.1.2.1	Study Termination and Primary Reason for Discontinuation of Treatment	All Randomised Patients
Table 14.1.3.1	Demographics and Baseline Characteristics	Intent to Treat Set
Table 14.1.3.2	Demographics and Baseline Characteristics	Per Protocol Set
Table 14.1.3.3	Demographics Admission Condition	Intent to Treat Set
Table 14.1.3.4	Demographics Admission Condition	Per Protocol Set
Table 14.1.4.1	Medical History	Intent to Treat Set
Table 14.1.4.2	Current Medical Conditions	Intent to Treat Set

Efficacy Data

Table 14.2.1.1	Analysis of Primary Endpoint - Change in Extravascular Lung Water Index (EVLWi)	Intent to Treat Set
Table 14.2.1.2	Analysis of Primary Endpoint - Change in Extravascular Lung Water Index (EVLWi)	PP Set
Table 14.2.1.3	Analysis of Primary Endpoint - Change in Extravascular Lung Water Index (EVLWi) – Without Imputation	Intent to Treat Set
Table 14.2.1.4	Analysis of Primary Endpoint - Change in Extravascular Lung Water Index (EVLWi) Without Imputation	PP Set
Table 14.2.2.1	Change from baseline in Extravascular Lung Water Index Day1-7 (EVLWi)	Intent to Treat Set
Table 14.2.2.2	Change from baseline in Pulmonary vascular permeability index	Intent to Treat Set
Table 14.2.2.3	Change from baseline in Oxygenation index	Intent to Treat Set
Table 14.2.2.4	Change from baseline in PaO2/FiO2 ratio	Intent to Treat Set
Table 14.2.2.5	Change from baseline in Airway driving pressure	Intent to Treat Set
Table 14.2.2.6	Change from baseline in Compliance of the respiratory system	Intent to Treat Set

Table 14.2.2.7	Change from baseline in Mechanical power	Intent to Treat Set
Table 14.2.2.8	Change from baseline in SOFA Score	Intent to Treat Set
Table 14.2.2.9	Change from baseline in ventilation free days	Intent to Treat Set
Table 14.2.2.10	Change from baseline in ventilation free days with survival >=28 days (Death categorised as 0)	Intent to Treat Set
Table 14.2.2.11	Change from baseline in ventilation free days with survival >=28 days (Death categorised as -1)	Intent to Treat Set
Table 14.2.2.12	Change from baseline in Duration of mechanical ventilation (days)	Intent to Treat Set
Table 14.2.2.13	Change from baseline in Length of ICU stay (days)	Intent to Treat Set
Table 14.2.2.14	Change from baseline in Hospital length of stay (days)	Intent to Treat Set
Table 14.2.2.15	Change from baseline in EVLW	Intent to Treat Set
Table 14.2.2.16	Change from baseline in GEDI	Intent to Treat Set
Table 14.2.2.17	Change from baseline in CI	Intent to Treat Set
Table 14.2.2.18	Change from baseline in SVI	Intent to Treat Set
Table 14.2.2.19	Change from baseline in SVV	Intent to Treat Set
Table 14.2.2.20	Change from baseline in SVRi	Intent to Treat Set
Table 14.2.2.21	Change from baseline in GEF	Intent to Treat Set
Table 14.2.2.22	Change from baseline in dPmx	Intent to Treat Set
Table 14.2.3.1	28-day mortality	Intent to Treat Set
Table 14.2.3.2	Blood Cell Count	Intent to Treat Set
Table 14.2.3.3	Kidney function	Intent to Treat Set
Table 14.2.3.4	Liver enzymes	Intent to Treat Set
Table 14.2.3.5	NT-proBNP	Intent to Treat Set

Table 14.2.3.6	Corrected QT Interval for ECG	Intent to Treat Set

Safety Data

Table 14.3.1.1	Treatment-Emergent Adverse Events	Safety Set
Table 14.3.1.2	Treatment-Emergent Adverse Events, by SOC and PT	Safety Set
Table 14.3.1.3	Severe Treatment-Emergent Adverse Events, by SOC and PT	Safety Set
Table 14.3.1.4	Serious Treatment-Emergent Adverse Events, by SOC and PT	Safety Set
Table 14.3.1.5	Drug-Related Treatment-Emergent Adverse Events, by SOC and PT	Safety Set
Table 14.3.1.6	Serious Drug-Related Treatment-Emergent Adverse Events, by SOC and PT	Safety Set
Table 14.3.1.7	Treatment-Emergent Adverse Events Leading to Withdrawal from the Study, by SOC and PT	Safety Set
Table 14.3.2.1	Listing of Deaths	Safety Set
Table 14.3.2.2	Listing of Serious Adverse Events	Safety Set
Table 14.3.2.3	Listing of Withdrawals Due to Adverse Events	Safety Set
Table 14.3.4.1	Listing of Abnormal Laboratory Values	Safety Set
Table 14.3.5.1	Shift Table of Laboratory Parameters	Intent to Treat Set
Table 14.3.5.2	Change from Baseline of Laboratory Parameters	Intent to Treat Set
Table 14.3.6	Vital Signs, Change from Baseline	Safety Set
Table 14.3.6.1	Glasgow Coma Scale	Safety Set
~~Table 14.3.7~~	~~Shift Table of Physical Examination~~	~~Safety Set~~
Table 14.3.8	Study Drug Exposure and Compliance	Safety Set
Table 14.3.9.1	Prior Medications	Intent to Treat Set
Table 14.3.9.2	Concomitant Medications	Intent to Treat Set

Table 14.3.9.3	Changes in Concomitant Medications	Intent to Treat Set

13.2 List of Figures

Figure 1	Primary Endpoint: Boxplot per group per timepoint	Intent to Treat Set
Figure 2	Primary Endpoint Summarizing Individual Trajectories	Intent to Treat Set
Figure 3	Kaplan-Meier Plot of Time to Death	Intent to Treat Set
Figure 4	Individual Patient PK Plots	Intent to Treat Set

13.3 List of Listings

Patient Data Listings

Listing 16.2.1.1	Patient Status
Listing 16.2.1.2	Discontinued Patients
Listing 16.2.1.3	Study Drug Exposure and Compliance
Listing 16.2.1.4	Patient Visit Dates
Listing 16.2.2	Protocol Deviations
Listing 16.2.3	Analysis Datasets
Listing 16.2.4.1	Demographic Data
Listing 16.2.4.2	Hospitalisation
Listing 16.2.4.3	Medical History
Listing 16.2.4.4	Inclusion Measurements
Listing 16.2.6.1	PICCO Data
Listing 16.2.6.2	Ventilation Parameters
Listing 16.2.6.3	PD Parameters (LUS)
Listing 16.2.6.4	SOFA Score
Listing 16.2.7.1	Adverse Event Listing
Listing 16.2.8.1	Laboratory Measurements
Listing 16.2.8.2	Laboratory Measurements: Pregnancy Data
Listing 16.2.9.1	Vital Signs Data
~~Listing 16.2.10.1~~	~~PE~~
Listing 16.2.11.1	ECG
Listing 16.2.12.1	Prior and Concomitant Medication Data
Listing 16.2.13.1	Final Status

Listing 16.2.14.1	Analysis Log output for Table 14.2.1.1
Listing 16.2.14.2	Analysis Log output for Table 14.2.1.2
Listing 16.2.14.3	Analysis Log output for Table 14.2.1.3
Listing 16.2.14.4	Analysis Log output for Table 14.2.1.4
Listing 16.2.14.5	Analysis Log output for Table 14.2.2.1
Listing 16.2.14.6	Analysis Log output for Table 14.2.2.2
Listing 16.2.14.7	Analysis Log output for Table 14.2.2.3
Listing 16.2.14.8	Analysis Log output for Table 14.2.2.4
Listing 16.2.14.9	Analysis Log output for Table 14.2.2.5
Listing 16.2.14.10	Analysis Log output for Table 14.2.2.6
Listing 16.2.14.11	Analysis Log output for Table 14.2.2.7
Listing 16.2.14.12	Analysis Log output for Table 14.2.2.8
Listing 16.2.14.13	Analysis Log output for Table 14.2.2.9
Listing 16.2.14.14	Analysis Log output for Table 14.2.2.10
Listing 16.2.14.15	Analysis Log output for Table 14.2.2.11
Listing 16.2.14.16	Analysis Log output for Table 14.2.2.12
Listing 16.2.14.17	Analysis Log output for Table 14.2.2.13
Listing 16.2.14.18	Analysis Log output for Table 14.2.3.1
Listing 16.2.14.19	Analysis Log output for Table 14.2.3.2
Listing 16.2.14.20	Analysis Log output for Table 14.2.3.3
Listing 16.2.14.21	Analysis Log output for Table 14.2.3.4
Listing 16.2.14.22	Analysis Log output for Table 14.2.3.5
Listing 16.2.14.23	Analysis Log output for Table 14.2.3.6
Listing 16.2.14.24	Analysis Log output for Table 14.4.2.1
Listing 16.2.14.25	Analysis Log output for Table 14.4.2.2

14 SHELLS FOR TABLES, FIGURES AND LISTINGS

The intended layouts for tables, figures and listings are presented. However, it may be appropriate to change the layouts, upon review of the data available, for completeness and clarity.

QCd output will be produced as Rich Text Format (.rtf) files for convenient inclusion in the CSR. The default tables, figures and listings (TFL) layout will be as follows:

**Orientation**	A4 Landscape
**Margins**	Top: 2.54 cm<br>Bottom: 2.54 cm<br>Left: 2.54 cm<br>Right: 2.54 cm
**Font**	Courier New 9pt
**Headers** (Centre)	Sponsor Protocol Number, TFL Number, Title, Analysis Set
**Footers** (Left)	Source Listing, Date/Time TFL Generated, Page Number, i.e. Page x of y

Listing shells are displayed within this document without the comments field but, should there be any comments recorded for the represented data, this field will be added to the listing. In addition, at the time of programming, footnotes will be added to the listing, table or figure as needed. All footnotes will be used for purposes of clarifying the presentation

Should the number of variables within a listing or table be too great to fit on one page without compromising clarity, then the variables will be split across multiple subsequent pages and key identifying variables replicated with these (i.e. subject number, visit etc). The differing pages will be identified using a sequential number which will follow the TFL title, i.e. xxxx - (1), xxxx - (2).

2020-005447-23 ddMMMyyyy HH:MM

Table 14.1.1.1 Patient Disposition (All Randomised Patients)

	Imatinib	Placebo	Total
Screened Patients			xx
Randomised Patients	xx (xx.x%)	xx (xx.x%)	xx (xx.x%)
Safety Set	xx (xx.x%)	xx (xx.x%)	xx (xx.x%)
Intent to Treat Set	xx (xx.x%)	xx (xx.x%)	xx (xx.x%)
PP Set	xx (xx.x%)	xx (xx.x%)	xx (xx.x%)

The denominator for each percentage is the number of randomized patients in the column.

Source: Listing 16.x.x

Path\Filename Page x/y

2020-005447-23 ddMMMyyyy HH:MM

Table 14.1.2.1 Study Termination and Primary Reason for Discontinuation of Treatment (All Randomised Patients)

	Imatinib	Placebo	Total
Randomised	xx	xx	xx
Completed	xx (xx.x%)	xx (xx.x%)	xx (xx.x%)
Early withdrawal	xx (xx.x%)	xx (xx.x%)	xx (xx.x%)
Main reason for Discontinuing Treatment*			
xxxxxxx	xx (xx.x%)	xx (xx.x%)	xx (xx.x%)
xxxxxxx	xx (xx.x%)	xx (xx.x%)	xx (xx.x%)
xxxxxxx	xx (xx.x%)	xx (xx.x%)	xx (xx.x%)
xxxxx	xx (xx.x%)	xx (xx.x%)	xx (xx.x%)
xxxxxx	xx (xx.x%)	xx (xx.x%)	xx (xx.x%)
Etc	xx (xx.x%)	xx (xx.x%)	xx (xx.x%)
Patient status upon study conclusion			
Uninfected	xx (xx.x%)	xx (xx.x%)	xx (xx.x%)
Ambulatory	xx (xx.x%)	xx (xx.x%)	xx (xx.x%)
Hospitalized	xx (xx.x%)	xx (xx.x%)	xx (xx.x%)
Death	xx (xx.x%)	xx (xx.x%)	xx (xx.x%)

The denominator for each percentage is the number of randomized patients in the column.
*The denominator is the number of early withdrawal subjects.

Source: Listing 16.x.x

Path\Filename

Page x/y

Table 14.1.3.1 Demographics and Baseline Characteristics (Intent to Treat Set)

		Imatinib (N=xx)	Placebo (N=xx)	Total (N=xx)
Age (years) at time of Consent	N	xx	xx	xx
	Mean	xx.x	xx.x	xx.x
	SD	xx.xx	xx.xx	xx.xx
	Median	xx.x	xx.x	xx.x
	Minimum	Xx	Xx	Xx
	Maximum	xx	xx	xx
Gender				
	Male	xx (xx.x%)	xx (xx.x%)	xx (xx.x%)
	Female	xx (xx.x%)	xx (xx.x%)	xx (xx.x%)
Height (cm)	N	xx	xx	xx
	Mean	xx.x	xx.x	xx.x
	SD	xx.xx	xx.xx	xx.xx
	Median	xx.x	xx.x	xx.x
	Minimum	Xx	Xx	Xx
	Maximum	xx	xx	xx
Weight (kg)	N	xx	xx	xx
	Mean	xx.x	xx.x	xx.x
	SD	xx.xx	xx.xx	xx.xx
	Median	xx.x	xx.x	xx.x
	Minimum	Xx	Xx	Xx
	Maximum	xx	xx	xx
BMI (kg/m2)	N	xx	xx	xx
	Mean	xx.x	xx.x	xx.x
	SD	xx.xx	xx.xx	xx.xx

	Median	xx.x	xx.x	xx.x
	Minimum	Xx	Xx	Xx
	Maximum	xx	xx	xx
Predicted Weight (kg)	N	xx	xx	xx
	Mean	xx.x	xx.x	xx.x
	SD	xx.xx	xx.xx	xx.xx
	Median	xx.x	xx.x	xx.x
	Minimum	Xx	Xx	Xx
	Maximum	xx	xx	xx
Has the Patient Ever Smoked				
	Yes	xx (xx.x%)	xx (xx.x%)	xx (xx.x%)
	No	xx (xx.x%)	xx (xx.x%)	xx (xx.x%)
Does the Patient Still Smoke				
	Yes	xx (xx.x%)	xx (xx.x%)	xx (xx.x%)
	No	xx (xx.x%)	xx (xx.x%)	xx (xx.x%)
Alcohol Abuse upon Inclusion				
	Yes	xx (xx.x%)	xx (xx.x%)	xx (xx.x%)
	No	xx (xx.x%)	xx (xx.x%)	xx (xx.x%)
Admission Type	Medical	xx (xx.x%)	xx (xx.x%)	xx (xx.x%)
	Planned Surgical	xx (xx.x%)	xx (xx.x%)	xx (xx.x%)
	Emergency Surgical	xx (xx.x%)	xx (xx.x%)	xx (xx.x%)

The denominator for each percentage is the number of non-missing observations within the column.

Source: Listing 16.x.x

Path\Filename

Page x/y

Programming note: This table will be repeated for table 14.1.3.2.

2020-005447-23 ddMMMyyyy

Table 14.1.3.3 Demographics Admission Condition (Intent to Treat Set)

		Imatinib (N=xx)	Placebo (N=xx)	Total (N=xx)
Estimated Number of Days since Onset of Symptoms related to COVID	N	xx	xx	xx
	Mean	xx.x	xx.x	xx.x
	SD	xx.xx	xx.xx	xx.xx
	Median	xx.x	xx.x	xx.x
	Minimum	Xx	Xx	Xx
	Maximum	xx	xx	xx
Days since Positive SARS CoV-2 Test				
	N	xx	xx	xx
	Mean	xx.x	xx.x	xx.x
	SD	xx.xx	xx.xx	xx.xx
	Median	xx.x	xx.x	xx.x
	Minimum	Xx	Xx	Xx
	Maximum	xx	xx	xx
Non-Covid Infection	Yes	xx (xx.x%)	xx (xx.x%)	xx (xx.x%)
	No	xx (xx.x%)	xx (xx.x%)	xx (xx.x%)
Non-Covid Pneumonia	Yes	xx (xx.x%)	xx (xx.x%)	xx (xx.x%)
	No	xx (xx.x%)	xx (xx.x%)	xx (xx.x%)
Pancreatitis	Yes	xx (xx.x%)	xx (xx.x%)	xx (xx.x%)
	No	xx (xx.x%)	xx (xx.x%)	xx (xx.x%)
Shock	Yes	xx (xx.x%)	xx (xx.x%)	xx (xx.x%)
	No	xx (xx.x%)	xx (xx.x%)	xx (xx.x%)

High Risk Trauma	Traumatic Brain Injury	xx (xx.x%)	xx (xx.x%)	xx (xx.x%)
	Smoke Inhalation	xx (xx.x%)	xx (xx.x%)	xx (xx.x%)
	Near Drowning	xx (xx.x%)	xx (xx.x%)	xx (xx.x%)
	Lung Contusion	xx (xx.x%)	xx (xx.x%)	xx (xx.x%)
	Multiple Fractures	xx (xx.x%)	xx (xx.x%)	xx (xx.x%)
	Not Applicable	xx (xx.x%)	xx (xx.x%)	xx (xx.x%)
High Risk Surgery	Orthopedic Spine	xx (xx.x%)	xx (xx.x%)	xx (xx.x%)
	Acute Abdomen	xx (xx.x%)	xx (xx.x%)	xx (xx.x%)
	Cardiac	xx (xx.x%)	xx (xx.x%)	xx (xx.x%)
	Aortic Vascular	xx (xx.x%)	xx (xx.x%)	xx (xx.x%)
	Not Applicable	xx (xx.x%)	xx (xx.x%)	xx (xx.x%)

The denominator for each percentage is the number of non-missing observations within the column.

Source: Listing 16.x.x

Path\Filename

Page x/y

Programming note: This table will be repeated for table 14.1.3.4.

Table 14.1.4.1.1 Medical History (Intent to Treat Set)

		Imatinib (N=xx)	Placebo (N=xx)	Total (N=xx)
Number of Patients with Any medical history		xx (xx.x%)	xx (xx.x%)	xx (xx.x%)
COPD	Yes	xx (xx.x%)	xx (xx.x%)	xx (xx.x%)
	No	xx (xx.x%)	xx (xx.x%)	xx (xx.x%)
	Total	xx (xx.x%)	xx (xx.x%)	xx (xx.x%)
Heart Failure	Yes	xx (xx.x%)	xx (xx.x%)	xx (xx.x%)
	No	xx (xx.x%)	xx (xx.x%)	xx (xx.x%)
	Total	xx (xx.x%)	xx (xx.x%)	xx (xx.x%)
Etc				
Diabetes Mellitus	Yes	xx (xx.x%)	xx (xx.x%)	xx (xx.x%)
	Uncomplicated*	xx (xx.x%)	xx (xx.x%)	xx (xx.x%)
	End-Organ Damage*	xx (xx.x%)	xx (xx.x%)	xx (xx.x%)
	Non/Diet Controlled	xx (xx.x%)	xx (xx.x%)	xx (xx.x%)
	Total	xx (xx.x%)	xx (xx.x%)	xx (xx.x%)
Solid Malignancy	Yes	xx (xx.x%)	xx (xx.x%)	xx (xx.x%)
	No	xx (xx.x%)	xx (xx.x%)	xx (xx.x%)
	Total	xx (xx.x%)	xx (xx.x%)	xx (xx.x%)
	Localized**	xx (xx.x%)	xx (xx.x%)	xx (xx.x%)
	Metastatic**	xx (xx.x%)	xx (xx.x%)	xx (xx.x%)
Liver Disease	Yes	xx (xx.x%)	xx (xx.x%)	xx (xx.x%)

No	xx (xx.x%)	xx (xx.x%)	xx (xx.x%)
Total	xx (xx.x%)	xx (xx.x%)	xx (xx.x%)
Mild***	xx (xx.x%)	xx (xx.x%)	xx (xx.x%)
Moderate/Severe***	xx (xx.x%)	xx (xx.x%)	xx (xx.x%)

The denominator for each percentage is the number of patients within the column.* Denominator is the number of patients with non diet controlled diabetes melitus. **Denominator is the number of patients with Solid Malignancy.***Denominator is the number of patients with liver disease.

Medical history refers to conditions which stopped prior to or at the screening visit.

Source: Listing 16.x.x

Path\Filename Page x/y

This layout also applies to:
Table 14.1.4.2 Current Medical Conditions (Intent to Treat Set) [Programmer's Note: Update footnote to medical conditions definition.]

2020-005447-23 ddMMMyyyy

Table 14.2.1.1 Analysis of Primary Endpoint - Change in Extravascular Lung Water Index (EVLWi) (Intent to Treat Set)

		Imatinib (N=xx)	Placebo (N=xx)	Total (N=xx)	p-value*
Extravascular Lungwater Index at Baseline	N	xx (xx.x%)	xx (xx.x%)	xx (xx.x%)	
	Mean	xx.x	xx.x	xx.x	
	SD	xx.x	xx.x	xx.x	
	Median	xx.x	xx.x	xx.x	
	Minimum	xx.x	xx.x	xx.x	
	Maximum	xx.x	xx.x	xx.x	
Extravascular Lungwater Index at Day 4	N	xx (xx.x%)	xx (xx.x%)	xx (xx.x%)	
	Mean	xx.x	xx.x	xx.x	
	SD	xx.x	xx.x	xx.x	
	Median	xx.x	xx.x	xx.x	
	Minimum	xx.x	xx.x	xx.x	
	Maximum	xx.x	xx.x	xx.x	
Change from Baseline to Day 4	N	xx (xx.x%)	xx (xx.x%)	xx (xx.x%)	x.xxxxxxxxx
	Mean	xx.x	xx.x	xx.x	
	SD	xx.x	xx.x	xx.x	
	Median	xx.x	xx.x	xx.x	
	IQR	xx.x	xx.x	xx.x	
	Minimum	xx.x	xx.x	xx.x	
	Maximum	xx.x	xx.x	xx.x	

The denominator for each percentage is the number of patients within the column.
*T-test in case of normal distribution, Mood test in case of non-normal distribution.
Source: Listing 16.x.x
Path\Filename Page x/y
Programming note: This table will be repeated for Table 14.2.1.2, Table 14.2.1.3, Table 14.2.1.4, Table 14.2.3.6, Table 14.2.1.3 and Table 14.2.1.4, there the footnote should read

2020-005447-23 ddMMMyyyy

Table 14.2.2.1 Change from baseline in Extravascular Lung Water Index Day 1-7 (EVLWi) (Intent to Treat Set)

	Imatinib (N=xx)	Placebo (N=xx)	Difference in LS Means (95%CI)	p-value*

Extravascular Lungwater Index at Baseline	N	xx (xx.x%)	xx (xx.x%)		
	Mean	xx.x	xx.x		
	SD	xx.x	xx.x		
	Median	xx.x	xx.x		
	Minimum	xx.x	xx.x		
	Maximum	xx.x	xx.x		
Extravascular Lungwater Index at Day 7	N	xx (xx.x%)	xx (xx.x%)		
	Mean	xx.x	xx.x		
	SD	xx.x	xx.x		
	Median	xx.x	xx.x		
	Minimum	xx.x	xx.x		
	Maximum	xx.x	xx.x		
Change from Baseline to Day 7	N	xx (xx.x%)	xx (xx.x%)		
	Mean	xx.x	xx.x		
	SD	xx.xx	xx.xx		
	Median	xx.x	xx.x		
	Minimum	xx.x	xx.x		
	Maximum	xx.x	xx.x		
Mixed Model for Repeated Measures	Treatment: LS Mean (95% CI)	x.xx(x.xx-x.xx)	x.xx(x.xx-x.xx)	x.xx(x.xx-x.xx)	x.xxxxxxxxx
	Centre				x.xxxxxxxxx

The denominator for each percentage is the number of patients within the column.
*MMRM model stratified by centre.

Source: Listing 16.x.x
Path\Filename

This layout also applies to:
Table 14.2.2.2 Change from baseline in Pulmonary vascular permeability index (Intent to Treat Set)

Table 14.2.2.3 Change from baseline in Oxygenation index (Intent to Treat Set)

Table 14.2.2.4 Change from baseline in PaO2/FiO2 ratio (Intent to Treat Set)

Table 14.2.2.5 Change from baseline in Airway driving pressure (Intent to Treat Set)

Table 14.2.2.6 Change from baseline in Compliance of the respiratory system (Intent to Treat Set)

Table 14.2.2.7 Change from baseline in Mechanical power (Intent to Treat Set)

Table 14.2.2.8 Change from baseline in SOFA Score (Intent to Treat Set)

Table 14.2.2.9 Change from baseline in ventilation free days (Intent to Treat Set) Footnote for Two sample T Test

Table 14.2.2.10 Change from baseline in ventilation free days with survival >=28 days (Death categorised as 0) (Intent to Treat Set) Footnote for Two sample T Test

Table 14.2.2.11 Change from baseline in ventilation free days with survival >=28 days (Death categorised as -1) (Intent to Treat Set) Footnote for Two sample T Test

Table 14.2.2.12 Change from baseline in Duration of mechanical ventilation (days) (Intent to Treat Set) Footnote for Two sample T Test

Table 14.2.2.13 Change from baseline in Length of ICU stay (days) (Intent to Treat Set) Footnote for Two sample T Test

Table 14.2.2.14 Change from baseline in Hospital length of stay (days) (Intent to Treat Set) Footnote for Two sample T Test

Programming note for these tables: repeat Descriptive Statistics for all timepoint

2020-005447-23 ddMMMyyyy

Table 14.2.2.15 Change from baseline in Extravascular Lung Water (EVLW) (Intent to Treat Set)

		Imatinib (N=xx)	Placebo (N=xx)	Total (N=xx)
Extravascular Lungwater at Baseline	N	xx (xx.x%)	xx (xx.x%)	xx (xx.x%)
	Mean	xx.x	xx.x	xx.x
	SD	xx.x	xx.x	xx.x
	Median	xx.x	xx.x	xx.x
	Minimum	xx.x	xx.x	xx.x
	Maximum	xx.x	xx.x	xx.x
Extravascular Lungwater at Day 7	N	xx (xx.x%)	xx (xx.x%)	xx (xx.x%)
	Mean	xx.x	xx.x	xx.x
	SD	xx.x	xx.x	xx.x
	Median	xx.x	xx.x	xx.x
	Minimum	xx.x	xx.x	xx.x
	Maximum	xx.x	xx.x	xx.x
Change from Baseline to Day 7	N	xx (xx.x%)	xx (xx.x%)	xx (xx.x%)
	Mean	xx.x	xx.x	xx.x
	SD	xx.xx	xx.xx	xx.xx
	Median	xx.x	xx.x	xx.x
	Minimum	xx.x	xx.x	xx.x
	Maximum	xx.x	xx.x	xx.x

The denominator for each percentage is the number of patients within the column.

Source: Listing 16.x.x
Path\Filename

This layout also applies to:

Table 14.2.2.16 Change from baseline in GEDI (Intent to Treat Set)

Table 14.2.2.17 Change from baseline in CI (Intent to Treat Set)

Table 14.2.2.18 Change from baseline in SVI (Intent to Treat Set)

Table 14.2.2.19 Change from baseline in SVV (Intent to Treat Set)

Table 14.2.2.20 Change from baseline in SVRi (Intent to Treat Set)

Table 14.2.2.21 Change from baseline in GEF (Intent to Treat Set)

Table 14.2.2.22 Change from baseline in dPmx (Intent to Treat Set)

2020-005447-23 ddMMMyyyy

Table 14.2.3.1 28-day mortality

		Imatinib (N=xx)	Placebo (N=xx)	Hazard Ratio (95% CI)		(p-value)
Dead	Yes	xx (xx.x%)	xx (xx.x%)			
	No	xx (xx.x%)	xx (xx.x%)			
cox Regression Model				XXXXXX	(xx.x-xx.x)	x.xxxx
Log -Rank Test	Treatment					x.xxxx
	Centre					x.xxxx
	< other Covariates?>					x.xxxx
	etc					

Source: Listing 16.x.x
Path\Filename

Table 14.2.3.2 Blood Cell Count (Intent to Treat Set)

		Imatinib (N=xx)	Placebo (N=xx)	Difference in LS Means (95%CI)	p-value*
Parameter (Unit)					
Baseline	N	xx (xx.x%)	xx (xx.x%)		
	Mean	xx.x	xx.x		
	SD	xx.x	xx.x		
	Median	xx.x	xx.x		
	Minimum	xx.x	xx.x		
	Maximum	xx.x	xx.x		
Day 1	N	xx (xx.x%)	xx (xx.x%)		
	Mean	xx.x	xx.x		
	SD	xx.x	xx.x		
	Median	xx.x	xx.x		
	Minimum	xx.x	xx.x		
	Maximum	xx.x	xx.x		
Change from Baseline to Day 1	N	xx (xx.x%)	xx (xx.x%)		
	Mean	xx.x	xx.x		
	SD	xx.x	xx.x		
	Median	xx.x	xx.x		
	IQR	xx.x	xx.x		
	Minimum	xx.x	xx.x		
	Maximum	xx.x	xx.x		
Repeat for all days					
Mixed Model for Repeated Measures	Treatment: LS Mean (95%CI)	x.xx(x.xx-x.xx)	x.xx(x.xx-x.xx)	x.xx(x.xx-x.xx)	x.xxxxxxxxx
	< other Covariates>				x.xxxxxxxxx
Repeat for each parameter					

The denominator for each percentage is the number of patients within the column.
*T-test in case of normal distribution, Mood test in case of non-normal distribution.

Source: Listing 16.x.x
Path\Filename

Programming note: Parameters are: RBC, WBC, Thrombocytes

This layout also applies to:
Table 14.2.3.3 Kidney function (Intent to Treat Set) (Programming note: parameters are creatinine, eGFR)

Table 14.2.3.4 Liver enzymes (Intent to Treat Set) (Programming note: parameters are ALT, AST, Bilirubin, y-glutamyl transferase, alkaline phosphatase)

Table 14.2.3.5 NT-proBNP (Intent to Treat Set)

2020-005447-23 ddMMMyyyy

Table 14.3.1.1 Treatment-Emergent Adverse Events (Safety Set)

	Imatinib (N=xx)		Placebo (N=xx)		Total (N=xx)	
	E	n (%)	E	n (%)	E	n (%)
Treatment-Emergent Adverse Events	xx	xx (xx.x%)	xx	xx (xx.x%)	xx	xx (xx.x%)
Severe Treatment-Emergent Adverse Events	xx	xx (xx.x%)	xx	xx (xx.x%)	xx	xx (xx.x%)
Serious Treatment-Emergent Adverse Events	xx	xx (xx.x%)	xx	xx (xx.x%)	xx	xx (xx.x%)
Drug-Related Treatment-Emergent Adverse Events	xx	xx (xx.x%)	xx	xx (xx.x%)	xx	xx (xx.x%)
Serious Drug-Related Treatment-Emergent Adverse Events	xx	xx (xx.x%)	xx	xx (xx.x%)	xx	xx (xx.x%)
Treatment-Emergent Adverse Events leading to Withdrawal from study	xx	xx (xx.x%)	xx	xx (xx.x%)	xx	xx (xx.x%)

The table presents number of events (E) and number and percentage of patients (n(%))
The denominator for each percentage is the number of patients within the column

Source: Listing 16.x.x
Path\Filename

Page x/y

2020-005447-23 ddMMMyyyy

Table 14.3.1.2 Treatment-Emergent Adverse Events, by SOC and PT (Safety Set)

	Imatinib (N=xx)		Placebo (N=xx)		Total (N=xx)	
	E	n (%)	E	n (%)	E	n (%)
Any Treatment-Emergent Adverse Event	xx	xx (xx.x%)	xx	xx (xx.x%)	xx	xx (xx.x%)
SOC	xx	xx (xx.x%)	xx	xx (xx.x%)	xx	xx (xx.x%)
PT	xx	xx (xx.x%)	xx	xx (xx.x%)	xx	xx (xx.x%)
PT	xx	xx (xx.x%)	xx	xx (xx.x%)	xx	xx (xx.x%)
SOC Etc	xx	xx (xx.x%)	xx	xx (xx.x%)	xx	xx (xx.x%)
Etc						

The table presents number of events (E) and number and percentage of patients (n(%)).
The denominator for each percentage is the number of patients within the column.

Source: Listing 16.2.7
Path\Filename

Page x/y

This layout also applies to:
Table 14.3.1.3 Severe Treatment-Emergent Adverse Events, by SOC and PT (Safety Set)
Table 14.3.1.4 Serious Treatment-Emergent Adverse Events, by SOC and PT (Safety Set)
Table 14.3.1.5 Drug-Related Treatment-Emergent Adverse Events, by SOC and PT (Safety Set)
Table 14.3.1.6 Serious Drug-Related Treatment-Emergent Adverse Events, by SOC and PT (Safety Set)
Table 14.3.1.7 Treatment-Emergent Adverse Events Leading to Withdrawal from the Study, by SOC and PT (Safety Set)

2020-005447-23 ddMMMyyyy

Table 14.3.2.1 Listing of Deaths (Safety Set)

Treatment	Patient number	Date of death	Cause of death
xxxxxx	xxx-xxxx	ddMMMyyyy	xxxxxxxxxxxxxxxxxxx
xxxxxx	xxx-xxxx	ddMMMyyyy	xxxxxxxxxxxxxxxxxxx
Etc			

Path\Filename

Page x/y

2020-005447-23 ddMMMyyyy

Table 14.3.2.2 Listing of Serious Adverse Events (Safety Set)

Treatment /<br>Patient number	Adverse Event<br>PT<br>SOC	Date of onset (stop date ongoing)/<br>Study Day	Duration of event (days)<br>AE Abated after IMP dose Changed/Stopped	Nature of AE<br>Nature of SAE	Type of AE<br>Expected AE	Outcome	Severity	Relationship to<br>study drug /Action<br>Taken with Study<br>Drug	If Action is not equal to None; then Event reoccurred After IMP Restarted?	SAE?<br>If yes<br>Seriousness Criteria
xxxxx	xxxxxxxxxx<br>xxxxxxxx<br>xxxx	ddMMMyyyy<br>(ddMMMyyyy)<br>xx<br>Etc	Xx<br>No/Yes/Not Applicable	xxxxxxxxxxx	Gastrointestinal<br>xxxxx<br>Other <Specify><br>etc<br>Yes/No	Resolved / Resolved without sequelae / Etc	Mild /Moderate<br>/Severe<br>/Life-threatening<br>/Death	None/ Temporary discontinuation/ Etc	Yes/No. Not<br>Applicable	Yes/No<br>Death<br>Life-threatening. ..
Etc										

Path\Filename

Page x/y

This layout also applies to:
Table 14.3.2.3 Listing of Withdrawals Due to Adverse Events (Safety Set)

2020-005447-23 ddMMMyyyy

Table 14.3.4.1 Listing of Abnormal Laboratory Values (Safety Set)

Treatment	Patient number	Visit	Laboratory Parameter	Value
xxxxxx	xxx-xxxx	Screening	xxxxxxxxxxxxxxxxxxx	xx.xx
			xxxxxxxxxxxxxxxxxxx	xx.xx
			Etc	
		Day 1		
	Etc			
Etc				

Abnormal is defined as above or below the normal range.
Path\Filename

Page x/y

2020-005447-23 ddMMMyyyy

Table 14.3.5.1 Shift Table of Laboratory Parameters
(Intent to Treat Set)

		Imatinib (N=xxx)	Placebo (N=xxx)
parameter			
Screening	N	xx	xx
	Below	xx (xx.x%)	xx (xx.x%)
	Within	xx (xx.x%)	xx (xx.x%)
	Above	xx (xx.x%)	xx (xx.x%)
Timepoint	N	xx	xx
	Below	xx (xx.x%)	xx (xx.x%)
	Within	xx (xx.x%)	xx (xx.x%)
	Above	xx (xx.x%)	xx (xx.x%)
Change from Screening to Timepoint			
	Below -> Below	xx (xx.x%)	xx (xx.x%)
	Below ->Normal	xx (xx.x%)	xx (xx.x%)
	Below ->Above	xx (xx.x%)	xx (xx.x%)
	Within ->Below	xx (xx.x%)	xx (xx.x%)
	Within ->Within	xx (xx.x%)	xx (xx.x%)
	Within ->Above	xx (xx.x%)	xx (xx.x%)
	Above ->Below	xx (xx.x%)	xx (xx.x%)

Above ->Within	xx (xx.x%)	xx (xx.x%)
Above ->Above	xx (xx.x%)	xx (xx.x%)

Source: Listing 16.x.x
Path\Filename

Page x/y

Please repeat for all catagorical variables, For the shift part of the table please remove lines where the total is 0.

2020-005447-23 ddMMMyyyy

Table 14.3.5.2 Change from Baseline of Laboratory Parameters (Intent to Treat Set)

	Imatinib (N=xx)	Placebo (N=xx)	Total (N=xx)

Haematocrit				
Baseline	N	xx	xx	xx
	Mean	xx.xx	xx.xx	xx.xx
	SD	xx.xx	xx.xx	xx.xx
	Median	xx.xx	xx.xx	xx.xx
	Minimum	xx.x	xx.x	xx.x
	Maximum	xx.x	xx.x	xx.x
Day 1	N	xx	Xx	Xx
	Mean	xx.xx	xx.xx	xx.xx
	Etc			
Change from baseline to Day 1	N	xx	Xx	Xx
	Mean	xx.xx	xx.xx	xx.xx
	Etc			
Etc				
Etc				

Source: Listing 16.x.x.x
Path\Filename

Page x/y

Programming note: start each parameter on a new page

2020-005447-23 ddMMMyyyy

Table 14.3.6 Vital Signs, Change from Baseline (Safety Set)

		Imatinib (N=xx)	Placebo (N=xx)	Total (N=xx)
Temperature (C)				
Baseline	N	xx	xx	xx
	Mean	xx.xx	xx.xx	xx.xx
	SD	xx.xx	xx.xx	xx.xx

	Median	xx.xx	xx.xx	xx.xx
	Minimum	xx.x	xx.x	xx.x
	Maximum	xx.x	xx.x	xx.x
Day 1	N	xx	xx	xx
	Mean	xx.xx	xx.xx	xx.xx
	SD	xx.xx	xx.xx	xx.xx
	Etc.			
Change from baseline	N	xx	xx	xx
Etc	Mean	xx.xx	xx.xx	xx.xx
	SD	xx.xx	xx.xx	xx.xx
	Etc.			

Source: Listing 16.x.x
Path\Filename

Programmer's Note: list of variables consists of: temperature, pulse rate, blood pressure, respiratory rate, SpO2 and FiO2.

2020-005447-23 ddMMMyyyy

Table 14.3.6.1 Glasgow Coma Scale (Safety Set)

		Imatinib (N=xx)	Placebo (N=xx)	Total (N=xxx)
Screening	N	xx	xx	xx
	15	xx (xx.x%)	xx (xx.x%)	xx (xx.x%)
	13-14	xx (xx.x%)	xx (xx.x%)	xx (xx.x%)
	10-12	xx (xx.x%)	xx (xx.x%)	xx (xx.x%)
	6-9	xx (xx.x%)	xx (xx.x%)	xx (xx.x%)
	<6	xx (xx.x%)	xx (xx.x%)	xx (xx.x%)
Timepoint	N	xx	xx	xx
	15	xx (xx.x%)	xx (xx.x%)	xx (xx.x%)
	13-14	xx (xx.x%)	xx (xx.x%)	xx (xx.x%)
	10-12	xx (xx.x%)	xx (xx.x%)	xx (xx.x%)
	6-9	xx (xx.x%)	xx (xx.x%)	xx (xx.x%)
	<6	xx (xx.x%)	xx (xx.x%)	xx (xx.x%)
Change from Screening to Timepoint				
	15 ->15	xx (xx.x%)	xx (xx.x%)	xx (xx.x%)
	15 ->13-14	xx (xx.x%)	xx (xx.x%)	xx (xx.x%)
	15 ->10-12	xx (xx.x%)	xx (xx.x%)	xx (xx.x%)
	15 ->6-9	xx (xx.x%)	xx (xx.x%)	xx (xx.x%)
	15 -> <6	xx (xx.x%)	xx (xx.x%)	xx (xx.x%)
	ETC	xx (xx.x%)	xx (xx.x%)	xx (xx.x%)

Source: Listing 16.x.x
Path\Filename

Page x/y

2020-005447-23 ddMMMyyyy

Table 14.3.8 Study Drug Exposure and Compliance (Safety Set)

		Imatinib (N=xx)	Placebo (N=xx)	Total (N=xx)
<Day>				
All Administered as protocol	Yes	xx (xx.x%)	xx (xx.x%)	xx (xx.x%)
	No	xx (xx.x%)	xx (xx.x%)	xx (xx.x%)
Not Administered*		xx (xx.x%)	xx (xx.x%)	xx (xx.x%)
Partially Administered		xx (xx.x%)	xx (xx.x%)	xx (xx.x%)
Fully Administered, However Interrupted		xx (xx.x%)	xx (xx.x%)	xx (xx.x%)
	Etc.			
Partially Administered**	Death	xx (xx.x%)	xx (xx.x%)	xx (xx.x%)
	Hospital Discharge	xx (xx.x%)	xx (xx.x%)	xx (xx.x%)
	Discharged to Ward	xx (xx.x%)	xx (xx.x%)	xx (xx.x%)
	Other	xx (xx.x%)	xx (xx.x%)	xx (xx.x%)
Total Daily Volume of IMP (ml)	N	xx (xx.x%)	xx (xx.x%)	xx (xx.x%)
	mean	xx.xx	xx.xx	xx.xx
	SD	xx.xxx	xx.xxx	xx.xxx
	Median	xx.xx	xx.xx	xx.xx
	Minimum	xx.x	xx.x	xx.x
	Maximum	xx.x	xx.x	xx.x

*Denominator for each percentage is the number not administered** Denominator for each Percentage is the number of Partially Administered.

Source: Listing 16.x.x
Path\Filename

Page x/y

2020-005447-23 ddMMMyyyy

Table 14.3.9.1 Prior Medications (Intent to Treat Set)

	Imatinib	Placebo	Total

	(N=xx)	(N=xx)	(N=xx)
Any prior medication1	xx (xx.x%)	xx (xx.x%)	xx (xx.x%)
X, XXXXXXXXXXXXXX	xx (xx.x%)	xx (xx.x%)	xx (xx.x%)
XON, XXXXXXXXXXXXX	xx (xx.x%)	xx (xx.x%)	xx (xx.x%)
XONXX, XXXXXXXXXXX	xx (xx.x%)	xx (xx.x%)	xx (xx.x%)
XXXXXXXXXXXXX	xx (xx.x%)	xx (xx.x%)	xx (xx.x%)
Etc	xx (xx.x%)	xx (xx.x%)	xx (xx.x%)
XONXX, XXXXXXXXXXX	xx (xx.x%)	xx (xx.x%)	xx (xx.x%)
XXXXXXXXXXXX	xx (xx.x%)	xx (xx.x%)	xx (xx.x%)
Etc	xx (xx.x%)	xx (xx.x%)	xx (xx.x%)
XON, XXXXXXXXXXXXX	xx (xx.x%)	xx (xx.x%)	xx (xx.x%)
XONXX, XXXXXXXXXXX	xx (xx.x%)	xx (xx.x%)	xx (xx.x%)
XXXXXXXXXXXX	xx (xx.x%)	xx (xx.x%)	xx (xx.x%)
Etc	xx (xx.x%)	xx (xx.x%)	xx (xx.x%)
Etc			
Etc			

[1] Medication that stopped prior to date of first dose.
WHO-DDE version <XX.X>
The denominator for each percentage is the number of patients in the Intent to Treat set within the column.
Source: Listing 16.x.x.x
Path\Filename

Page x/y

This layout also applies to:
Table 14.3.9.2 Concomitant Medications (Intent to Treat Set)

2020-005447-23

ddMMMyyyy

Table 14.3.9.3 Change in Concomitant Medications (Intent to Treat Set)

	Imatinib (N=xx)	Placebo (N=xx)	Total (N=xx)
Any concomitant medication	xx (xx.x%)	xx (xx.x%)	xx (xx.x%)
X, XXXXXXXXXXXXXX	xx (xx.x%)	xx (xx.x%)	xx (xx.x%)
XON, XXXXXXXXXXXXX	xx (xx.x%)	xx (xx.x%)	xx (xx.x%)
XONXX, XXXXXXXXXXX	xx (xx.x%)	xx (xx.x%)	xx (xx.x%)
XXXXXXXXXXXXX	xx (xx.x%)	xx (xx.x%)	xx (xx.x%)
Etc	xx (xx.x%)	xx (xx.x%)	xx (xx.x%)
XONXX, XXXXXXXXXXX	xx (xx.x%)	xx (xx.x%)	xx (xx.x%)
XXXXXXXXXXXX	xx (xx.x%)	xx (xx.x%)	xx (xx.x%)
Etc	xx (xx.x%)	xx (xx.x%)	xx (xx.x%)

X0N, xxxxxxxxxxxxx	xx (xx.x%)	xx (xx.x%)	xx (xx.x%)
X0NXX, xxxxxxxxxxx	xx (xx.x%)	xx (xx.x%)	xx (xx.x%)
xxxxxxxxxxxx	xx (xx.x%)	xx (xx.x%)	xx (xx.x%)
Etc	xx (xx.x%)	xx (xx.x%)	xx (xx.x%)
Etc			
Etc			

WHO-DDE version <XX.X>
The denominator for each percentage is the number of patients in the Intent to Treat set within the column.

Source: Listing 16.x.x.x
Path\Filename

Page x/y

2020-005447-23 ddMMMyyyy

Listing 16.2.1.1 Patient Status

Treatment	Centre/ Patient number	Date of informed Consent	Visit	Status at 08:00 am	Days since ICU administration	Intubated?	Days since intubation
xxxxxx	xxx-xxxx	ddMMMyyyy	ddMMMyyyy	xxxxxxx	xxx	Yes/No	xxx
	xxx-xxxx		ddMMMyyyy	xxxxxxx	xx	Yes/No	xxx
	xxx-xxxx		ddMMMyyyy	xxxxxxx	xxx	Yes/No	xxx
	xxx-xxxx		ddMMMyyyy	xxxxxxx	xx	Yes/No	xxx
Etc							

Path\Filename

2020-005447-23 ddMMMyyyy

Listing 16.2.1.2 Discontinued Patients

Treatment	Centre/ Patient number	First dose date	Last dose date	Date of withdrawal	Main reason for withdrawal
xxxxxx	xxx-xxxx	ddMMMyyyy	ddMMMyyyy	ddMMMyyyy	xxxxxxxxxxxxxxxxxxxxxxxxxxx
	xxx-xxxx	ddMMMyyyy	ddMMMyyyy	ddMMMyyyy	xxxxxxxxxxxxxxxxxxxxxxxxxxx
	xxx-xxxx	ddMMMyyyy	ddMMMyyyy	ddMMMyyyy	xxxxxxxxxxxxxxxxxxxxxxxxxxx
	xxx-xxxx	ddMMMyyyy	ddMMMyyyy	ddMMMyyyy	xxxxxxxxxxxxxxxxxxxxxxxxxxx
Etc					

Path\Filename

2020-005447-23 ddMMMyyyy

Listing 16.2.1.3 Study Drug Exposure and Compliance

Treatment	Centre/ Patient number	Visit	Date	IMP administered?	All IMP doses administered according to	Time of Dose 1	If partially administered then add	Time of Dose 2	If partially administered then add

			protocol? (if no, specify)		volume of IMP administered		volume of IMP administered
xxxxxx	xxx-xxxx	Yes/No	Yes/No	xx:xx	xxxx	xx:xx	xxxx
	xxx-xxxx	Yes/No	Yes/No	xx:xx	xxxx	xx:xx	xxxx
	xxx-xxxx	Yes/No	Yes/No	xx:xx	xxxx	xx:xx	xxxx
Etc							

Path\Filename Page x/y

Make the columns Treatment / Centre/Patient Number / Visit / Date / IMP Administered? / All IMP doses administered according to protocol? (if no specify) / Time of Dose 1 / If partially administered then add volume of IMP administered/ Time of Dose 2 / If partially administered then add volume of IMP administered edited

There are two of those partial volumes, one for each time. Wording might need compressing a bit!

2020-005447-23 ddMMMyyyy

Listing 16.2.1.4 Patient Visit Dates

Treatment	Centre/ Patient number	Visit	Date
xxxxxx	xxx-xxxx	xx	ddMMMyyyy
		xx	ddMMMyyyy
		xx*	ddMMMyyyy
		xx	ddMMMyyyy
Etc			

*Outside the visit window

Path\Filename

Page x/y

2020-005447-23 ddMMMyyyy

Listing 16.2.2 Protocol Deviations

Treatment	Centre/ Patient number	Major deviation	Protocol deviation

xxxxxx	xxx-xxxx	Yes/No	xxxxxxxxxxxxxxxxxxxxxxxxxxxxxxxxxxxxxxxxxxxxxxxxxxxxxxxxxxxxxxxxxxxxxxxxxxxxxxxx
	xxx-xxxx	Yes/No	xxxxxxxxxxxxxxxxxxxxxxxxxxxxxxxxxxxxxxxxxxxxxxxxxxxxxxxxxxxxxxxxxxxxxxxxxxxxxxxx
	xxx-xxxx	Yes/No	xxxxxxxxxxxxxxxxxxxxxxxxxxxxxxxxxxxxxxxxxxxxxxxxxxxxxxxxxxxxxxxxxxxxxxxxxxxxxxxx
	xxx-xxxx	Yes/No	xxxxxxxxxxxxxxxxxxxxxxxxxxxxxxxxxxxxxxxxxxxxxxxxxxxxxxxxxxxxxxxxxxxxxxxxxxxxxxxx

Etc

Path\Filename

Page x/y

2020-005447-23 ddMMMyyyy

Listing 16.2.3 Analysis Datasets

Treatment	Centre/ Patient number	Intent to Treat Set	Per Protocol Set	Safety Set	PK Set	PD Set
xxxxxx	xxx-xxxx	Yes/No	Yes/No	Yes/No	Yes/No	Yes/No
	xxx-xxxx	Yes/No	Yes/No	Yes/No	Yes/No	Yes/No
	xxx-xxxx	Yes/No	Yes/No	Yes/No	Yes/No	Yes/No
	xxx-xxxx	Yes/No	Yes/No	Yes/No	Yes/No	Yes/No
Etc						

Path\Filename

Page x/y

2020-005447-23 ddMMMyyyy

Listing 16.2.4.1 Demographic Data

Treatment	Centre/ Patient number	Date of screening	Age (years)	Gender	Race (If other, please specify)	Weight (kg)	Height (cm)	BMI (kg/m2)	Has the patient ever smoked?	If Yes, number of pack/year /Still smoke	Alcohol abuse upon inclusion
xxxxxx	xxx-xxxx	ddMMMyyyy	xx	xxxx	xxxx	xx.x	xxx.x	xx.x	Yes/No	XXX/ Yes/Ex-Smoker	Yes/No/Unknown
	xxx-xxxx	ddMMMyyyy	xx	xxxx	xxxx	xx.x	xxx.x	xx.x	Yes/No	XXX/ Yes/Ex-Smoker	Yes/No/Unknown
	xxx-xxxx	ddMMMyyyy	xx	xxxx	xxxx	xx.x	xxx.x	xx.x	Yes/No	XXX/ Yes/Ex-Smoker	Yes/No/Unknown
	xxx-xxxx	ddMMMyyyy	xx	xxxx	xxxx	xx.x	xxx.x	xx.x	Yes/No	XXX/ Yes/Ex-Smoker	Yes/No/Unknown
	xxx-xxxx	ddMMMyyyy	xx	xxxx	xxxx	xx.x	xxx.x	xx.x	Yes/No	XXX/ Yes/Ex-Smoker	Yes/No/Unknown
									Yes/No	XXX/ Yes/Ex-Smoker	Yes/No/Unknown
Etc											

2020-005447-23 ddMMMyyyy

Listing 16.2.4.2 Hospitalisation

Treatment	Centre/ Patient number	Date of Hospitalisation	Admission Type	Estimated Number of Days Since Onset of Symptoms	Days Since Positive SARS COV-2 test	Number of ventilator-free days and alive at Day 28	Duration of mechanical ventilation (days) between Day 1 and 28	Length of ICU stay (days) between Day 1 and 28	Hospital length of stay (days) between Day 1 and 28	28-day mortality
xxxxxx	xxx-xxxx	ddMMMyyyy	Medical<br>Planned surgical<br>Emergency Surgical	xxxx	xxxx					
	xxx-xxxx	ddMMMyyyy	xx	xxxx	xxxx					
Etc										

Path\Filename Page x/y

2020-005447-23 ddMMMyyyy

Listing 16.2.4.3 Medical History

Treatment	Centre/ Patient number	Condition	Response
xxxxxx	xxx-xxxx	xxxxxxxxxxxxxx	Yes/No/ Mild/ Moderate/ Severe/ Uncomplicated End-Organ damage/ Localized/Metastatic
		xxxxxxxxxxxxxx	Yes/No/ Mild/ Moderate/ Severe/ Uncomplicated End-Organ damage/ Localized/Metastatic
Etc			

Path\Filename

2020-005447-23 ddMMMyyyy

Listing 16.2.4.4 Inclusion Measurements

Treatment	Centre/ Patient number	Date of Eligibility	Parameter (Unit)	Result
xxxxxx	xxx-xxxx	ddMMMyyyy	FiO2	xxx.x
			PaO2 (kPa)	
			PaO2 (mmHg)	
			PF Ratio	
		ddMMMyyyy	xxxxxxxxxxxxxx	xxx.x
Etc				

Path\Filename

Page x/y

2020-005447-23 ddMMMyyyy

Listing 16.2.6.1 PICCO Data

Treatment	Centre/ Patient number	Visit	Date/Time	Parameter Name	Parameter	Result	Unit

xxxxxx	xxx-xxxx	Baseline	ddMMMYYYY/hh:mm	Extravascular Lungwater	EVLW	xxxx	xxxx
	xxx-xxxx	Day 1	ddMMMYYYY/hh:mm	etc	EVLWi	xxxx	xxxx
	xxx-xxxx	etc	ddMMMYYYY/hh:mm		PVPI	xxxx	xxxx
					GEDI		
					CI		
					dpmx		
CI							
Etc							

Data recorded daily at 6 am +/- 2 hours, * out of window

Path\Filename Page x/y

2020-005447-23 ddMMMyyyy

Listing 16.2.6.2 Ventilation Parameters Data

Treatment	Centre/ Patient number	Visit	Date/Time	Mechanical Ventilation Mode	Parameter	Result	Unit
xxxxxx	xxx-xxxx	xxxxx	ddMMMYYYY/hh:mm	xxxxxxx	xx	xxxx	
				<other: Specify>			
	xxx-xxxx	xxxxx	ddMMMYYYY/hh:mm	etc		xxxx	
	xxx-xxxx	etc	ddMMMYYYY/hh:mm			xxxx	
CI							
Etc							

All Values recorded at moment of lowers PF ratio on specific Days.

Path\Filename Page x/y

2020-005447-23 ddMMMyyyy

Listing 16.2.6.3 PD Parameters (LUS)

Treatment	Centre/ Patient number	Visit	Date/Time	Lung Ultrasound category	Was the test performed?	Zone	Specify

					If Yes, Prone position?		
xxxxxx	xxx-xxxx	Baseline	ddMMMYYYY/hh:mm	Right/Left/ substudy	Yes/No<br>Yes/No	R1 – A/B1/B2/C/Unable to score/Not performed<br>R2 – A/B1/B2/C/Unable to score/Not performed<br>etc	No lung sliding/Subpleural consolidation/Pleural effusion/etc
		Day 1		Right/Left/ substudy	Yes/No<br>Yes/No		
		Etc					
Etc							

Path\Filename

Page x/y

2020-005447-23 ddMMMyyyy

Listing 16.2.6.4 SOFA Score

Treatment	Centre/ Patient number	Visit	Date/Time	Mechanical Ventilation	Mean Arterial Pressure(mmHg) or Vasopressor (microg/kg/min)	SOFA Score

xxxxxx	xxx-xxxx	xxxxxx	ddMMMYYYY/hh:mm	Yes/no	xxxxxx	R1 – A/B1/B2/C/Unable to score/Not performed<br>R2 – A/B1/B2/C/Unable to score/Not performed<br>etc
		xxxxx<br>Etc				
Etc						

Path\Filename

Page x/y

2020-005447-23 ddMMMyyyy

Listing 16.2.7 Adverse Event Listing

Treatment /<br>Patient number	Adverse Event<br>PT<br>SOC	Date of onset (stop date ongoing)/<br>Study Day	Duration of event (days) AE Abated after IMP dose Changed/Stopped	Nature of AE<br>Nature of SAE	Type of AE<br>Expected AE	Outcome	Severity	Relationship to<br>study drug /Action<br>Taken with Study<br>Drug	If Action is not equal to None; then Event reoccurred After IMP Restarted?	SAE?<br>If yes<br>Seriousness Criteria
xxxxx	xxxxxxxxxx<br>xxxxxxxx	ddMMMyyyy<br>(ddMMMyyyy)<br>xx	Xx<br>No/Yes/Not<br>Applicable	xxxxxxxxxxx	Gastrointestinal<br>xxxxx<br>Other <Specify>	Resolved/ Resolved without sequelae/ Etc	Mild /Moderate<br>/Severe	None/ Temporary discontinuation/ Etc	Yes/No. Not<br>Applicable	Yes/No<br>Death<br>Life-threatening...

	xxxx				etc<br>Yes/No		/Life-threatening<br>/Death			
	xxxxxxxxxx<br>xxxxxxxx<br>xxxx	ddMMMyyyy<br>(ddMMMyyyy)<br>xx	Xx<br>No/Yes/Not<br>Applicable	xxxxxxxxxxx		Resolved/ Resolved without sequelae/ Etc	Mild<br>/Moderate<br>/Severe<br>/Life-threatening<br>/Death	None/ Temporary discontinuation/ Etc	Yes/No. Not<br>Applicable	Yes/No<br>Death<br>Life-threatening...
Etc										

2020-005447-23 ddMMMyyyy

Listing 16.2.8.1 Laboratory Measurements

Parameter	Treatment	Centre/ Patient number	Visit	Was sample taken?	Result	Unit	Outcome
Haemoglobin	xxxxxxx	xxx-xxxx	Screening	Yes/ No (reason)	xxx.xx	xxxx	Normal/ Abnormal
			Baseline	Yes/ No (reason)	xxx.xx	xxxx	Normal/ Abnormal
			Day 1	Yes/ No (reason)	xxx.xx	xxxx	Normal/ Abnormal
			Etc		xxx.xx	xxxx	Normal/ Abnormal
		xxx-xxxx	Screening		xxx.xx	xxxx	Normal/ Abnormal
			Etc				
		Etc					
	Etc						
Etc							

Path\Filename

Page x/y

2020-005447-23 ddMMMyyyy

Listing 16.2.8.2 Laboratory Measurements Pregnancy Data

Parameter	Treatment	Centre/ Patient number	Visit	Was sample taken?	Retest	Beta HCG (mIU/ml)	Result
Haemoglobin	xxxxxxx	xxx-xxxx	Baseline	Yes/ No (reason)	yes/no	xxx.xx	xxxx

Etc

Etc

Etc

Path\Filename

Page x/y

2020-005447-23 ddMMMyyyy

Listing 16.2.9.1 Vital Signs Data

Treatment	Centre/ Patient number	Visit	Date of visit	Mean arterial pressure (mmHg)	Body temperature (˚C)	Glasgow Coma Scale	Cumulative fluid balance (liters)	Cumulative urine production (liters)	Heart rate (beats/min)	Respiratory rate (/min)	SpO2 (%)
xxxxxx	xxx-xxxx	xx	ddMMMyyyy	xx.x	xx.x	xx.x	xxx	xxx	xxx	xx.x	xx.x
		xx	ddMMMyyyy	xx.x	xx.x	xx.x	xxx	xxx	xxx	xx.x	xx.x
		xx*	ddMMMyyyy	xx.x	xx.x	xx.x	xxx	xxx	xxx	xx.x	xx.x

xx	ddMMMyyyy	xx.x	xx.x	xx.x	xxx	xxx	xxx	xx.x	xx.x

Etc

*Outside the visit window
Data collected 8 am (+/-1hour)
Path\Filename
Page x/y

2020-005447-23 ddMMMyyyy

Listing 16.2.11.1 12-Lead Electrocardiogram

Treatment	Centre/ Patient number	Visit	Date of visit	Corrected QT Interval (msec)	Outcome
xxxxxx	xxx-xxxx	Baseline	ddMMMyyyy	xx	Normal/ Abnormal
		Day 1	ddMMMyyyy	xx	Normal/ Abnormal
		Etc*			
	Etc				
Etc					

*Outside the visit window

Path\Filename

Page x/y

2020-005447-23 ddMMMyyyy

Listing 16.2.13.1 Final Status

Treatment	Centre/ Patient number	First dose date	Last dose date	Completed treatment (If No, specify and	Patient status upon study conclusion	Intubated?	Days alive from inclusion	Days since hospital admission	Days since ICU admission	Hospital discharge date

				add discontinuation date)			until Day 28			
xxxxxx	xxx-xxxx	ddMMMyyyy	ddMMMyyyy	Yes/No	Uninfected/Ambulatory/hospitalized/death	xx	xx	xx	xx	ddMMMyyyy
	xxx-xxxx	ddMMMyyyy	ddMMMyyyy	Yes/No	Uninfected/Ambulatory/hospitalized/death	xx	xx	xx	xx	ddMMMyyyy
	xxx-xxxx	ddMMMyyyy	ddMMMyyyy	Yes/No	Uninfected/Ambulatory/hospitalized/death	xx	xx	xx	xx	ddMMMyyyy
	xxx-xxxx	ddMMMyyyy	ddMMMyyyy	Yes/No	Uninfected/Ambulatory/hospitalized/death	xx	xx	xx	xx	ddMMMyyyy
Etc										

Path\Filename

[programming note] If Hospital Discharge date is after Day 28 visit, present as "after end of study"?

2020-005447-23 ddMMMyyyy

Listing 16.4.12.1 Prior Medications

Treatment	Centre/ Patient number	Therapy ATC Code PT	Indication	Dose	Unit	Start date (Stop date / Ongoing)	Medication with known strong interactions? (if other, please specify)	Antibiotics received?(if other, please specify)	Potentially relevant?
xxxxxx	xxx-xxxx	xxxxxxxxxxxxxxx xxxxxxxxxxxxx xxxxxxxxxxx	xxxxxxx	xx.x	xxx	ddMMMyyyy (ddMMMyyyy)	Yes (xxx) / No	Yes (xxx) / No	Yes (xxx) / No
		xxxxxxxxxxxxxxx xxxxxxxxxxxxx xxxxxxxxxxx	xxxxxxx	xx.x	xxx	ddMMMyyyy (ddMMMyyyy)	Yes (xxx) / No	Yes (xxx) / No	Yes (xxx) / No
		xxxxxxxxxxxxxxx xxxxxxxxxxxxx xxxxxxxxxxx	xxxxxxx	xx.x	xxx	ddMMMyyyy (ddMMMyyyy)	Yes (xxx) / No	Yes (xxx) / No	Yes (xxx) / No
Etc									

Path\Filename

Page x/y

This layout also applies to:

Listing 16.4.12.2 Concomitant Medications (Programming note: can have stop date as ‘ongoing’)

Listing 16.4.12.3 Changes to Medications

15 APPENDICES

15.1 STUDY FLOWCHART AND/OR SCHEDULE

Study Period	Screening	Baseline	Treatment							Follow up	
**Study Day (± Window)**	**0-1[a]**	**1**	**2**	**3**	**4**	**5**	**6**	**7**	**10**	**11-27[b]**	**28[c] (±3)**
ELIGIBILITY											
(Deferred) Informed consent	X										
Demographics	X										
Relevant medical history[d]	X										
SARS-CoV-2 diagnostic test review[e]	X										
Assess ARDS diagnosis and severity[f]	X										
Inclusion and exclusion criteria	X										
STUDY INTERVENTION											
Randomisation		X[g]									
IMP administration		X[g]	X	X	X	X	X	X			
Treatment with SoC	X	X	X	X	X	X	X	X	X	X	X
STUDY PROCEDURES											
12-lead Electrocardiogram (corrected QT interval)	X	X	X		X			X	X		
Height and weight		X							X		
Targeted physical examination[h]		X	X	X	X	X	X	X			
Vital signs: temperature, pulse rate, blood pressure, respiratory rate, SpO_2 and FiO_2	X	X	X	X	X	X	X	X	X		X
Placement of central venous catheter and PiCCO catheter	X										
EVLWi and PVPi		X	X	X	X	X	X	X			
Mechanical ventilation parameters[i]		X	X		X			X	X		X
Arterial blood gas		X	X		X			X	X		
SOFA score and OI[j]		X	X		X			X	X		X
Clinical status[k]		X	X	X	X	X	X	X	X		X
PK sampling		X[l]	X[m]		X[m]			X[m]			
Blood, plasma and serum sampling for biomarker studies		X	X		X			X	X		
Thoracic ultrasound (optional)		X			X						
Targeted medication review (including use of vasopressors)		X	X	X	X	X	X	X	X		X

Study Period	Screening	Baseline	Treatment							Follow up	
**Study Day (± Window)**	**0-1[(a)]**	**1**	**2**	**3**	**4**	**5**	**6**	**7**	**10**	**11-27[(b)]**	**28[(c)] (±3)**
Adverse event evaluation		X	X	X	X	X	X	X	X	X	X
SAFETY LABORATORY											
Haematology, chemistry, liver function tests, coagulation	X[(n)]	X	X		X			X	X[(o)]		
Pregnancy test for females of childbearing potential	X										

EVLWi =Extravascular lung water index; FiO_2=Fraction of inspired oxygen; PaO_2=partial pressure of oxygen; SARS-CoV-2= severe acute respiratory syndrome coronavirus 2; SoC=standard of care; SpO2 = peripheral oxygen saturation; OI = Oxygenation Index PVPi = pulmonary vascular permeability index; SOFA score = Sequential Organ Failure Assessment score.

(a). To be performed prior to randomisation.

(b). Or until hospital discharge, if earlier.

(c). If patient has been discharged before Day 28, this assessment may be conducted by telephone or with a home visit by study staff. For visits conducted by telephone, it will not be possible to perform some scheduled assessments (e.g., ECG). Where patients have discontinued the study prematurely, Day 28 assessments should be performed, where possible.

(d). Medical history includes an estimate of date and time of first signs and symptoms and presence of co-morbidities (e.g., respiratory, cardiovascular, metabolic, malignancy, endocrine, gastrointestinal, immunologic, renal).

(e). Tests performed prior to hospital admission are acceptable provided test result is from a laboratory or validated point of care test.

(f). Documentation of evidence to confirm diagnosis and ARDS severity according to Berlin definition.

(g). Randomisation and first dose of IMP must take place within 48 hours of intubation. IMP should be administered twice daily, 12h (± 2h) apart between 06:00 - 12:00 in the morning and 18:00 - 24:00 in the evening. Exceptions apply on Day 1 – see § 6.2 Dosage and Administration.

(h). Where clinically indicated.

(i). Air driving pressure; respiratory system compliance, PEEP, Tidal volume, and mechanical power.

(j). OI: Assess once daily, in case of more measurements per calendar day, the worst OI, and all related measurements will be taken. Manual techniques should be used only if automated devices are not available.

(k). Record any change in clinical status: change in extubation or reintubation; first unassisted breathing or death; discharge from ICU, hospital or death; WHO ordinal Scale for Clinical Improvement.

(l). Plasma samples for determination of imatinib, albumin and AGP taken at 4h and between 7 and 8h after the start of the first IMP infusion. Where PK sampling cannot be performed on Day 1, samples will be taken 3h and 7h after the start of the first IMP infusion on Day 2 instead. For a subgroup of patients, extra samples will be taken during the IMP infusion and 2h after the start of the first infusion (i.e., at the end of the infusion).

(m). Pre-IMP infusion (am or pm dose).

(n). Laboratory tests performed in the 48 hours prior to first dose of study treatment will be accepted for determination of eligibility. If multiple tests are performed during this time, the test closest to dosing will be regarded as the formal sample to confirm eligibility.

(o). Any laboratory tests performed as part of routine clinical care within ±1 day of Day 10 assessment while hospitalized can be used.

DocuSign

Certificate Of Completion

Envelope Id: E0BA0CA7ACF847CCB3047BE0B4F351AD
Status: Completed
Subject: Please DocuSign: EXVASTAT SAP Final 1.0 24052022.docx
Source Envelope:
Document Pages: 90
Signatures: 4
Certificate Pages: 6
Initials: 0
AutoNav: Enabled
EnvelopeId Stamping: Disabled
Time Zone: (UTC) Dublin, Edinburgh, Lisbon, London

Envelope Originator:
Richard Morton
Unit 29 Simbec Research Ltd
Merthyr Tydfil Industrial Park, Pentrebach
Merthyr Tydfil , Merthyr Tydfil CF464DR
Richard.Morton@simbecorion.com
IP Address: 51.219.87.100

Record Tracking

Status	Holder	Location
Status: Original<br>24-May-2022 | 17:23	Holder: Richard Morton<br>Richard.Morton@simbecorion.com	Location: DocuSign

Signer Events

Signer Events	Signature	Timestamp
christelle grall<br>Christelle.Grall@simbecorion.com<br>Project Director<br>Orion Clinical Services Limited (acting for itself and on behalf of its Affiliates) - Part 11<br>Security Level: Email, Account Authentication (Required)	christelle grall<br><br>Signature Adoption: Pre-selected Style<br>Signed by link sent to Christelle.Grall@simbecorion.com<br>Signature ID:<br>DD4B2DF6-5A62-41C1-95A2-ABDAC81AE309<br>Using IP Address: 51.219.87.100<br><br>With Signing Authentication via DocuSign password<br>With Signing Reasons (on each tab):<br>I approve this document	Sent: 24-May-2022 | 17:36<br>Viewed: 24-May-2022 | 19:47<br>Signed: 24-May-2022 | 19:48

Electronic Record and Signature Disclosure:
Not Offered via DocuSign

Signer Events	Signature	Timestamp
james McGinlay<br>James.McGinlay@simbecorion.com<br>Simbec Orion<br>Security Level: Email, Account Authentication (Required)	james McGinlay<br><br>Signature Adoption: Pre-selected Style<br>Signed by link sent to James.McGinlay@simbecorion.com<br>Signature ID:<br>D4E896BE-86BE-4398-A102-48CE7C0804EB<br>Using IP Address: 51.219.87.100<br><br>With Signing Authentication via DocuSign password<br>With Signing Reasons (on each tab):<br>I approve this document	Sent: 24-May-2022 | 17:36<br>Viewed: 25-May-2022 | 09:24<br>Signed: 25-May-2022 | 09:25

Electronic Record and Signature Disclosure:
Not Offered via DocuSign

Signer Events	Signature	Timestamp
Jurjan Aman<br>j.aman@amsterdamumc.nl<br>MD PhD<br>Jurjan Aman<br>Security Level: Email, Account Authentication (Required)	Jurjan Aman<br><br>Signature Adoption: Pre-selected Style<br>Signed by link sent to j.aman@amsterdamumc.nl<br>Signature ID:<br>3BE720AB-1161-4B42-B078-9CDBD0B82B36<br>Using IP Address: 143.176.157.246<br><br>With Signing Authentication via DocuSign password<br>With Signing Reasons (on each tab):<br>Ik keur dit document goed	Sent: 24-May-2022 | 17:36<br>Viewed: 24-May-2022 | 18:47<br>Signed: 24-May-2022 | 19:09
**Electronic Record and Signature Disclosure:**<br>Accepted: 25-Jan-2021 | 20:37<br>ID: 81908d77-d7f5-4337-a24c-8644a4547532		
Richard Morton<br>richard.morton@simbecorion.com<br>Simbec Orion<br>Security Level: Email, Account Authentication (Required)	Richard Morton<br><br>Signature Adoption: Pre-selected Style<br>Signed by link sent to<br>richard.morton@simbecorion.com<br>Signature ID:<br>64790F34-AA68-425C-849F-940546C5FB03<br>Using IP Address: 51.219.87.100<br><br>With Signing Authentication via DocuSign password<br>With Signing Reasons (on each tab):<br>I approve this document	Sent: 24-May-2022 | 17:36<br>Viewed: 24-May-2022 | 17:36<br>Signed: 24-May-2022 | 17:37
**Electronic Record and Signature Disclosure:**<br>Not Offered via DocuSign		

In Person Signer Events	Signature	Timestamp

Editor Delivery Events	Status	Timestamp

Agent Delivery Events	Status	Timestamp

Intermediary Delivery Events	Status	Timestamp

Certified Delivery Events	Status	Timestamp

Carbon Copy Events	Status	Timestamp

Witness Events	Signature	Timestamp

Notary Events	Signature	Timestamp

Envelope Summary Events	Status	Timestamps
Envelope Sent	Hashed/Encrypted	24-May-2022 | 17:36
Certified Delivered	Security Checked	24-May-2022 | 17:36
Signing Complete	Security Checked	24-May-2022 | 17:37
Completed	Security Checked	25-May-2022 | 09:25

Payment Events	Status	Timestamps

Electronic Record and Signature Disclosure

ELECTRONIC RECORD AND SIGNATURE DISCLOSURE

From time to time, Orion Clinical Services Limited (acting for itself and on behalf of its Affiliates) - Part 11 (we, us or Company) may be required by law to provide to you certain written notices or disclosures. Described below are the terms and conditions for providing to you such notices and disclosures electronically through the DocuSign system. Please read the information below carefully and thoroughly, and if you can access this information electronically to your satisfaction and agree to this Electronic Record and Signature Disclosure (ERSD), please confirm your agreement by selecting the check-box next to 'I agree to use electronic records and signatures' before clicking 'CONTINUE' within the DocuSign system.

Getting paper copies

At any time, you may request from us a paper copy of any record provided or made available electronically to you by us. You will have the ability to download and print documents we send to you through the DocuSign system during and immediately after the signing session and, if you elect to create a DocuSign account, you may access the documents for a limited period of time (usually 30 days) after such documents are first sent to you. After such time, if you wish for us to send you paper copies of any such documents from our office to you, you will be charged a $0.00 per-page fee. You may request delivery of such paper copies from us by following the procedure described below.

Withdrawing your consent

If you decide to receive notices and disclosures from us electronically, you may at any time change your mind and tell us that thereafter you want to receive required notices and disclosures only in paper format. How you must inform us of your decision to receive future notices and disclosure in paper format and withdraw your consent to receive notices and disclosures electronically is described below.

Consequences of changing your mind

If you elect to receive required notices and disclosures only in paper format, it will slow the speed at which we can complete certain steps in transactions with you and delivering services to you because we will need first to send the required notices or disclosures to you in paper format, and then wait until we receive back from you your acknowledgment of your receipt of such paper notices or disclosures. Further, you will no longer be able to use the DocuSign system to receive required notices and consents electronically from us or to sign electronically documents from us.

All notices and disclosures will be sent to you electronically

Unless you tell us otherwise in accordance with the procedures described herein, we will provide electronically to you through the DocuSign system all required notices, disclosures, authorizations, acknowledgements, and other documents that are required to be provided or made available to you during the course of our relationship with you. To reduce the chance of you inadvertently not receiving any notice or disclosure, we prefer to provide all of the required notices and disclosures to you by the same method and to the same address that you have given us. Thus, you can receive all the disclosures and notices electronically or in paper format through the paper mail delivery system. If you do not agree with this process, please let us know as described below. Please also see the paragraph immediately above that describes the consequences of your electing not to receive delivery of the notices and disclosures electronically from us.

How to contact Orion Clinical Services Limited (acting for itself and on behalf of its Affiliates) - Part 11:

You may contact us to let us know of your changes as to how we may contact you electronically, to request paper copies of certain information from us, and to withdraw your prior consent to receive notices and disclosures electronically as follows:
To contact us by email send messages to: jim.kendall@simbecorion.com

To advise Orion Clinical Services Limited (acting for itself and on behalf of its Affiliates) - Part 11 of your new email address

To let us know of a change in your email address where we should send notices and disclosures electronically to you, you must send an email message to us at jim.kendall@simbecorion.com and in the body of such request you must state: your previous email address, your new email address. We do not require any other information from you to change your email address.

If you created a DocuSign account, you may update it with your new email address through your account preferences.

To request paper copies from Orion Clinical Services Limited (acting for itself and on behalf of its Affiliates) - Part 11

To request delivery from us of paper copies of the notices and disclosures previously provided by us to you electronically, you must send us an email to jim.kendall@simbecorion.com and in the body of such request you must state your email address, full name, mailing address, and telephone number. We will bill you for any fees at that time, if any.

To withdraw your consent with Orion Clinical Services Limited (acting for itself and on behalf of its Affiliates) - Part 11

To inform us that you no longer wish to receive future notices and disclosures in electronic format you may:

i. decline to sign a document from within your signing session, and on the subsequent page, select the check-box indicating you wish to withdraw your consent, or you may;

ii. send us an email to jim.kendall@simbecorion.com and in the body of such request you must state your email, full name, mailing address, and telephone number. We do not need any other information from you to withdraw consent.. The consequences of your withdrawing consent for online documents will be that transactions may take a longer time to process..

Required hardware and software

The minimum system requirements for using the DocuSign system may change over time. The current system requirements are found here: https://support.docusign.com/guides/signer-guide-signing-system-requirements.

Acknowledging your access and consent to receive and sign documents electronically

To confirm to us that you can access this information electronically, which will be similar to other electronic notices and disclosures that we will provide to you, please confirm that you have read this ERSD, and (i) that you are able to print on paper or electronically save this ERSD for your future reference and access; or (ii) that you are able to email this ERSD to an email address where you will be able to print on paper or save it for your future reference and access. Further, if you consent to receiving notices and disclosures exclusively in electronic format as described herein, then select the check-box next to 'I agree to use electronic records and signatures' before clicking 'CONTINUE' within the DocuSign system.

By selecting the check-box next to 'I agree to use electronic records and signatures', you confirm that:

- You can access and read this Electronic Record and Signature Disclosure; and
- You can print on paper this Electronic Record and Signature Disclosure, or save or send this Electronic Record and Disclosure to a location where you can print it, for future reference and access; and
- Until or unless you notify Orion Clinical Services Limited (acting for itself and on behalf of its Affiliates) - Part 11 as described above, you consent to receive exclusively through electronic means all notices, disclosures, authorizations, acknowledgements, and other documents that are required to be provided or made available to you by Orion Clinical Services Limited (acting for itself and on behalf of its Affiliates) - Part 11 during the course of your relationship with Orion Clinical Services Limited (acting for itself and on behalf of its Affiliates) - Part 11.
